# A Pilot Study of IL-2Rα Blockade during Lymphopenia Depletes Regulatory T-cells and Correlates with Enhanced Immunity in Patients with Glioblastoma

**DOI:** 10.1371/journal.pone.0031046

**Published:** 2012-02-27

**Authors:** John H. Sampson, Robert J. Schmittling, Gary E. Archer, Kendra L. Congdon, Smita K. Nair, Elizabeth A. Reap, Annick Desjardins, Allan H. Friedman, Henry S. Friedman, James E. Herndon, April Coan, Roger E. McLendon, David A. Reardon, James J. Vredenburgh, Darell D. Bigner, Duane A. Mitchell

**Affiliations:** 1 Division of Neurosurgery, Department of Surgery, Duke University Medical Center, Durham, North Carolina, United States of America; 2 Department of Pathology, Duke University Medical Center, Durham, North Carolina, United States of America; 3 Division of Surgical Sciences, Department of Surgery, Duke University Medical Center, Durham, North Carolina, United States of America; 4 Department of Biostatistics and Bioinformatics, Duke University Medical Center, Durham, North Carolina, United States of America; 5 Dana-Farber Cancer Institute, Boston, Massachusetts, United States of America; The University of Chicago, United States of America

## Abstract

**Background:**

Preclinical studies in mice have demonstrated that the prophylactic depletion of immunosuppressive regulatory T-cells (T_Regs_) through targeting the high affinity interleukin-2 (IL-2) receptor (IL-2Rα/CD25) can enhance anti-tumor immunotherapy. However, therapeutic approaches are complicated by the inadvertent inhibition of IL-2Rα expressing anti-tumor effector T-cells.

**Objective:**

To determine if changes in the cytokine milieu during lymphopenia may engender differential signaling requirements that would enable unarmed anti-IL-2Rα monoclonal antibody (MAbs) to selectively deplete T_Regs_ while permitting vaccine-stimulated immune responses.

**Methodology:**

A randomized placebo-controlled pilot study was undertaken to examine the ability of the anti-IL-2Rα MAb daclizumab, given at the time of epidermal growth factor receptor variant III (EGFRvIII) targeted peptide vaccination, to safely and selectively deplete T_Regs_ in patients with glioblastoma (GBM) treated with lymphodepleting temozolomide (TMZ).

**Results and Conclusions:**

Daclizumab treatment (n = 3) was well-tolerated with no symptoms of autoimmune toxicity and resulted in a significant reduction in the frequency of circulating CD4+Foxp3+ TRegs in comparison to saline controls (n = 3)( p = 0.0464). A significant (p<0.0001) inverse correlation between the frequency of TRegs and the level of EGFRvIII specific humoral responses suggests the depletion of TRegs may be linked to increased vaccine-stimulated humoral immunity. These data suggest this approach deserves further study.

**Trial Registration:**

ClinicalTrials.gov NCT00626015

## Introduction

CD4^+^CD25^+^Foxp3^+^ regulatory T-cells (T_Regs_) are an immunosuppressive lymphocyte subset comprising 5–10% of the CD4^+^ compartment in both mice and humans [1]. T_Regs_ potently inhibit T-cell cytokine secretion and proliferation [2]–[6], directly curtail the generation and expansion of endogenous or induced immune responses [7]–[16], and appear to play a significant role in hindering immunity to normal and tumor-associated antigens [17], [18]. Increased levels of T_Regs_ have been found in the tumors and peripheral blood of patients with various malignancies including glioblastoma multiforme (GBM), and within GBM, we have shown T_Regs_ to be an important and reversible component of the immunosuppression endemic to this disease [19]–[23].Early attempts to clinically deplete T_Regs_ and alleviate anti-tumor immunosuppression targeted the high affinity interleukin-2 (IL-2) Receptor (IL-2Rα/CD25) due to its constitutive expression on the T_Reg_ population. Denileukin diftitox, a fusion protein of IL-2 and a portion of the diphtheria toxin, and LMB-2, a fusion protein of an anti-IL-2Rα MAb and a portion of a bacterial exotoxin, have been utilized in humans to deplete T_Regs_ but have achieved inconsistent successes in improving immunotherapy [24]–[27]; potentially because activated effector T-cells transiently express IL-2Rα [28]. Unarmed anti-IL-2Rα antibodies that block IL-2 signaling [29], as opposed to cytolytic targeted therapies, have the potential to act differentially upon T-cells depending on their requirement for IL-2. Additionally, work from our laboratory [30] and others [31] has shown in murine models that anti-IL-2Rα MAbs can deactivate T_Reg_ suppression through functional inhibition as well as depletion.

A recent report by Jacobs *et al.* [32] examined the ability of the humanized anti-IL-2Rα MAb daclizumab to deplete T_Regs_ in metastatic melanoma patients receiving antitumor vaccination in the absence of chemotherapy. They demonstrated that while T_Regs_ were effectively depleted, the functionality of vaccine-induced anti-tumor T-cells was impaired and the formation of vaccine-induced humoral immunity was blocked; suggesting that daclizumab will impair both T_Regs_ and effector T-cell activation. However, administration of anti-IL-2Rα MAb during lymphopenia may function differently than in a normal non-lymphopenic context due to disparate IL-2 signaling requirements by regulatory versus effector T-cells. Preclinical studies in our lab corroborate this hypothesis as anti-IL-2Rα MAb administration during temozolomide (TMZ) induced lymphopenia depletes T_Regs_ while sparing activated effectors to enhance anti-tumor efficacy in an established model of murine tumorigenesis [33]. Therefore, we believe that the application of anti-IL-2Rα MAbs during standard chemotherapy-induced lymphopenia in patients with cancer will selectively ablate or inactivate T_Regs_ while permitting immune responses induced by anti-tumor immunotherapy.

**Figure 1:**
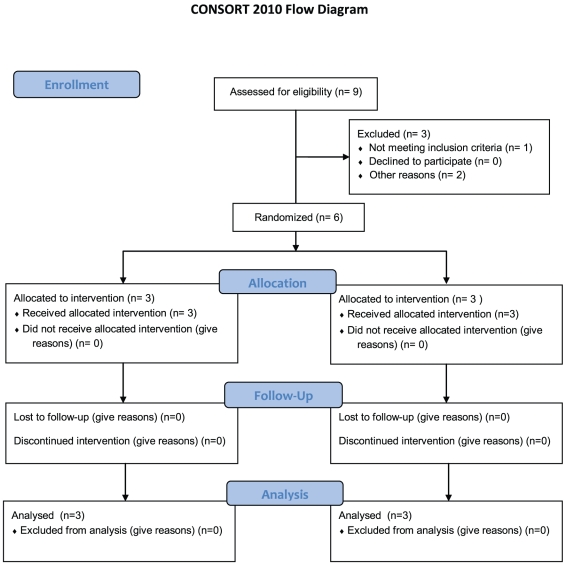
CONSORT 2010 Flow Diagram. Flow diagram of enrollment, allocation, follow-up and analysis of the Zenapax® Activated Peptide Immunotherapy (ZAP IT) Clinical Trial.

We herein report that a single dose of the anti-IL-2Rα MAb daclizumab, given concomitant with epidermal growth factor receptor variant III (EGFRvIII) targeted vaccination in a randomized saline-controlled pilot study, has the capacity to safely and effectively deplete CD4^+^Foxp3^+^ T_Regs_ in TMZ-treated patients with GBM without impairing vaccine-induced immune responses (Figure 1). EGFRvIII is a tumor-specific mutation commonly found on GBMs [34] as well as breast, lung, head and neck cancers [35]–[37] and studies from our group demonstrate that peptide vaccination targeting the mutant fusion junction of EGFRvIII prolongs survival time in a selected population of patients with GBM treated in multi-institutional Phase II trials [38]–[40]. Daclizumab given concomitantly with EGFRvIII-targeted vaccination significantly depleted T_Regs_ (p = 0.0464) without impairing EGFRvIII specific antibody titers. Additionally, vaccine-stimulated anti-EGFRvIII antibody levels showed a significant inverse correlation with the frequency of T_Regs_ (r = −0.93, p<0.0001). The cumulative data suggests that administration of an anti-IL-2Rα MAb during lymphopenia is not only safe, but that it notably reduces T_Regs_ allowing enhanced vaccine-stimulated immunity.

## Results

### In vitro impact of daclizumab on effector T-cell function

**Figure 2:**
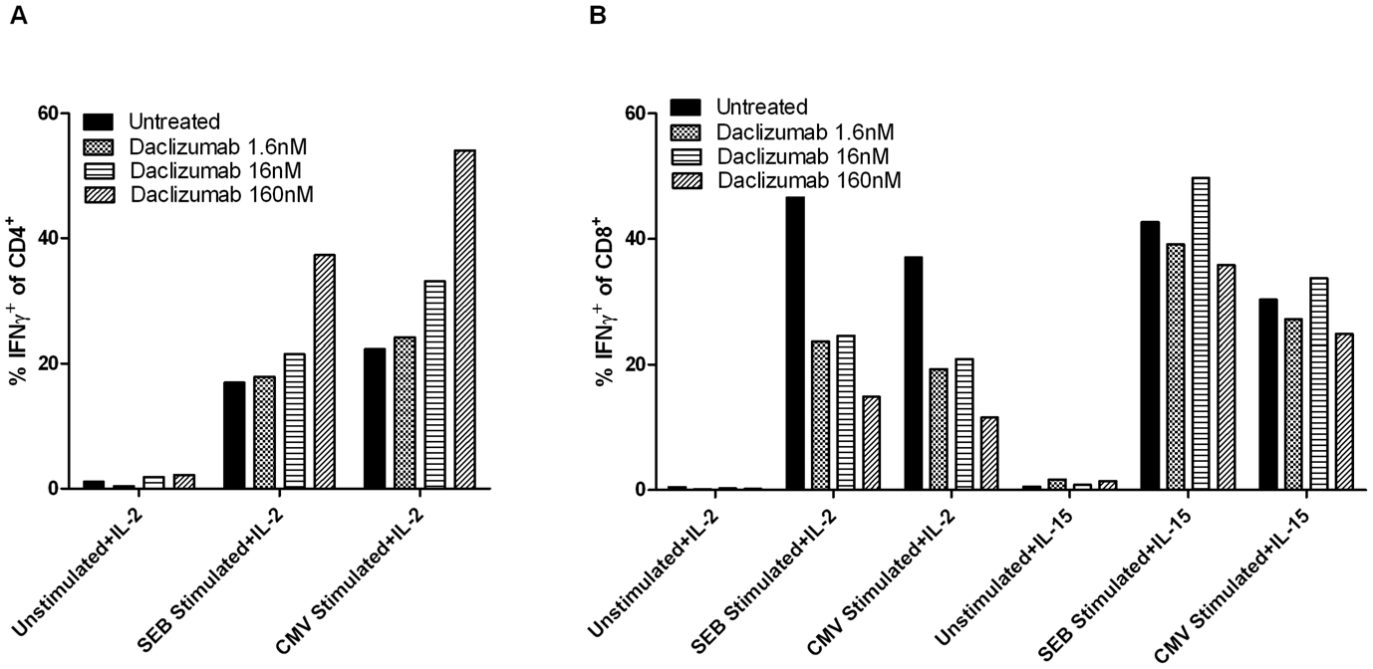
*In vitro* effects of IL2Rα inhibition on CD4^+^, CD8^+^ and regulatory T-cells. Normal donor peripheral blood mononuclear cells (PBMCs) were cultured for 48 hours with increasing concentrations of daclizumab followed by an additional 14 days stimulation/expansion with CMV pp65 RNA-pulsed DCs along with IL-2 or IL-15. PBMC were then isolated and stimulated for 6 hours with SEB or pp65 peptide mix in the presence of CD28/CD49d costimulation and Brefeldin A. The IFN-γ secretion of (A) CD3^+^CD4^+^CD69^+^ or (B) CD3^+^CD8^+^CD69^+^ T-cells was determined by flow cytometry.

Daclizumab is a humanized MAb that specifically binds to the high affinity IL-2 receptor and blocks IL-2 binding [41]. IL-2Rα inhibition mediated by daclizumab has been shown to inconsistently inhibit T-cell function *in vitro* [29], [42]. To begin assessing the functionality of CD4^+^ and CD8^+^ effector T-cells exposed to anti-IL-2Rα MAbs, we performed a two week *in vitro* activation with dendritic cells (DCs) expressing the immunodominant *Cytomegalovirus* (CMV) pp65 protein, a model human antigen, in the presence of increasing concentrations of daclizumab (Figure 2A & 2B). As a marker of functionality, T-cells were examined for the secretion of interferon-gamma (IFN-γ) after stimulation with the superantigen SEB or restimulation with CMV pp65 peptide mix. The secretion of IFNγ by CD4^+^ T-cells stimulated with CMV or SEB was enhanced by increasing doses of daclizumab. While increasing doses of daclizumab diminished IFN-γ secretion by CD8^+^ T-cells; IFN-γ secretion could be rescued in the presence of interleukin 15 (IL-15). Importantly, IL-15 bioavailability is increased during lymphopenia induced homeostatic proliferation [43] and our *in vitro* data in combination with other preclinical studies from our laboratory supports the possibility that daclizumab may well function differentially on effector T-cells and T_Regs_ *in vivo* during TMZ induced lymphopenia.

### Clinical Trial

**Figure 3:**
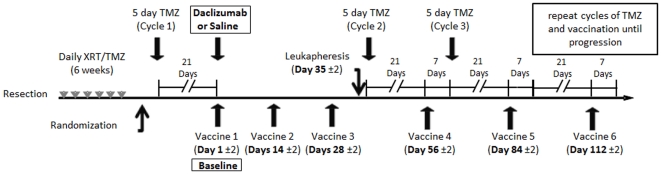
Schema of ZAP IT Trial.

To begin assessing the potential of a single dose of daclizumab, a clinically-approved αIL-2Rα MAb, to reduce or eliminate T_Regs_ in lymphopenic patients with newly-diagnosed GBM undergoing standard-of-care TMZ therapy (Zenapax-Activated Peptide ImmunoTherapy (ZAP IT) Protocol - FDA - IND - BB - 9949, Duke IRB Pro00000947); six patients with EGFRvIII-expressing GBM were treated with standard of care radiation with TMZ therapy and then randomized in a double-blinded fashion to saline (n = 3) or daclizumab (n = 3). With an original accrual goal of 20 patients, enrollment on this trial was halted after six patients due to discontinuation of the availability of daclizumab by the manufacturer. Patients began the first cycle of 200 mg/m^2^ TMZ for 5 days and on day 21±2 concomitantly received the PEPvIII peptide EGFRvIII-targeted vaccine [39] and a single infusion of daclizumab (1 mg/kg) or saline. Extensive work from our laboratory has shown that PEPvIII peptide vaccination elicits potent and predominantly humoral responses generating high levels of anti-PEPvIII specific antibodies [38]–[40]. Patient characteristics and a schematic of the ZAP IT study are summarized in Table 1 and Figure 3 respectively. All enrolled patients, were randomized and included in the study analysis.

**Table 1:** ZAP IT Patient Characteristics.

Patient	Treatment	Gender	Age	EGFRvIII Intensity (+ to 3+)	TMZ Cycles	KPS
1	Daclizumab	F	64	3+	12	100
2	Daclizumab	M	78	3+	12	80
3	Saline	M	50	2–3+	>12	100
4	Saline	M	60	3+	12	90
5	Daclizumab	M	55	2+	12	90
6	Saline	F	35	2+	3	90

> As of the lock date of the data, the indicated patient had 12 cycles of TMZ but continued with additional cycles of TMZ treatment.

### A single infusion of daclizumab in the context of immunization is safe

Given that this trial may be establishing new treatment regimens with the potential for an increased risk of toxicity, patients were clinically assessed before each vaccination and a panel of clinical laboratory analyses were additionally performed to screen for the most common manifestations of autoimmunity seen in related trials [44]–[47]. Daclizumab administration in the context of EGFRvIII-targeted immunization was well-tolerated with no adverse events beyond itching, swelling and redness at the vaccination site attributable to the vaccine and no changes in autoimmune laboratory analyses relative to baseline in daclizumab or saline treated individuals. For each patient, the average percent change between baseline (i.e. vaccine 1) and vaccine 4, 5, and 6 time points was computed for cortisol, TSH and ACTH. A two-sample t-test comparison of the daclizumab and saline groups with respect to these outcomes demonstrated no evidence of a difference (p = 0.4229, p = 0.5653, p = 0.3795, respectively).

### Daclizumab administration depletes T_Regs_ and increases the effector T-cell to T_Reg_ Ratio

**Figure 4:**
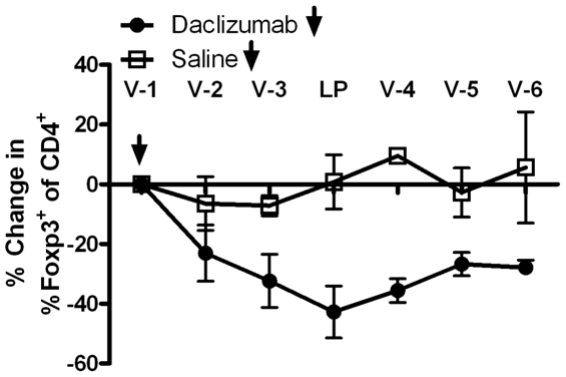
Regulatory T-cells are significantly depleted by a single infusion of daclizumab. The frequency of CD4^+^Foxp3^+^ T_Regs_ was determined by FACS analysis of peripheral blood samples drawn prior to vaccination (V) or leukapheresis (LP). Percent change was calculated in comparison to baseline (vaccine 1). For each follow-up assessment, percent change from baseline (vaccine 1) was computed. For statistical comparisons of the daclizumab and saline groups, the average percent change at vaccine 2 (V-2), vaccine 3 (V-3), and leukapheresis (LP) was computed for each patient. Daclizumab showed a significantly greater reduction in CD4^+^Foxp3^+^ regulatory T-cells (p = 0.0464).

The frequency of patient CD4^+^, CD8^+^ and CD4^+^Foxp3^+^ regulatory T-cells were monitored by complete blood counts (CBC) and flow cytometric analysis over the course of treatment. Throughout this study, T_Regs_ were defined as CD4^+^Foxp3^+^ as opposed to CD4^+^CD25^+^Foxp3^+^ as the binding of daclizumab to CD25 can impair the antibody-mediated flow cytometric detection of CD25 and may lead to subjectivity in determining the T_Reg_ population. For each patient, the average percent change in the frequency of CD4^+^Foxp3^+^ regulatory T-cells between baseline and the vaccine 2, 3 and leukapheresis time points was calculated. A two-sample t-test comparison showed that a single infusion of daclizumab resulted in a significant reduction in circulating T_Regs_ (p = 0.0464; Figure 4). For the observed time-points, T_Regs_ reached a nadir approximately one month after daclizumab administration and had not returned to baseline levels by vaccine 6 (day 112±2). The impact of daclizumab on the percent change in the frequency of CD8^+^ and overall CD4^+^ T-cells was also examined and showed considerable inter-patient variability, demonstrating no average trend in either direction as opposed to the notable decrease that was seen in the T_Reg_ population after infusion with daclizumab. Therefore, daclizumab administration significantly reduces T_Regs_ in TMZ treated patients with GBM with no evidence of a corresponding depletion of CD4^+^ or CD8^+^ T-cells.

To determine if daclizumab had any impact on the proliferative capacity of effector T-cells or T_Regs_, cells were analyzed for Ki67 status by flow cytometric analysis at baseline, leukapheresis and vaccine 4 time-points. No difference in the average percent change from baseline to leukapheresis and vaccine 4 between the daclizumab and saline groups with respect to CD4^+^ and CD8^+^ T-cells was detected (p = 0.4947 and p = 0.8113, respectively), suggesting that unarmed MAb blockade of IL-2Rα may not impair effector T-cell expansion. Though not statistically significant, there is a trend towards the daclizumab group having a smaller percent reduction in the average percent change from baseline in the frequency of Ki67^+^T_Regs_ relative to the saline group (p = 0.0841). This may simply be indicative of the regenerating T_Reg_ population rebounding in response to daclizumab-mediated depletion [48], [49].

As anti-IL-2Rα MAb administration has been shown to suppress vaccine-induced immunity [28], [32], the activation status of CD4^+^ T-cells was also assessed by examining HLA-DR expression. No difference in HLA-DR expression on CD4^+^ T-cells in daclizumab treated patients relative to controls was found (p = 0.4861); suggesting that daclizumab may not impair CD4^+^ effector T-cell activation.

**Figure 5:**
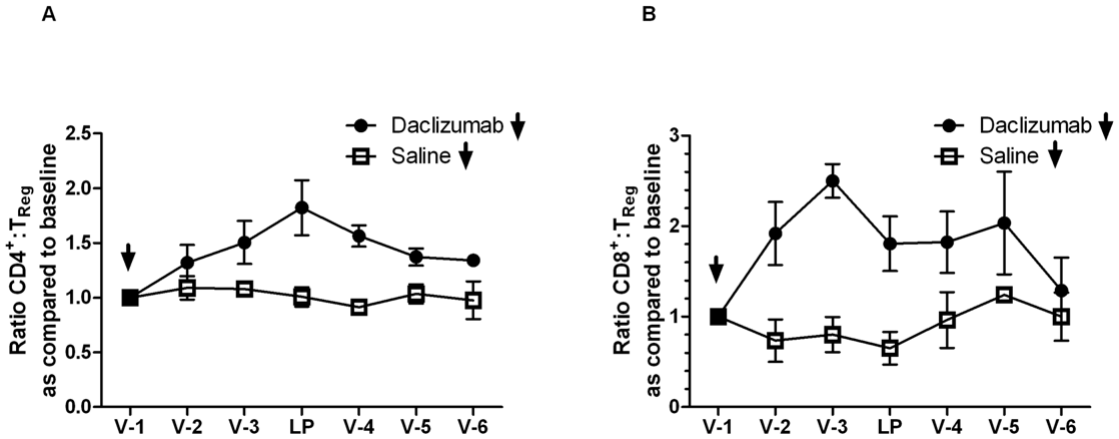
*In vivo* effects of daclizumab on the effector T-cell to regulatory T-cell ratio. (A–B) Effector T-cells (CD4^+^ or CD8^+^) to T_Reg_ ratios were derived by dividing the absolute number of effector T-cells by the absolute number of T_Regs_ at the indicated time-points; the absolute number of cells was determined by a combination of CBC and FACS analysis. The ratios of effector T-cells to T_Regs_ as compared to baseline were generated by dividing the individual patient CD4^+^∶T_Reg_ or CD8^+^∶T_Reg_ ratio at every time point by the ratio at vaccine 1 (V-1 = baseline). A two sample t-test averaged over the V-2, V-3 and LP time-points was utilized to examine the difference between the daclizumab and saline groups in the CD4^+^∶T_Reg_ (p = 0.0757) and CD8^+^∶T_Reg_ (p = 0.0153) ratios.

Preclinical and clinical studies show that increased systemic and intratumoral ratios of effector T-cells to T_Regs_ are associated with favorable cancer prognoses and enhanced anti-tumor efficacy after immunotherapy [50]–[54]. To assess the impact of daclizumab treatment on the ratio of CD4^+^ and CD8^+^ effector T-cells to T_Regs_, the absolute number of T-cells was divided by the absolute number of T_Regs_ and this ratio was compared to the ratio at baseline (Figure 5). A two sample t-test of this outcome averaged over the V-2, V-3 and LP time-points was generated to assess any statistical difference between daclizumab and saline treated patients. Both the CD4^+^∶T_Reg_ and the CD8^+^∶T_Reg_ ratios were notably altered in the arm receiving daclizumab as compared to saline controls with the ratio of CD4^+^∶T_Regs_ trending towards significance and the ratio of CD8^+^∶T_Regs_ demonstrating a significant increase in the average percent change from baseline (p = 0.0757 and p = 0.0153, respectively). The enhancement of effector to T_Reg_ ratios after daclizumab infusion suggests that daclizumab administration in TMZ treated patients with GBM may create an environment conducive to immunotherapeutic intervention. In contrast to preclinical [28] and clinical studies [32] examining MAb mediated inhibition of IL-2Rα in non-lymphopenic individuals, our cumulative data suggests that in TMZ treated patients with GBM, a single infusion of daclizumab effectively depletes T_Regs_ without notably impacting the CD4^+^ or CD8^+^ effector T-cell compartments.

### Daclizumab administration and regulatory natural killer cells

Regulatory CD56^Bright^ natural killer (NK) cells have been shown to both be expanded by the addition of daclizumab and to indirectly mediate the inhibitory effects of αIL-2Rα MAbs on effector T-cells [42], [55]. For each patient, the average percent change between baseline, leukapheresis and vaccine 4 time points was computed for the frequency of regulatory CD3^−^CD56^Bright^CX3CR1^−^ NK cells. No difference between daclizumab and saline groups was detected (two sample t-test p = 0.7088). It is possible that in this lymphodepleted context, daclizumab expanded regulatory NK cells that might normally impair effector T-cells are not present and therefore anti-IL-2Rα MAbs would generate a selective impairment on the T_Regs_ population.

### Daclizumab remains bound to residual T_Regs_

**Figure 6:**
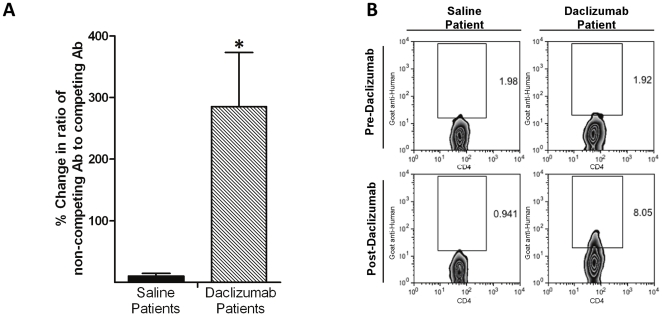
Daclizumab remains bound to T_Regs_ a month after administration. (A) CD4^+^Foxp3^+^ T_Regs_ from day 35±2 leukapheresis samples (saline n = 3, daclizumab n = 3) were determined by flow cytometry and were additionally stained with anti-CD25 antibodies that bind the same CD25 eptiope as daclizumab (competing clone 2A3) or bind a separate epitope (non-competing clone MA251). The ratio of the frequency of CD4^+^CD25^+^Foxp3^+^ T_Regs_ as determined by MA251 or 2A3 binding was used as an indirect indicator of surface daclizumab expression. The percent change in the ratio was calculated from ratios determined from baseline (vaccine 1) samples, unpaired t-test *p = 0.0353. (B) CD4^+^CD25^+^Foxp3^+^ T_Regs_ were determined by FACS analysis of PBMC and examined for human antibody expression as a direct indicator of daclizumab binding to the surface of T_Regs_. PBMCs from a saline and a daclizumab treated patient from vaccine 1 (Pre-Daclizumab) and leukapheresis at day 35±2 (Post-Daclizumab) time-points were assessed.

The half-life of daclizumab is 20 days [56] and it has been indirectly shown that daclizumab can remain bound to T_Regs_ for weeks after administration [42], [57]. To determine if daclizumab remains bound to residual T_Regs_ in TMZ treated patients with GBM, T_Regs_ were isolated both before and after saline or daclizumab administration and identical samples were separately stained for the frequency of CD4^+^CD25^+^Foxp3^+^ T_Regs_ with an anti-CD25 antibody that recognizes the Tac epitope (competing with daclizumab, clone 2A3) and one that does not (non-competing, clone MA251). The ratio of T_Regs_ (non-competing antibody/competing antibody) was determined and a ratio greater than 1 indicates a T_Reg_ population that is only detectable by the non-competing antibody. This demonstrates that detection of T_Regs_ by the competing antibody is blocked by the presence of daclizumab bound to IL-2Rα. The percent change in this ratio from baseline (vaccine 1) to leukapheresis (35 days after daclizumab) was used to determine the amount of daclizumab remaining bound to the T_Regs_. An increase in percent change indicates an increase in the ratio from baseline, demonstrating that 35 days after daclizumab administration there is a population of T_Regs_ detectable only by the non-competing antibody as daclizumab bound to IL-2Rα prevents binding of the competing antibody (Figure 6A). Additionally, to determine if the presence of daclizumab on T_Regs_ could be directly visualized, T_Regs_ were stained with a goat anti-human antibody and the presence of human antibody was detected on T_Regs_ exclusively in the daclizumab treated patient (Figure 6B). These results indicate that daclizumab remains bound to a population of residual T_Regs_ persisting after depletion.

### The frequency of T_Regs_ is inversely correlated with vaccine-stimulated humoral responses

**Figure 7:**
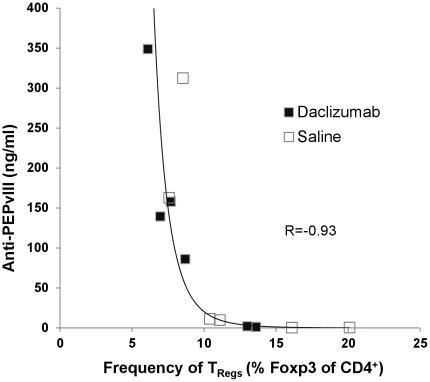
The frequency of T_Regs_ and anti-PEPvIII humoral responses are inversely correlated. Patient sera from peripheral blood (vaccine 4) and leukapheresis samples were analyzed for levels of anti-PEPvIII antibodies and humoral responses were plotted against the frequency of T_Regs_ (Foxp3^+^ of CD4^+^). Assuming the assessments within individuals are independent, the Spearman correlation coefficient for both saline and daclizumab treated patients overall is (R = −0.93, p<0.0001).

Numerous preclinical studies have demonstrated that anti-IL-2Rα MAb administration can deplete or functionally inactivate T_Regs_ in mice and can augment anti-tumor immunotherapy if delivered as a prophylactic prior to vaccination [30], [50], [58]. However, if delivered therapeutically in these models, anti-IL-2Rα MAbs have been shown to impair anti-tumor immune responses potentially due to inhibition of the activated effector T-cells expressing CD25 [28]. Daclizumab administration in non-lymphopenic metastatic melanoma patients significantly depletes T_Regs_ but additionally impaired vaccine-induced anti-tumor T-cell function and prevented vaccine-induced humoral immunity [32]. We have previously demonstrated that the PEPvIII peptide vaccine mediates efficacy through a humoral biased immune response [39] and we examined patients in this study for alterations in humoral immunity. As opposed to the findings of Jacobs *et al.*, no reduction in anti-PEPvIII antibody titers was detected between daclizumab and saline treated patients, indicating daclizumab does not block vaccine-induced humoral immunity in TMZ treated patients with GBM. To examine the potential relationship between the levels of regulatory T-cells and the induction of vaccine-induced antibody responses, we plotted the frequency of T_Regs_ against anti-PEPvIII antibody titers from both saline and daclizumab patients (Figure 7). While our analysis does not demonstrate causality, there was a significant (p<0.0001) inverse correlation (r = −0.93) between the frequency of T_Regs_ and the concentration of anti-PEPvIII antibody, suggesting that high T_Reg_ levels are associated with low anti-PEPvIII antibody responses and low T_Reg_ levels are associated with increased anti-PEPvIII antibody responses. As our cumulative data demonstrates that a single infusion of daclizumab is a safe and effective means of sustained T_Reg_ depletion, this method may be used to reduce T_Regs_ for the augmentation of vaccine-induced immunity as suggested by our heightened anti-PEPvIII antibody titers.

## Discussion

The results presented herein demonstrate that the unarmed IL-2Rα-specific antibody daclizumab effectively eliminates T_Regs_ in TMZ treated patients with GBM without decreasing effector T-cell populations or impairing vaccine-stimulated immunity. *In vitro*, these antibodies enhance IFN-γ production to an antigen-specific and nonspecific stimulus in CD4^+^ T-cells, and while IFN-γ secretion by CD8^+^ T-cells was blunted, this was rescued with the addition of IL-15, a homeostatic cytokine that would be present in patients recovering from chemotherapy-induced lymphopenia. This supports our hypothesis that IL-2Rα-specific antibodies may have differential effects on T_Regs_ and anti-tumor effector T-cells in the milieu of homeostatic cytokines which would be seen in patients treated with lymphodepleting chemotherapies, such as TMZ, which is now standard-of-care for patients with GBM. Enrollment on this study was halted due to unanticipated discontinuation of the availability of daclizumab by the manufacturer. However, when assessed in our randomized saline-controlled pilot study “ZAP-IT”, daclizumab administration was safe, depleted T_Regs_, did not deplete CD4^+^ or CD8^+^ effector T-cells and increased the ratio of CD4^+^ and CD8^+^ effectors to T_Regs_. Importantly, decreased T_Reg_ numbers strongly correlated with heightened vaccine-induced humoral responses, suggesting T_Reg_ depletion may augment vaccine-induced humoral immunity. Finally, unlike other approaches to enhancing immune response in cancer patients [44]–[47], we saw no evidence of toxicity despite the dramatic reduction in regulatory T-cell numbers that we observed.

Other T_Reg_ depletion strategies, such as the IL-2 targeted toxins denileukin diftitox and LMB-2, have been used in clinical studies and have had partial success in the *in vivo* reduction of regulatory T-cells [24]–[26], [59], [60]. However, these strategies have limitations not found when using unarmed MAb blockade. Denileukin diftitox targets the IL-2 moiety itself allowing indiscriminate targeting of the lower affinity IL-2ßγ receptors which are expressed on a broader subset of cells including memory T-cells. Thus, denileukin diftitox cannot distinguish between T_Regs_ and activated and memory T-cells expressing any of the IL-2 receptors and may even stimulate T_Regs_. Alternative strategies that employ IL-2Rα-targeted immunotoxins, such as LMB-2, still allow indiscriminate killing of all IL-2Rα-expressing cells including recently activated, vaccine-induced effector T-cells that express IL-2Rα.

We and others have recently shown, however, that unarmed anti-IL-2Rα antibodies may function differently and not have a direct cytotoxic effect [30], [31]. Rather, these antibodies may impair regulatory T-cells by blocking IL-2 receptor signaling through their cognate receptor. T_Regs_ are known to be uniquely dependent on the high affinity IL-2 receptor for their function and survival [61]–[64] and MAbs that block IL-2Rα have been shown to significantly reduce regulatory T-cell activity in preclinical models [30], [65]–[67]. While these antibodies would also bind IL-2Rα on activated T-cells, activated effectors may not require this signaling, as others have demonstrated that IL-2 signals during priming are required for robust secondary memory T-cell responses and that activated T-cells are not dependent on IL-2 signaling during the primary response [68]. It is additionally quite conceivable that by the time patients are diagnosed and treated, that most tumor antigens actually represent memory T-cells which would not be dependent on IL-2 signaling to generate secondary immune responses. Furthermore, homeostatic cytokines such as IL-7 and IL-15, that are prevalent during lymphopenia, have been shown to be able to substitute for IL-2 signaling in activated effector cells [43]. Thus, when anti-IL-2Rα MAbs are employed in the unique host environment that exists after therapeutic TMZ-induced lymphodepletion, vaccine-stimulated anti-tumor T-cells may be independent of IL-2 signaling whereas T_Regs_ will remain dependent. This differential effect should lead to increased effector∶T_Reg_ ratios as we have seen here, but remains wholly dependent on a lymphodepleted environment. Given the prior difficulties in eliminating T_Regs_ without impairing T-cell effectors, it has been controversial whether or not the depletion of T_Regs_ would enhance immune responses. Our data demonstrating the inverse correlation between T_Reg_ frequency and vaccine-stimulated antibody levels suggests that reducing T_Regs_ may improve vaccine-induced immunity and warrants further investigation. Of note, T_Reg_ depletion using daclizumab was incomplete in these patients using a single intravenous administration, suggesting either incomplete saturation of IL-2Rα receptors at the dose used (1 mg/kg), downregulation or shedding of IL-2Rα from the surface of T_Regs_ that renders some cells refractory to an antibody dependent elimination, or a refractory population of FOXP3+ CD4+ T_Regs_ that is not amenable to elimination by anti-IL-2Rα MAbs treatment. We did not differentiate in this study, for instance, whether thymic-derived natural T_Regs_, (nT_Regs_) versus peripherally converted T_Regs_ are preferentially depleted by anti-IL-2Rα blockade. This is of importance since, recent studies have demonstrated that thymic-derived T_Regs_ predominate in patients with malignant brain tumors [69]. The early and significant depletion of T_Regs_ shortly after antibody administration in patients after surgical resection, suggests that n T_Regs_ are likely effectively depleted by this treatment but determination of the effects of anti-IL-2Rα MAbs on T_Reg_ subsets constitute an important area for future research.

The results of our trial examining the impact of daclizumab and anti-tumor vaccination differ widely from the recent work of Jacobs *et al.* [32] in which the administration of daclizumab successfully depleted T_Regs_ but impaired vaccine-stimulated T-cell function and prevented antibody formation. In our study, robust PEPvIII-specific humoral responses were present in both the saline and daclizumab arms and the presence of class-switching indicates that functional CD4^+^ T-cell help must have been provided. Additionally, our study demonstrates that lower T_Reg_ levels actually correlate with improved anti-PEPvIII antibody responses. Key differences between our study and the work of Jacobs *et al.* include type of vaccination (peptide versus DC), randomization (randomized versus not randomized) as well as administration and dose of daclizumab (1 mg/kg at vaccination versus 0.5 mg/kg 4 or 8 days prior to vaccination). However, we believe the fundamental difference is that our application of daclizumab and anti-tumor vaccination occurs in the context of lymphopenia and it is this setting that permits daclizumab to selectively deplete T_Regs_ while leaving vaccine-stimulated anti-tumor immunity intact.

Although promising, our study does have a number of limitations, one of which is despite being randomized, blinded and placebo-controlled; the number of patients enrolled in this trial is small due to discontinuation of the availability of daclizumab. However, these results have been reproduced in two separate phase I trials we have conducted more recently. In the first trial, patients were vaccinated against C*ytomegalovirus*, pp65, a tumor antigen now known to be specifically expressed in GBM [70]–[73]. While patients treated with vaccine alone have a median progression free survival of only 15.4 months, patients in the second study [33] treated with a combination of vaccination and concomitant daclizumab have a progression-free survival of 27.2 months. While these are also small studies, these results may be significant given that the expected progression-free survival is 6.9–8.2 months in this patient population [74], [75]. While the EGFRvIII-specific vaccine has been previously shown to stimulate predominantly humoral responses, in these other trials we are additionally examining differences in T-cell responses. The results of our cumulative data provide a safe, novel and much needed method for depleting T_Regs_ without impairing activated effector T-cells. Unfortunately, the discontinuation of the availability of daclizumab precluded further study of the effects of this drug on immunologic responses. A chimeric monoclonal antibody targeted CD25, basiliximab, however is currently available and studies evaluating the use of this antibody to selectively deplete T_Regs_ are underway. The utility of repeated administration of anti-IL-2Rα MAbs during recovery from lymphopenia and the effects of dose-escalation of TMZ to achieve greater and sustained lymphopenia constitute potential avenues for exploitation of the use of anti-IL-2Rα MAbs in cancer immunotherapy.

## Materials and Methods

### Patient Selection, Clinical Protocol and Ethics Statement

The protocol for this trial and supporting CONSORT checklist are available as supporting information; see Checklist S1 and Protocol S1. Adults with a first time histopathologic diagnosis of GBM (WHO Grade IV) and a Karnofsky Performance Scale (KPS) score ≥80 were eligible for vaccination if tumor cells expressed EGFRvIII by Immunohistochemistry (IHC), and they had no radiographic evidence of progression after radiation therapy. The trial design and written Informed Consent were approved by the U.S. Food and Drug Administration (under BB-IND-9,944) and the local Institutional Review Board at Duke University (00000947). Prior to the first vaccine the patients were randomized to receive daclizumab or saline. The original study was designed for enrollment of twenty patients but was halted after enrollment of six patients due to discontinuation of the drug daclizumab by the manufacturer (Roche).

### Vaccine Product and Administration

The vaccine consisted of a 13-amino-acid peptide that spans the EGFRvIII mutation (LEEKKGNYVVTDHC) conjugated to keyhole limpet hemocyanin (KLH) and was manufactured by Celldex, CDX-110/rindopepimut. The CDX-110/rindopepimut (500 µg/immunization) was mixed with granulocyte-macrophage colony-stimulating factor (GM-CSF) (150 µg/immunization) within 30 minutes of administration. All vaccines were given intradermally within 10 cm of the inguinal ligament on alternating sides on day 21±2 of each 28 day TMZ cycle.

### Flow cytometric analysis of PBMC

PBMC were potentially stained for the following surface antigens: CD4-FITC (clone RPA-T4; BD, San Diego, CA), CD8 (clone RPA-T8; BD Bioscience, San Diego, CA), CD25-PE (clone MA251 or clone 2A3; BD Bioscience, San Diego, CA), CD127-PerCpP-Cy5.5 (clone hIL-7R-M21; BD Bioscience , San Diego, CA) or goat anti-human-PE (#109-486-127, F(ab′)2 fragment; Jackson Immuno Research, West Grove, PA). Cells were washed extensively and incubated on ice for 30 minutes in fixation/permeabilization buffer (eBioscience, Cat # 00-5123-43, San Diego, CA). For T_Reg_ analysis, after surface staining cells were washed in 1× permeabilization buffer (eBioscience, Cat # 00-8333-56, San Diego, CA), pelleted, and stained with Foxp3-APC (clone PCH101; eBioscience, San Diego, CA). Samples were acquired on BD FACS Calibur (BD, San Diego, CA) and analyzed with FlowJo (TreeStar, Ashland, OR).

### In vitro analysis of PBMC

PBMC from normal donors were incubated for 48 hours in AIM-V media (Gibco, Cat # 0870112, Carlsbad, CA)+2% human AB serum (Valley Biomedical, Cat # HP1022, Winchester, VA) with various levels of daclizumab. After washing, cells were stimulated with CMV pp65 RNA-pulsed DCs (1∶10, DC∶Tcell) in AIM-V+2% human AB serum supplemented with 100 U/ml IL-2 (Proleukin, Prometheus, San Diego, CA) or 10 ng/ml IL-15 (PeproTech, Cat # AF-200-15, Rocky Hill, NJ) for 14 days. Harvested cells were washed and stimulated for 6 hours with SEB (Sigma, Cat # S0281, St Louis, MO) or CMV peptide pool (BD Bioscience, Cat # 551969) in AIM-V+2% human AB serum supplemented with Brefeldin A (BD Bioscience, Cat # 347688 and CD28/CD49d (BD Bioscience, Clone L25/L293). Cells were washed, fixed with FAC Lyse (BD Bioscience, Cat # 349202), permeablized with Perm 2 (BD Bioscience, Cat # 347692), stained with FastImmune CD4/CD69/CD3 (BD Bioscience, Clones SK3/L78/SK7) or CD8/CD69/CD3 (BD Bioscience, Clones SK1/L78/SK7)+IFNγ (BD Bioscience, Clone B27), and analyzed on BD FACS Calibur.

### Antibody Titers

Patient serums were analyzed for humoral response against EGFRvIII antigen (PEPvIII) by a flow cytometry bead assay as previously described [76]. Briefly, PEPvIII was immobilized on magnetic particles (Dynal M280 tosylactivated beads, Cat # 142.03, Invitrogen Corporation, Carlsbad, CA) following manufactures directions. Serum samples were diluted 1∶100 with PBS+0.1% Tween 20 (Sigma, Cat # P7949, St. Louis, MO) and human anti-PEPvIII antibody was captured during 30 minute incubation with beads. After washing away unbound material, captured antibody was detected through the binding of a labeled secondary anti-human polyclonal antibody (Jackson Immuno Research, goat anti-human-PE, F(ab′)2 fragment specific for both IgG and IgM, #109-116-127) in an additional 30 minute incubation step. Beads were again washed to remove unbound goat anti-human-PE before analysis and labeled beads were then analyzed on a flow cytometer to determine their mean fluorescent intensity (MFI). Humanized anti-EGFRvIII (L8A4) was used to generate standard curve and Prism software was used to convert MFI to ng/ml. To ensure specificity, separate serum samples were pre-adsorbed with PEPvIII to block specific antibody from binding to beads. Samples were acquired on BD FACS Calibur and analyzed with FlowJo.

### Statistical Analysis

For each patient, the average percent change in the frequency of CD4^+^Foxp3^+^ regulatory T-cells between baseline (i.e. vaccine 1) and the vaccine 2, 3 and leukapheresis time points was calculated. These follow-up assessments occurring immediately after daclizumab administration were of primary interest as the serum half-life of daclizumab is 20 days [56] and levels will continuously decrease over time. A two-sample t-test was used to compare daclizumab and saline arms with respect to this measure. Similar analyses were conducted for other immunologic measures with a focus on the average percent change between baseline and follow-up time points between baseline, leukapheresis, and vaccine 4. For cortisol, TSH, and ACTH, analyses focused on the average percent change between baseline and vaccine 4, 5, and 6. The spearman rank correlation coefficient was used to assess the association between the frequency regulatory T-cells and anti-PEPvIII humoral response. All statistical analyses were conducted using SAS 9.2 (SAS Institute, Cary, NC). A two-sided significance level of 0.05 was used for statistical tests.

## Supporting Information

Checklist S1 **CONSORT 2010 Checklist of Information.** Checklist for the Zenapax® Activated Peptide Immunotherapy (ZAP IT) Clinical Trial.
(DOC)

Protocol S1 **Clinical Protocol.** Clinical Protocol for the Zenapax® Activated Peptide Immunotherapy (ZAP IT) Clinical Trial.
(PDF)

## References

[1] Zou W (2006). Regulatory T cells, tumour immunity and immunotherapy.. Nat Rev Immunol.

[2] Thornton AM, Shevach EM (1998). CD4+CD25+ immunoregulatory T cells suppress polyclonal T cell activation in vitro by inhibiting interleukin 2 production.. J Exp Med.

[3] Jonuleit H, Schmitt E, Stassen M, Tuettenberg A, Knop J (2001). Identification and functional characterization of human CD4(+)CD25(+) T cells with regulatory properties isolated from peripheral blood.. J Exp Med.

[4] Dieckmann D, Plottner H, Berchtold S, Berger T, Schuler G (2001). Ex vivo isolation and characterization of CD4(+)CD25(+) T cells with regulatory properties from human blood.. J Exp Med.

[5] Fontenot JD, Gavin MA, Rudensky AY (2003). Foxp3 programs the development and function of CD4+CD25+ regulatory T cells.. Nat Immunol.

[6] Khattri R, Cox T, Yasayko SA, Ramsdell F (2003). An essential role for Scurfin in CD4+CD25+ T regulatory cells.. Nat Immunol.

[7] Green DR, Webb DR (1993). Saying the ‘S’ word in public.. Immunol Today.

[8] Sakaguchi S, Sakaguchi N, Asano M, Itoh M, Toda M (1995). Immunologic self-tolerance maintained by activated T cells expressing IL-2 receptor alpha-chains (CD25). Breakdown of a single mechanism of self-tolerance causes various autoimmune diseases.. J Immunol.

[9] Asano M, Toda M, Sakaguchi N, Sakaguchi S (1996). Autoimmune disease as a consequence of developmental abnormality of a T cell subpopulation.. J Exp Med.

[10] Salomon B, Lenschow DJ, Rhee L, Ashourian N, Singh B (2000). B7/CD28 costimulation is essential for the homeostasis of the CD4+CD25+ immunoregulatory T cells that control autoimmune diabetes.. Immunity.

[11] Stephens LA, Mason D (2000). CD25 is a marker for CD4+ thymocytes that prevent autoimmune diabetes in rats, but peripheral T cells with this function are found in both CD25+ and CD25− subpopulations.. J Immunol.

[12] Taguchi O, Nishizuka Y (1987). Self tolerance and localized autoimmunity. Mouse models of autoimmune disease that suggest tissue-specific suppressor T cells are involved in self tolerance.. J Exp Med.

[13] Taguchi O, Kontani K, Ikeda H, Kezuka T, Takeuchi M (1994). Tissue-specific suppressor T cells involved in self-tolerance are activated extrathymically by self-antigens.. Immunology.

[14] Seddon B, Mason D (1999). Regulatory T cells in the control of autoimmunity: the essential role of transforming growth factor beta and interleukin 4 in the prevention of autoimmune thyroiditis in rats by peripheral CD4(+)CD45RC- cells and CD4(+)CD8(−) thymocytes.. J Exp Med.

[15] Seddon B, Mason D (1999). Peripheral autoantigen induces regulatory T cells that prevent autoimmunity.. J Exp Med.

[16] Bagavant H, Thompson C, Ohno K, Setiady Y, Tung KSK (2002). Differential effect of neonatal thymectomy on systemic and organ-specific autoimmune disease.. Int Immunol.

[17] Somasundaram R, Jacob L, Swoboda R, Caputo L, Song H (2002). Inhibition of cytolytic T lymphocyte proliferation by autologous CD4+/CD25+ regulatory T cells in a colorectal carcinoma patient is mediated by transforming growth factor-beta.. Cancer Res.

[18] Curiel TJ, Coukos G, Zou L, Alvarez X, Cheng P (2004). Specific recruitment of regulatory T cells in ovarian carcinoma fosters immune privilege and predicts reduced survival.. Nat Med.

[19] Liyanage UK, Moore TT, Joo HG, Tanaka Y, Herrmann V (2002). Prevalence of regulatory T cells is increased in peripheral blood and tumor microenvironment of patients with pancreas or breast adenocarcinoma.. J Immunol.

[20] Wolf AM, Wolf D, Steurer M, Gastl G, Gunsilius E (2003). Increase of regulatory T cells in the peripheral blood of cancer patients.. Clin Cancer Res.

[21] Ichihara F, Kono K, Takahashi A, Kawaida H, Sugai H (2003). Increased populations of regulatory T cells in peripheral blood and tumor-infiltrating lymphocytes in patients with gastric and esophageal cancers.. Clin Cancer Res.

[22] Woo EY, Chu CS, Goletz TJ, Schlienger K, Yeh H (2001). Regulatory CD4(+)CD25(+) T cells in tumors from patients with early-stage non-small cell lung cancer and late-stage ovarian cancer.. Cancer Res.

[23] Fecci PE, Mitchell DA, Whitesides JF, Xie W, Friedman AH (2006). Increased regulatory T-cell fraction amidst a diminished CD4 compartment explains cellular immune defects in patients with malignant glioma.. Cancer Res.

[24] Morse MA, Hobeika AC, Osada T, Serra D, Niedzwiecki D (2008). Depletion of human regulatory T cells specifically enhances antigen-specific immune responses to cancer vaccines.. Blood.

[25] Dannull J, Su Z, Rizzieri D, Yang BK, Coleman D (2005). Enhancement of vaccine-mediated antitumor immunity in cancer patients after depletion of regulatory T cells.. J Clin Invest.

[26] Attia P, Maker AV, Haworth LR, Rogers-Freezer L, Rosenberg SA (2005). Inability of a fusion protein of IL-2 and diphtheria toxin (Denileukin Diftitox, DAB389IL-2, ONTAK) to eliminate regulatory T lymphocytes in patients with melanoma.. J Immunother.

[27] Powell DJ, Felipe-Silva A, Merino MJ, Ahmadzadeh M, Allen T (2007). Administration of a CD25-directed immunotoxin, LMB-2, to patients with metastatic melanoma induces a selective partial reduction in regulatory T cells in vivo.. J Immunol.

[28] Curtin JF, Candolfi M, Fakhouri TM, Liu C, Alden A (2008). Treg depletion inhibits efficacy of cancer immunotherapy: implications for clinical trials.. PLoS One.

[29] Goebel J, Stevens E, Forrest K, Roszman TL (2000). Daclizumab (Zenapax) inhibits early interleukin-2 receptor signal transduction events.. Transpl Immunol.

[30] Fecci PE, Sweeney AE, Grossi PM, Nair SK, Learn CA (2006). Systemic anti-CD25 monoclonal antibody administration safely enhances immunity in murine glioma without eliminating regulatory T cells.. Clin Cancer Res.

[31] Kohm AP, McMahon JS, Podojil JR, Begolka WS, DeGutes M (2006). Cutting Edge: Anti-CD25 monoclonal antibody injection results in the functional inactivation, not depletion, of CD4+CD25+ T regulatory cells.. J Immunol.

[32] Jacobs JF, Punt CJ, Lesterhuis WJ, Sutmuller RP, Brouwer HM (2010). Dendritic Cell Vaccination in Combination with Anti-CD25 Monoclonal Antibody Treatment: A Phase I/II Study in Metastatic Melanoma Patients.. Clin Cancer Res.

[33] Mitchell DA, Cui X, Schmittling RJ, Sanchez-Perez L, Snyder DJ (2011). Monoclonal antibody blockade of IL-2R{alpha} during lymphopenia selectively depletes regulatory T cells in mice and humans.. Blood.

[34] Humphrey PA, Wong AJ, Vogelstein B, Zalutsky MR, Fuller GN (1990). Anti-synthetic peptide antibody reacting at the fusion junction of deletion-mutant epidermal growth factor receptors in human glioblastoma.. Proc Natl Acad Sci U S A.

[35] Wikstrand CJ, Hale LP, Batra SK, Hill ML, Humphrey PA (1995). Monoclonal antibodies against EGFRvIII are tumor specific and react with breast and lung carcinomas and malignant gliomas.. Cancer Res.

[36] Purev E, Cai D, Miller E, Swoboda R, Mayer T (2004). Immune responses of breast cancer patients to mutated epidermal growth factor receptor (EGF-RvIII, Delta EGF-R, and de2-7 EGF-R).. J Immunol.

[37] Sok JC, Coppelli FM, Thomas SM, Lango MN, Xi S (2006). Mutant epidermal growth factor receptor (EGFRvIII) contributes to head and neck cancer growth and resistance to EGFR targeting.. Clin Cancer Res.

[38] Sampson JH, Aldape KD, Archer GE, Coan A, Desjardins A (2011). Greater chemotherapy-induced lymphopenia enhances tumor-specific immune responses that eliminate EGFRvIII-expressing tumor cells in patients with glioblastoma.. Neuro Oncol.

[39] Sampson JH, Heimberger AB, Archer GE, Aldape KD, Friedman AH (2010). Immunologic escape after prolonged progression-free survival with epidermal growth factor receptor variant III peptide vaccination in patients with newly diagnosed glioblastoma.. J Clin Oncol.

[40] Sampson JH, Archer GE, Mitchell DA, Heimberger AB, Herndon JE, 2nd (2009). An epidermal growth factor receptor variant III-targeted vaccine is safe and immunogenic in patients with glioblastoma multiforme.. Mol Cancer Ther.

[41] Binder M, Vogtle FN, Michelfelder S, Muller F, Illerhaus G (2007). Identification of their epitope reveals the structural basis for the mechanism of action of the immunosuppressive antibodies basiliximab and daclizumab.. Cancer Res.

[42] Bielekova B, Catalfamo M, Reichert-Scrivner S, Packer A, Cerna M (2006). Regulatory CD56(bright) natural killer cells mediate immunomodulatory effects of IL-2Ralpha-targeted therapy (daclizumab) in multiple sclerosis.. Proc Natl Acad Sci U S A.

[43] Gattinoni L, Finkelstein SE, Klebanoff CA, Antony PA, Palmer DC (2005). Removal of homeostatic cytokine sinks by lymphodepletion enhances the efficacy of adoptively transferred tumor-specific CD8+ T cells.. J Exp Med.

[44] Phan GQ, Yang JC, Sherry RM, Hwu P, Topalian SL (2003). Cancer regression and autoimmunity induced by cytotoxic T lymphocyte-associated antigen 4 blockade in patients with metastatic melanoma.. Proc Natl Acad Sci U S A.

[45] Attia P, Phan GQ, Maker AV, Robinson MR, Quezado MM (2005). Autoimmunity correlates with tumor regression in patients with metastatic melanoma treated with anti-cytotoxic T-lymphocyte antigen-4.. J Clin Oncol.

[46] Jaber SH, Cowen EW, Haworth LR, Booher SL, Berman DM (2006). Skin reactions in a subset of patients with stage IV melanoma treated with anti-cytotoxic T-lymphocyte antigen 4 monoclonal antibody as a single agent.. Arch Dermatol.

[47] Blansfield JA, Beck KE, Tran K, Yang JC, Hughes MS (2005). Cytotoxic T-lymphocyte-associated antigen-4 blockage can induce autoimmune hypophysitis in patients with metastatic melanoma and renal cancer.. J Immunother.

[48] Setoguchi R, Hori S, Takahashi T, Sakaguchi S (2005). Homeostatic maintenance of natural Foxp3(+) CD25(+) CD4(+) regulatory T cells by interleukin (IL)-2 and induction of autoimmune disease by IL-2 neutralization.. J Exp Med.

[49] Neujahr DC, Chen C, Huang X, Markmann JF, Cobbold S (2006). Accelerated memory cell homeostasis during T cell depletion and approaches to overcome it.. J Immunol.

[50] Quezada SA, Peggs KS, Simpson TR, Shen Y, Littman DR (2008). Limited tumor infiltration by activated T effector cells restricts the therapeutic activity of regulatory T cell depletion against established melanoma.. J Exp Med.

[51] Wrzesinski C, Paulos CM, Gattinoni L, Palmer DC, Kaiser A (2007). Hematopoietic stem cells promote the expansion and function of adoptively transferred antitumor CD8 T cells.. J Clin Invest.

[52] Sato E, Olson SH, Ahn J, Bundy B, Nishikawa H (2005). Intraepithelial CD8+ tumor-infiltrating lymphocytes and a high CD8+/regulatory T cell ratio are associated with favorable prognosis in ovarian cancer.. Proc Natl Acad Sci U S A.

[53] Nair S, Boczkowski D, Fassnacht M, Pisetsky D, Gilboa E (2007). Vaccination against the forkhead family transcription factor Foxp3 enhances tumor immunity.. Cancer Res.

[54] Koyama K, Kagamu H, Miura S, Hiura T, Miyabayashi T (2008). Reciprocal CD4+ T-cell balance of effector CD62Llow CD4+ and CD62LhighCD25+ CD4+ regulatory T cells in small cell lung cancer reflects disease stage.. Clin Cancer Res.

[55] Martin JF, Perry JS, Jakhete NR, Wang X, Bielekova B An IL-2 paradox: blocking CD25 on T cells induces IL-2-driven activation of CD56(bright) NK cells.. J Immunol.

[56] Vincenti F, Nashan B, Light S (1998). Daclizumab: outcome of phase III trials and mechanism of action. Double Therapy and the Triple Therapy Study Groups.. Transplant Proc.

[57] Van Assche G, Sandborn WJ, Feagan BG, Salzberg BA, Silvers D (2006). Daclizumab, a humanised monoclonal antibody to the interleukin 2 receptor (CD25), for the treatment of moderately to severely active ulcerative colitis: a randomised, double blind, placebo controlled, dose ranging trial.. Gut.

[58] Boissonnas A, Scholer-Dahirel A, Simon-Blancal V, Pace L, Valet F (2010). Foxp3+ T cells induce perforin-dependent dendritic cell death in tumor-draining lymph nodes.. Immunity.

[59] Powell DJ, Attia P, Ghetie V, Schindler J, Vitetta ES (2008). Partial reduction of human FOXP3+ CD4 T cells in vivo after CD25-directed recombinant immunotoxin administration.. J Immunother.

[60] Attia P, Powell DJ, Maker AV, Kreitman RJ, Pastan I (2006). Selective elimination of human regulatory T lymphocytes in vitro with the recombinant immunotoxin LMB-2.. J Immunother.

[61] Maloy KJ, Powrie F (2005). Fueling regulation: IL-2 keeps CD4+ Treg cells fit.[comment].. Nat Immunol.

[62] D'Cruz LM, Klein L (2005). Development and function of agonist-induced CD25+Foxp3+ regulatory T cells in the absence of interleukin 2 signaling.. Nat Immunol.

[63] Fontenot JD, Rasmussen JP, Gavin MA, Rudensky AY (2005). A function for interleukin 2 in Foxp3-expressing regulatory T cells.. Nat Immunol.

[64] Malek TR, Bayer AL (2004). Tolerance, not immunity, crucially depends on IL-2.. Nat Rev Immunol.

[65] Onizuka S, Tawara I, Shimizu J, Sakaguchi S, Fujita T (1999). Tumor rejection by in vivo administration of anti-CD25 (interleukin-2 receptor alpha) monoclonal antibody.. Cancer Res.

[66] Antony PA, Piccirillo CA, Akpinarli A, Finkelstein SE, Speiss PJ (2005). CD8+ T cell immunity against a tumor/self-antigen is augmented by CD4+ T helper cells and hindered by naturally occurring T regulatory cells.. J Immunol.

[67] Turk MJ, Guevara-Patino JA, Rizzuto GA, Engelhorn ME, Sakaguchi S (2004). Concomitant tumor immunity to a poorly immunogenic melanoma is prevented by regulatory T cells.. J Exp Med.

[68] Williams MA, Tyznik AJ, Bevan MJ (2006). Interleukin-2 signals during priming are required for secondary expansion of CD8+ memory T cells.. Nature.

[69] Wainwright DA, Sengupta S, Han Y, Lesniak MS (2011). Thymus-derived rather than tumor-induced regulatory T cells predominate in brain tumors.. Neuro Oncol.

[70] Mitchell DA, Xie W, Schmittling R, Learn C, Friedman A (2008). Sensitive detection of human cytomegalovirus in tumors and peripheral blood of patients diagnosed with glioblastoma.. Neuro Oncol.

[71] Cobbs CS, Harkins L, Samanta M, Gillespie GY, Bharara S (2002). Human cytomegalovirus infection and expression in human malignant glioma.. Cancer Res.

[72] Prins RM, Cloughesy TF, Liau LM (2008). Cytomegalovirus immunity after vaccination with autologous glioblastoma lysate.. N Engl J Med.

[73] Scheurer ME, Bondy ML, Aldape KD, Albrecht T, El-Zein R (2008). Detection of human cytomegalovirus in different histological types of gliomas.. Acta Neuropathol.

[74] Stupp R, Mason WP, van den Bent MJ, Weller M, Fisher B (2005). Radiotherapy plus concomitant and adjuvant temozolomide for glioblastoma.. N Engl J Med.

[75] Liau LM, Prins RM, Kiertscher SM, Odesa SK, Kremen TJ (2005). Dendritic cell vaccination in glioblastoma patients induces systemic and intracranial T-cell responses modulated by the local central nervous system tumor microenvironment.. Clin Cancer Res.

[76] Schmittling RJ, Archer GE, Mitchell DA, Heimberger A, Pegram C (2008). Detection of humoral response in patients with glioblastoma receiving EGFRvIII-KLH vaccines.. J Immunol Methods.

