# Supplementary material for: A Pilot Study of IL-2Rα Blockade during Lymphopenia Depletes Regulatory T-cells and Correlates with Enhanced Immunity in Patients with Glioblastoma
Source: PLoS One. 2012 Feb 27;7(2):e31046. doi: 10.1371/journal.pone.0031046 (PMC3288003; doi:10.1371/journal.pone.0031046)
Supplement: Protocol S1 — Clinical Protocol. Clinical Protocol for the Zenapax® Activated Peptide Immunotherapy (ZAP IT) Clinical Trial. (PDF) [file pone.0031046.s002.pdf]

ZAP IT:
Zenapax®-Activated Peptide ImmunoTherapy

Investigational Review Board Number 9047
Food and Drug Administration Investigational New Drug Permit 9,944

Duke University Medical Center
Gary E. Archer, Ph.D.
Darell D. Bigner, M.D., Ph.D.
R. Edward Coleman, M.D.
Annick Desjardins, M.D.
Allan H. Friedman, M.D.
Henry S. Friedman, M.D.
James E. Herndon, II, Ph.D.
John Kirkpatrick, M.D., Ph.D.
Joanne Kurtzberg, M.D.
Denise Lally-Goss, R.N., M.S.N.
Sharon McGehee
Roger E. McLendon, M.D.
Duane A. Mitchell, M.D., Ph.D. (Principal Investigator)
Beth Perry, R.N.
James M. Provenzale, M.D.
Jennifer A. Quinn, M.D.
Renee Hinsley Raynor, Ph.D
David Reardon, M.D.
Jeremy Rich, M.D.
John H. Sampson, M.D., Ph.D. (Sponsor)
Sith Sathornsumetee, M.D.
Robert Schmittling, B.S.
Greg Sempowski, Ph.D.
James J. Vredenburgh, M.D.
Kent Weinhold, Ph.D.

STUDY SYNOPSIS

**Title**	ZAP IT: Zenapax®-Activated Peptide ImmunoTherapy
**Study Drug:**	PEPvIII-KLH (now known as CDX-110): a tumor-specific peptide spanning the variant III mutation in the epidermal growth factor receptor, EGFRvIII[1] conjugated to keyhole limpet hemocyanin (KLH[2])(PEPvIII) with granulocyte macrophage-colony stimulating factor (GM-CSF[3]); Peripheral Blood Lymphocytes (PBLs[4]); and daclizumab (Zenapax), a humanized IgG1 monoclonal antibody which binds to the alpha subunit (CD25 or TAC subunit) of the human interleukin-2 (IL[5]-2) receptor with high affinity and blocks IL-2 binding, and signaling.
**Rationale:**	Malignant primary brain tumors are the most common cause of death among children, and account for more deaths in adults than melanoma. This is principally because conventional therapy is severely constrained by the need to eradicate tumor cells that are hidden behind a restrictive blood-brain barrier or that have invaded eloquent brain tissue. As a result, surgery and radiation must be curtailed to avoid incapacitating collateral damage, and chemotherapy becomes toxic to rapidly dividing extracerebral normal tissues before eliminating all intracerebral tumor cells. The immune system, however, has the capacity to eliminate altered neoplastic cells with incredible specificity. A consistent in-frame deletion in the extra-cellular domain of the EGFR produces a constitutively active tyrosine kinase that enhances neoplastic cell growth and migration and confers radiation and chemotherapeutic resistance to tumor cells in patients with glioblastoma multiforme (GBM[6]) and a broad array of other common cancers. It also results in a truly tumor-specific target amenable to immunotherapeutic attack. Our multi-institutional Phase II study demonstrates that vaccinations with an EGFRvIII-specific peptide induce T- and B-cell immunity, produce complete radiographic responses in all patients with residual tumor, and universally eliminate EGFRvIII-expressing cells. Recurrent tumors, however, continue to express wild-type EGFR suggesting that the immune response is specific, but productive intra-molecular cross-priming does not occur. We believe that cross-priming may be hindered by the presence of regulatory T-cells (T_{Regs}[7]). We have recently shown that T_{Regs} are disproportionately represented within the peripheral blood and tumors of patients with GBM and serve to induce a state of profound, but reversible, immunosuppression. T_{Regs} are characterized by constitutive expression of the high affinity interleukin IL-2 receptor (IL-2Rα) (CD25) and are absolutely and uniquely dependent on IL-2Rα signaling for their function and survival. Using our spontaneous murine glioma model, we have already demonstrated that pre-treatment with an antibody that blocks IL-2Rα signaling and functionally inactivates T_{Regs} without inducing autoimmune toxicity. Established immune responses may also depend on IL-2 signaling of effector T-cells or a novel subset of $CD3^{-}CD56^{bright}$ natural killer (NK[8]) cells, however, such that blocking IL-2Rα signaling during ongoing immune responses may impair effector T-cell responses as well. After periods of lymphopenia, however, physiologic surges in IL-7 and IL-15 drive homeostatic reconstitution of the effector T-cell compartment and may bypass the need and effects of IL-2 signaling in these cells. We have recently shown that temozolomide (TMZ[9]), an alkylating agent that produces a survival benefit in patients with GBM, induces a profound, but transient lymphopenic environment in humans and mice. Our preliminary studies have shown that unarmed CD25-specific antibodies given *in vivo* to mice during recovery from TMZ-induced lymphopenia and in *in vitro* human studies are capable of functionally inactivating T_{Regs} while dramatically enhancing vaccine-induced immune responses. Although others have used recombinant toxins conjugated to IL-2 to deplete T_{Regs}, results have been variable. This may be due to counter productive direct IL-2 signaling of T_{Regs}, depletion of effector memory cells through constitutively expressed lower affinity IL-2Rs, rapid clearance of the foreign toxin, or depletion of activated T-cells that also transiently express CD25. Conversely, *daclizumab*, an existing humanized unarmed CD25-specific antibody, functions identically to the antibody used for T_{Reg} inactivation studies in mice by blocking IL-2 signaling which is critical only to T_{Reg} function and survival during lymphopenic recovery. In this protocol, we will determine if daclizumab inhibits the functional recovery of T_{Regs} after therapeutic TMZ-induced lymphopenia in the context of vaccinating adult patients with newly-diagnosed high grade gliomas using a tumor-specific, EGFRvIII-targeted peptide vaccine.
**Primary Objective:**	1. To determine if daclizumab inhibits the functional recovery of T_{Regs} after therapeutic TMZ-induced lymphopenia in the context of vaccinating adult patients with newly-diagnosed high grade gliomas using CDX-110.
**Secondary Objectives:**	1. To evaluate the safety of daclizumab in the context of vaccinating adult patients with newly-diagnosed high grade gliomas using CDX-110 during recovery from therapeutic TMZ-induced lymphopenia.<br>2. To determine if daclizumab enhances the magnitude or character of CDX-110-induced cellular or humoral immune responses, inhibits or enhances activation-induced cell death, or induces immunologic or clinical evidence of autoimmunity.<br>3. To determine if daclizumab alters the phenotype (CD56-expression), cytokine secretion profile, or cytotoxicity of CD3-CD56+ NK cells.<br>4. To determine if daclizumab in addition to vaccination extends progression free survival compared to historical cohorts.<br>5. To characterize immunologic cell infiltrate in recurrent tumors and seek evidence of antigen escape outgrowth.
**Inclusion Criteria:**	• Age ≥18 years of age.<br>• Surgically resected, histologically confirmed $EGFRvIII^{+}$ High Grade Glioma (WHO Grade III or IV).<br>• Karnofsky Performance Status (KPS[10]) of ≥ 80% and a Curran Group status of I-IV.

[1] EGFRvIII, epidermal growth factor receptor variant III
[2] KLH, keyhole limpet hemocyanin
[3] GM-CSF, granulocyte macrophage-colony stimulating factor
[4] PBLs, peripheral blood lymphocytes
[5] IL, interleukin
[6] Gbm, glioblastoma multiforme
[7] TRegs, regulatory T-cells
[8] NK, natural killer
[9] TMZ, temozolomide
[10] KPS, Karnofsky performance status

**Exclusion Criteria:**	• Radiographic or cytologic evidence of leptomeningeal or multicentric disease at any time prior to vaccination.<br>• Prior conventional antitumor therapy other than steroids, RT, or TMZ.<br>• Pregnant or need to breast feed during the study period (Negative β-HCG test required).<br>• Requirement for continuous corticosteroids above physiologic levels at time of first vaccination.<br>• Active infection requiring treatment or an unexplained febrile (> 101.5^{o} F) illness.<br>• Known immunosuppressive disease or human immunodeficiency virus infection.<br>• Patients with unstable or severe intercurrent medical conditions such as severe heart or lung disease.<br>• Allergic or unable to tolerate TMZ for reasons other than lymphopenia.<br>• Patients with previous inguinal lymph node dissection.<br>• Prior treatment with daclizumab for any reason.<br>• Prior allogeneic solid organ transplant.
**Study Design:**	Twenty patients with newly-diagnosed EGFRvIII+ high grade glioma (WHO III or IV) will undergo leukapheresis for immunologic monitoring. Only 1 dose level of daclizumab (1 mg/kg) will be assessed. The decision to dose escalate in subsequent trials will be dependent on analysis of the safety and immunologic responses obtained in this trial. Patients will be followed until death. This study will be halted if any 2 patients experience a drug-related Grade IV or irreversible Grade III toxicity. After initial leukapheresis, all patients will then receive RT and concurrent TMZ at a standard targeted dose of 75 mg/m^2/d. Patients with progressive disease during radiation, dependent on steroid supplements above physiologic levels at time of vaccination, unable to tolerate TMZ or whose PBLs fail to meet release criteria will be replaced, but no more than 40 patients will be enrolled overall. Remaining patients will then receive the initial cycle of TMZ at a targeted dose of 100mg/m^2/d for 21 days 3 $\pm$ 1 weeks after completing RT and will be independently randomized to receive daclizumab versus saline simultaneous with vaccine #1. An additional cohort of 6 patients (cohort #2) will receive serial administrations of daclizumab at vaccine # 1, vaccine # 3, and during the recovery from each TMZ cycle for the first 3 cycles at the same dose. Vaccine #1 will be given on day 21±2 days after the start of the 100mg/m^2/d dose of TMZ. While standard dose 21-day TMZ treatment at a target dose of 100 mg/m^2/day is recommended, TMZ regimen may be adjusted at the discretion of the treating neuro-oncologist. Vaccine will always be given on day 21 ± 2 of each 28 day TMZ cycle. Vaccines will be given intradermally and divided equally to both inguinal regions. The calculated dosage of daclizumab will be administered in a 50 mL of sterile 0.9% sodium chloride solution via a peripheral or central vein over a 15 minute period. Vaccine #2 and #3 will occur at 2 week intervals. Patients will then be treated monthly with TMZ cycles for a total of 6 cycles after RT. TMZ will be given on days 1-5 of a 28 day cycle. Subsequent vaccines will be given on day 21$\pm$2 of each of the five remaining TMZ cycles. Cohort # 2 will receive sugsequent vaccinations along with daclizumab (1 mg/Kg) on day 21 ± 2 for first 3 cycles. Vaccines will then continue monthly until progression. An additional leukapheresis will be required for any patients who have a positive DTH skin test for PEPvIII for immunologic monitoring. Patients will be imaged after vaccine #4 and then bimonthly without receiving any other prescribed antitumor therapy until progression. Blood work for immunologic monitoring will be drawn before vaccine #1 and then each vaccine after vaccine #3. Blood work for sub-clinical autoimmunity will be drawn before vaccine #1 and every 4 months after vaccine #3. As part of standard care for these patients, upon tumor progression, participants may undergo stereotactic biopsy or resection. As this is not a research procedure consent will be obtained separately. However, if tissue is obtained, it will be used to confirm tumor progression histologically and to assess immunologic cell infiltration and EGFRvIII antigen escape at the tumor site.

TABLE of CONTENTS

1 ABSTRACT AND STUDY SCHEMA 6
1.1 Abstract 6
1.2 Study Schema 8
2 HYPOTHESES AND OBJECTIVES 9
2.1 Hypotheses 9
2.2 Objectives 9
3 BACKGROUND AND RATIONALE 11
3.1 Disease and Current Therapy 11
3.2 Vaccine Therapy for Cancer 11
3.3 EGFRvIII 13
3.4 CDX-110 14
3.5 Cross-Priming 19
3.6 Autoimmune Encephalomyelitis 19
3.7 T Cell Homeostatic Proliferation 21
3.8 Treatment-Induced Lymphopenia from Temozolomide 23
3.9 Immunosuppression in Patients with GBM and CD25+ T_{Regs} 24
3.10 Inhibition of T_{Reg} Function with Anti-CD25-specific Monoclonal Antibodies 27
3.11 IL-2α-specific Antibodies: PC61 and Daclizumab 30
3.12 TMZ-induced Lymphopenia and IL-2Rα Blockade Enhance Immunity 31
3.13 Daclizumab Eliminates T_{Reg} Function in Monkeys 35
3.14 Daclizumab Eliminates Human T_{Reg} and Enhances CD4+ T-cell Response 35
3.15 Rationale for Current Study 36
4 STUDY POPULATION 40
4.1 Inclusion Criteria 40
4.2 Exclusion Criteria 40
5 INVESTIGATIONAL PLAN 42
5.1 Preparation and Administration of Therapeutic Agents 42
5.2 Treatment Procedures 46
5.3 Safety, Toxicity, and Adverse Events 48
5.4 Adverse Event Reporting and Documentation 49
6 DATA AND SPECIMEN COLLECTION 51
6.1 Pretreatment Evaluation 51
6.2 Data and Specimens to be Accessioned 52
7 CLINICAL RESPONSE EVALUATIONS AND STATISTICS 54
7.1 Radiographic Response and Progression Criteria for Patients with Residual Disease 54
7.2 Special Circumstances 55
7.3 Time to Progression (TTP) 57
7.4 Survival 57
8 IMMUNOLOGIC MONITORING AND STATISTICAL ANALYSIS 58
8.2 T_{Reg} Functional Assays 59
8.3 T_{Reg} Counts 60
8.4 T-cell Cytokine Production 60
8.5 EGFRvIII $CD4^+$ and $CD8^+$ Cytokine Fluorescent Cytometry 61
8.6 Activation-induced Cell Death and *In Vivo* Proliferation 62

8.7 Delayed-type Hypersensitivity ... 62
8.8 PBMC Proliferation and Cytokine Secretion ELISA ... 63
8.9 Serology ... 63
8.10 NK Cell Assays ... 64
8.11 Sub-clinical Autoimmunity ... 64
8.12 Whole Blood for Quantitative PCR for EGFRvIII ... 65
9 SAFETY MONITORING ... 67
9.1 Potential Benefits ... 67
9.2 Potential Risks ... 67
9.3 Data and Safety Monitoring Plan ... 70
10 STATISTICAL CONSIDERATIONS ... 71
10.1 Recruitment ... 71
10.2 Safety Component ... 74
10.3 Time to Progression and Survival ... 74
11 STUDY FLOW SHEET ... 77
12 REFERENCES ... 78

1 ABSTRACT AND STUDY SCHEMA

1.1 Abstract

Malignant primary brain tumors are the most common cause of death among children, and account for more deaths in adults than melanoma(Kotliarov, Steed et al. 2006). This is principally because conventional therapy is severely constrained by the need to eradicate tumor cells that are hidden behind a restrictive blood-brain barrier or that have invaded eloquent brain tissue. As a result, surgery and radiation must be curtailed to avoid incapacitating collateral damage(Imperato, Paleologos et al. 1990), and chemotherapy becomes toxic to rapidly dividing extracerebral normal tissues before eliminating all intracerebral tumor cells. The immune system, however, has the capacity to eliminate altered neoplastic cells with incredible specificity.

A consistent in-frame deletion in the extra-cellular domain of the EGFR produces a constitutively active tyrosine kinase(Batra, Castelino-Prabhu et al. 1995; Chu, Everiss et al. 1997) that enhances neoplastic cell growth and migration(Boockvar, Kapitonov et al. 2003; Pedersen, Tkach et al. 2004) and confers radiation(Lammering, Hewit et al. 2003; Lammering, Hewit et al. 2004; Lammering, Valerie et al. 2004; Li, Yuan et al. 2004) and chemotherapeutic(Montgomery, Guzman et al. 2000; Nagane, Narita et al. 2001) resistance to tumor cells in patients with GBM and a broad array of other common cancers(Garcia de Palazzo, Adams et al. 1993; Moscatello, Holgado-Madruga et al. 1995; Wikstrand, Hale et al. 1995; Olapade-Olaopa, Moscatello et al. 2000; Ge, Gong et al. 2002; Luo, Gong et al. 2003; Okamoto, Kenyon et al. 2003; Aldape, Ballman et al. 2004; Cunningham, Essapen et al. 2005; Heimberger, Hlatky et al. 2005). It also results in a truly tumor-specific target amenable to immunotherapeutic attack. Our multi-institutional Phase II study demonstrates that vaccinations with an EGFRvIII-specific peptide induce T- and B-cell immunity, produce complete radiographic responses in all patients with residual tumor, and universally eliminate EGFRvIII-expressing cells(Heimberger, Hussain et al. 2006; Heimberger, Hussain et al. 2006). Recurrent tumors, however, continue to express wild-type EGFR suggesting that the immune response is specific, but productive intra-molecular cross-priming does not occur. We believe that cross-priming may be hindered by the presence of T_{Regs}.

We have recently shown that T_{Regs} are disproportionately represented within the peripheral blood and tumors of patients with GBM and serve to induce a state of profound, but reversible, immunosuppression(Fecci, Mitchell et al. 2006). T_{Regs} are characterized by constitutive expression of the high affinity IL-2 receptor (IL-2Rα)(CD25) and are absolutely and uniquely dependent on IL-2Rα signaling for their function and survival(Malek 2003; Malek and Bayer 2004; D'Cruz and Klein 2005; Fontenot, Rasmussen et al. 2005; Maloy and Powrie 2005; Fecci, Sweeney et al. 2006). Using our spontaneous murine glioma model, we have already demonstrated that pre-treatment with an antibody that blocks IL-2Rα signaling and functionally inactivates T_{Regs} without inducing autoimmune toxicity(Fecci, Ochiai et al. 2006; Fecci, Sweeney et al. 2006). Established immune responses may also depend on IL-2 signaling of effector T-cells or a novel subset of $CD3^{-}CD56^{bright}$ NK cells(Bielekova, Richert et al. 2004; Li, Lim et al. 2005; Bielekova, Catalfamo et al. 2006), however, such that blocking IL-2Rα signaling during ongoing immune responses may impair effector T-cell responses as well(Williams, Tyznik et al. 2006). After periods of lymphopenia, however, physiologic surges in IL-7(Seddon, Tomlinson et al. 2003; Seddon and Zamoyska 2003) and IL-15(Nishimura, Tagaya et al. 2001) drive homeostatic reconstitution of the effector T-cell

compartment and may bypass the need and effects of IL-2 signaling in these cells(Willerford, Chen et al. 1995; Baan, Boelaars-van Haperen et al. 2001).

We have recently shown that TMZ, an alkylating agent that produces a survival benefit in patients with GBM(Stupp, Mason et al. 2005), induces a profound, but transient lymphopenic environment in humans and mice. Our preliminary studies have shown that unarmed CD25-specific antibodies given *in vivo* to mice during recovery from TMZ-induced lymphopenia and in *in vitro* human studies are capable of functionally inactivating T_{Regs} while dramatically enhancing vaccine-induced immune responses. Although others have used recombinant toxins conjugated to IL-2 to deplete T_{Regs}, results have been variable. This may be due to counter productive direct IL-2 signaling of T_{Regs}, depletion of effector memory cells through constitutively expressed lower affinity IL-2Rs, rapid clearance of the foreign toxin, or depletion of activated T-cells that also transiently express CD25. Conversely, *daclizumab*, an existing humanized unarmed CD25-specific antibody, functions identically to the antibody used for T_{Reg} inactivation studies in mice by blocking IL-2 signaling which is critical only to T_{Reg} function and survival during lymphopenic recovery.

We hypothesize that daclizumab therapy during the recovery from therapeutic TMZ-induced lymphopenia in patients with newly-diagnosed high grade glioma will inhibit the functional recovery of T_{Regs}, enhance vaccine-induced immune responses, and promote cross-priming without the induction of deleterious autoimmunity.

In this protocol, we will determine if daclizumab inhibits the functional recovery of T_{Regs} after therapeutic TMZ-induced lymphopenia in the context of vaccinating adult patients with newly-diagnosed high grade gliomas using a tumor-specific, EGFRvIII-targeted peptide vaccine.

1.2 Study Schema

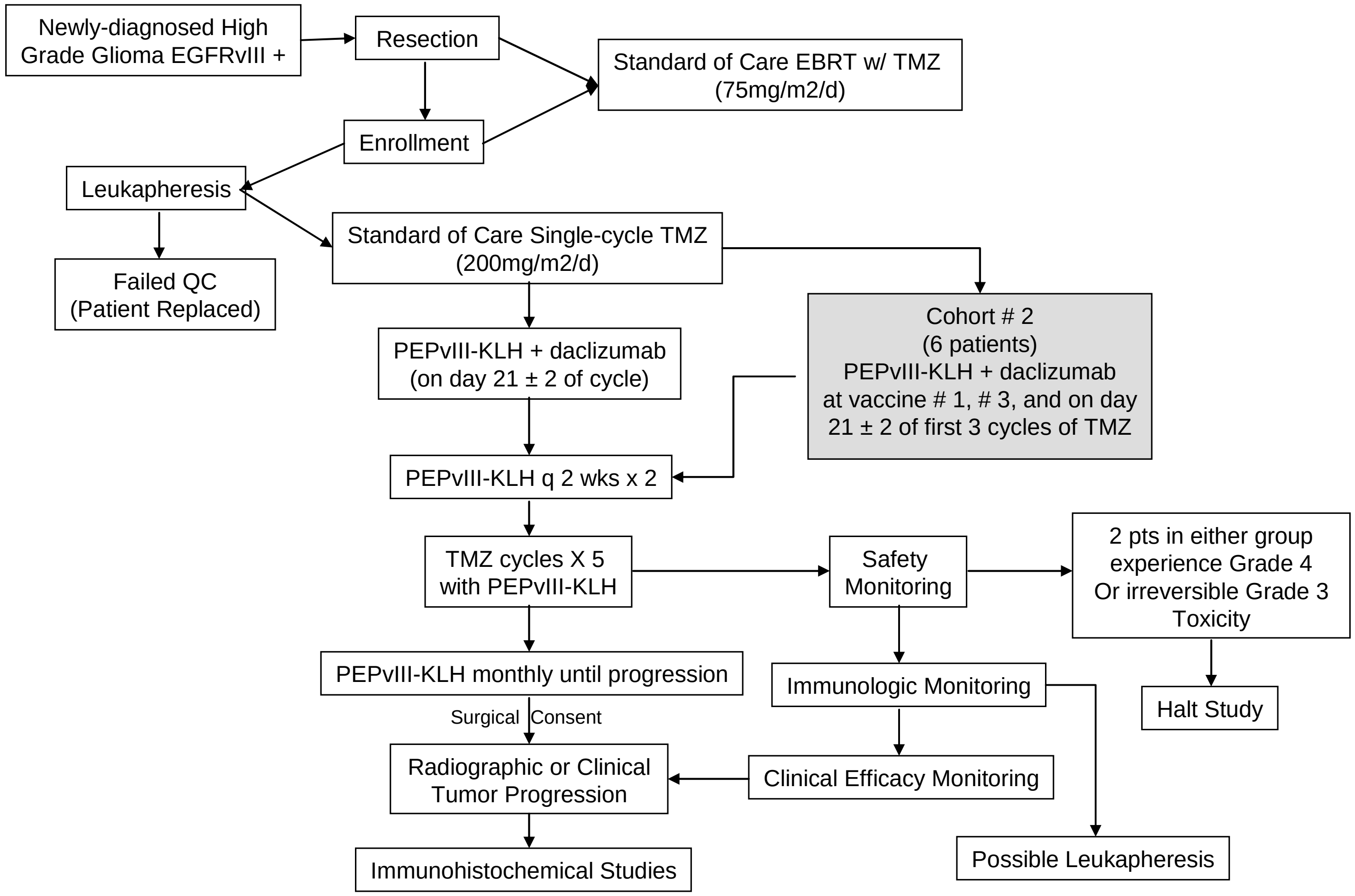

2 HYPOTHESES AND OBJECTIVES

2.1 Hypotheses

- Our primary hypothesis is that:
  - Daclizumab will inhibit the functional and quantitative recovery of T_{Regs} in patients with newly-diagnosed high grade glioma during homeostatic proliferation after therapeutic TMZ-induced lymphopenia.

- Secondary hypotheses that will be explored in this study are that:
  - Daclizumab will be safe in combination with CDX-110 in patients with newly-diagnosed high grade glioma during recovery from TMZ-induced lymphopenia.

  - The magnitude and character of humoral and cellular CD8+ and CD4+, EGFRvIII-specific T cell responses will be enhanced in adult patients with newly-diagnosed high grade glioma treated during recovery from TMZ-induced lymphopenia when daclizumab is given during the T cell recovery phase.

  - Daclizumab will not enhance antigen-specific or generalized activation-induced cell death or induce immunologic or clinical evidence of autoimmunity.

  - Daclizumab will alter the phenotype (CD56-expression), cytokine secretion profile, or cytotoxicity of CD3-CD56+ NK cells.

  - Treatment of adult patients with newly-diagnosed high grade glioma using CDX-110 during recovery from TMZ-induced lymphopenia with or without daclizumab will ultimately produce evidence of a prolonged time to progression (TTP[11]) and overall increase in median survival in these patients compared to a matched historical cohort from MD Anderson Cancer Center (provided by Dr. Amy Heimberger).

2.2 Objectives

2.2.1 Primary Objective

- To determine if daclizumab inhibits the functional and numeric recovery of T_{Regs} after therapeutic TMZ-induced lymphopenia in

[11] TTP, time to progression

the context of vaccinating adult patients with newly-diagnosed high grade gliomas using CDX-110.

2.2.2 Secondary Objectives

- To evaluate the safety of daclizumab in the context of vaccinating adult patients with newly-diagnosed high grade gliomas using CDX-110 during recovery from therapeutic TMZ-induced lymphopenia.
- To determine if daclizumab enhances the magnitude or character of CDX-110-induced cellular or humoral immune responses, inhibits or enhances activation-induced cell death, or induces immunologic or clinical evidence of autoimmunity.
- To determine if daclizumab alters the phenotype (CD56-expression), cytokine secretion profile, or cytotoxicity of CD3-CD56+ NK cells.
- To determine if daclizumab in addition to vaccination extends progression free survival compared to historical cohorts.
- To characterize immunologic cell infiltrate in recurrent tumors and seek evidence of antigen escape outgrowth.

3 BACKGROUND AND RATIONALE

3.1 Disease and Current Therapy

Malignant primary brain tumors are more common than Hodgkin's disease and account for more human deaths than melanoma or than cancer of the bladder or kidney. Despite aggressive, computer-guided tumor resection(Kelly 1992), high-dose external beam RT or brachytherapy, and multi-mechanistic chemotherapy delivered at toxic doses, most patients with malignant primary brain tumors live <15 months from the time of diagnosis, and patients with recurrent tumors usually survive <12 weeks(Walker, Alexander et al. 1978; Walker, Green et al. 1980; Shapiro 1986; Salford, Brun et al. 1988; Dinapoli, Brown et al. 1993; Stupp, Mason et al. 2005). The estimated cost of treatment for each patient with a malignant brain tumor is between $30,000 and several hundred thousand dollars annually. Thus, the annual treatment cost alone for these patients, not mentioning the lost earning potential of afflicted individuals, is greater than the entire annual budget of the National Institute of Neurological Diseases and Stroke. In fact, conventional therapy for patients with malignant brain tumor is the most expensive medical therapy per quality-adjusted life-year saved currently provided in the United States(Pickard, Bailey et al. 1990; Ekman and Westphal 2005). Moreover, the non-specific nature of conventional therapy for brain tumors often results in incapacitating damage to surrounding normal brain and systemic tissues(Imperato, Paleologos et al. 1990; Hall and Fodstad 1992). Thus, in order to be more effective, therapeutic strategies will have to precisely target tumor cells while minimizing collateral damage to neighboring eloquent cerebral cortex. The rationale for employing the immune system to target brain tumors is based on the premise that the inherent biologic specificity of immunologic reactivity could meet the clear need for more specific and precise therapy.

3.2 Vaccine Therapy for Cancer

There is extensive precedent for using therapeutic vaccines to target tumor specific or tumor relevant antigens. Many different vaccine constructs have been combined with different antigens and given via differing routes and doses with limited success at achieving tumor control. While no optimal vaccine formulation has been defined, essentially due to the lack of documented clinical efficacy with which to characterize an optimal formulation, it is clear that intradermal administration is an effective way to present antigens to the immune system, and assorted immunologically active molecules can be used to augment the immunogenicity of the vaccine.

Adjuvants frequently used with vaccination include Freund's incomplete adjuvant, bacilli Calmette-Guerin, QS-21, and diphtheria toxoid. Supplemental cytokines have been used as well for the adjuvant immunological effects (Rosenberg, Yang et al. 1998). Granulocyte macrophage-colony stimulating factor (GM-CSF) has been commonly used, as it is commercially available and well tolerated. GM-CSF is capable of stimulating macrophage function, inducing proliferation and maturation of dendritic cells, and is able to enhance T-lymphocyte stimulatory function. Intradermal administration of GM-CSF enhances the immunization efficacy at the site of administration in a dose dependent

fashion at an optimal dose of 125 μg (Kremer, Stevens et al. 2000). Significant anti-tumor immunity has been demonstrated in preclinical murine studies in which irradiated, stably transfected tumor cell lines secreting GM-CSF have protected against subsequent tumor challenge, especially against intracerebral tumors (Sampson, Ashley et al. 1997; Mach, Gillessen et al. 2000). Furthermore, the potency of GM-CSF has been demonstrated in a Phase I clinical trial in melanoma patients vaccinated with irradiated autologous melanoma cells engineered to secrete GM-CSF (Soiffer, Lynch et al. 1998). The immunization sites were intensely infiltrated with T lymphocytes, dendritic cells, macrophages, and eosinophils in 100% of evaluable patients. Extensive tumor destruction was seen in 11 of 16 patients. Both cytotoxic T cells and antibody responses were associated with this tumor destruction. Hence, GM-CSF has an extensive track record both as a growth factor and an adjuvant, is commercially available and has an acceptable toxicity profile. The experience on the ACTIVATE study supports the use of GM-CSF and sets the precedent for this phase II/III study.

3.2.1 CNS Immunologic Privilege

The CNS is an immune privileged site. Clearly, however, in situations where a CNS antigen has generated a strong immune response (Multiple Sclerosis or encephalitis) or in situations where the Blood Brain Barrier has been disrupted, it is quite possible to develop potent immune activity within CNS tissues. In the post-operative setting, where the CNS immune isolation is compromised, it is likely that an effective immune response could eliminate targeted malignant cells. Considering that an acutely ruptured Blood Brain Barrier could be revealing an area to which a patient's immune system has had only limited exposure, it is conceivable that this could be an ideal setting to generate de novo immune responses in the absence of pre-existing immune regulatory elements. The preclinical data in EGFRvIII expressing xenotransplant animal models, and the human experience with EGFRvIII vaccination to date, are consistent with effective immune control of CNS malignancy.

Experience to date in vaccinating patients against antigens expressed within the CNS has been very limited. Several other vaccine approaches against CNS tumors are in development, but no definitive data supporting this approach has been published.

3.2.2 Immunotherapy and Chemotherapy Combination

Cancer vaccines can elicit tumor-reactive humoral and cellular immune responses. To date, these responses have not reliably been able to eliminate tumor, potentially due to the multiple immune suppressive mechanism present within the malignant environment. While it has been traditionally felt that chemotherapy would compromise the cellular elements necessary to generate immune responses, recent data suggest that perhaps chemotherapy can augment immune effects through either preferential elimination of regulatory components, or amplification of antigen exposure following cytotoxic cell damage (Emens, Machiels et al. 2001; Machiels, Reilly et al. 2001; Wheeler, Das et al. 2004; Chong and Morse 2005; Emens and Jaffee 2005; van der Most, Currie et al. 2006). In general, it appears that the immune system regenerating from a cytotoxic insult may be hyperacutely activated and particularly responsive due both to increased stimulatory cytokines within the environment and reduced regulatory elements. This protocol will give vaccination during the recovery phase of each maintenance chemotherapy cycle (day

21 of 28), allowing approximately 1 week for the immune activation prior to the initiation of the next cycle of temozolomide. (See IB for lymphocyte recovery data)

3.2.3 Immunotherapy and Steroids

Glioblastoma patients are frequently treated with steroids for alleviation of neurologic dysfunction and pain that results from vasogenic edema. Lower dose steroids are often also incorporated into the chemoradiation regimen that is given in the adjuvant setting to attenuate the toxicity of radiation. The steroids can be given at high acute doses, but are tapered as rapidly as clinically feasible in order to limit the multiple consequences.

Steroids are known to be lympholytic and immune suppressive, particularly with high or prolonged dosing. In general, vaccination in the setting of steroid therapy is avoided. There are data that steroids may reduce the immune repertoire and reduce an immune response following vaccination, but may not eliminate it completely, and are unlikely to induce a tolerant state. Data in the setting of vaccination for infectious disease suggests that humoral responses may remain intact even in the presence of corticosteroids (de Roux, Schmidt et al. 2004; de Roux, Marx et al. 2006; Kapetanovic, Saxne et al. 2006). An evaluation of the effect of corticosterone on dendritic cell function revealed a failure in the generation of antigenic peptides in the presence of corticosteroid therapy (Truckenmiller, Princiotta et al. 2005; Truckenmiller, Bonneau et al. 2006). It is possible that the CDX-110 vaccine, which presents a processed immunogenic peptide, may not be subject to this effect. Thus, while vaccination in the setting of prolonged steroids is to be discouraged, it is quite possible that it may still be effective.

3.3 EGFRvIII

Most well-characterized tumor antigens are over-expressed normal proteins which have triggered tolerance to varying degrees. This may limit their effectiveness as tumor rejection antigens and poses a higher risk of autoimmunity induction if effectively targeted(Gilboa 1999; Gilboa 2004). Conversely, tumor-specific mutations in somatic genes produce truly tumor-specific proteins that would be **less prejudiced by central tolerance and less likely to induce autoimmunity**. Some studies also suggest that the autonomous immune response to human tumors is dominated by such neoantigens(Lennerz, Fatho et al. 2005). These mutations, however, tend to arise randomly as a result of the genetic instability(Lengauer, Kinzler et al. 1998; Loeb 2001) of tumors and as such tend to be individually patient-specific, incidental to the oncogenic process, and perhaps too subtle for immunologic recognition by humoral, and perhaps even, cellular immunity.

EGFRvIII, however, is a frequent, well-characterized, and consistent tumor-specific mutation (Fig. 1) , central to the neoplastic process, that consists of an in-frame deletion of 801 base pairs from the extracellular domain of the epidermal growth factor receptor (EGFR) that splits a codon and produces a novel glycine (*GLY*) at the fusion junction(Libermann, Nusbaum et al. 1985; Bigner, Humphrey et al. 1990). This mutation encodes a 145 kDa(Wong, Bigner et al. 1987; Wong, Ruppert et al. 1992) **continuatively active tyrosine kinase**(Batra, Castelino-Prabhu et al. 1995; Chu, Everiss et al. 1997) that **enhances tumorgenicity**(Nishikawa, Ji et al. 1994; Batra, Castelino-Prabhu et al. 1995; Moscatello, Montgomery et al. 1996; Huang, Nagane et al. 1997) **and migration**(Boockvar, Kapitonov et al. 2003; Pedersen, Tkach et al. 2004) and confers **radiation and chemotherapeutic resistance**(Nagane, Coufal et al. 1996; Montgomery, Guzman et al. 2000; Lammering, Hewit et al. 2003; Lammering, Hewit et al. 2004; Lammering, Valerie et al. 2004; Li, Yuan et al. 2004) to tumor cells. In addition, cells appear to be come “addicted”(Weinstein 2002) to the signaling pathways

induced by EGFRvIII and die without it(Mellinghoff, Wang et al. 2005). The EGFRvIII mutation is **most frequently seen in patients with GBM**(Sugawa, Ekstrand et al. 1990; Ekstrand, James et al. 1991; Wong, Ruppert et al. 1992; Wikstrand, Hale et al. 1995; Frederick, Wang et al. 2000; Aldape, Ballman et al. 2004; Heimberger, Hlatky et al. 2005), but has been **found in a broad array of other common cancers**(Garcia de Palazzo, Adams et al. 1993; Moscatello, Holgado-Madruga et al. 1995; Wikstrand, Hale et al. 1995; Olapade-Olaopa, Moscatello et al. 2000; Ge, Gong et al. 2002; Luo, Gong et al. 2003; Okamoto, Kenyon et al. 2003; Aldape, Ballman et al. 2004; Cunningham, Essapen et al. 2005; Heimberger, Hlatky et al. 2005) including breast carcinoma(Wikstrand, Hale et al. 1995; Ge, Gong et al. 2002; Luo, Gong et al. 2003), non-small cell lung carcinoma(Garcia de Palazzo, Adams et al. 1993; Wikstrand, Hale et al. 1995; Okamoto, Kenyon et al. 2003), colon carcinoma(Cunningham, Essapen et al. 2005), head and neck carcinoma, and prostate adenocarcinoma(Olapade-Olaopa, Moscatello et al. 2000). The new glycine inserted at the fusion junction of normally distant parts of the extracellular domain results in a **tumor-specific epitope not found in any normal adult tissues**(Humphrey, Wong et al. 1990). The exquisite tumor-specificity of EGFRvIII; its clonal expression in GBMs and other common tumors; its absence in any normal adult tissues; and its importance in the pathobiology of tumors make **EGFRvIII an ideal target for anti-tumor immunotherapy**(Humphrey, Wong et al. 1990; Garcia de Palazzo, Adams et al. 1993; Moscatello, Holgado-Madruga et al. 1995; Wikstrand, Hale et al. 1995).

3.4 CDX-110

CDX-110 is a 14 amino acid peptide chemically conjugated to Keyhole Limpet Hemocyanin (KLH). The peptide sequence is derived from the extracellular portion of the epidermal growth factor receptor mutant know as EGFRvIII. KLH is a well established carrier protein for conjugated peptide vaccines. CDX-110 is designed to induce or enhance immune responses to EGFRvIII to inhibit tumor growth or destroy tumor cells that express EGFRvIII.

3.4.1 Rationale for CDX-110

CDX-110 is a vaccine based on a peptide from a variant of the epidermal growth factor known as EGFRvIII. The epidermal growth factor receptor or EGFR has been shown to be essential for the growth of multiple human tumors. Activation of EGFR can induce cell proliferation, angiogenesis, invasion/metastasis, and the inhibition of apoptosis (Woodburn 1999) (Baselga 2001). In fact, the EGFR gene is often amplified and mutated in a high percentage of tumors, including GBMs, breast carcinomas, non-small cell lung carcinomas, and ovarian tumors (Libermann, Nusbaum et al. 1985). Several compounds that specifically target EGFR and other family members are currently being used for the treatment of cancer patients. However, the presence of EGFR on many normal tissues in the body leads to side effects with these drugs which can compromise their utility and efficacy. It also limits the use of EGFR as a target for active immunization. EGFRvIII is the most common mutated form of the natural EGFR and is present in multiple cancer types, expressed at the surface of tumor cells. Unlike EGFR, EGFRvIII has not been detected at a significant level in normal tissues.

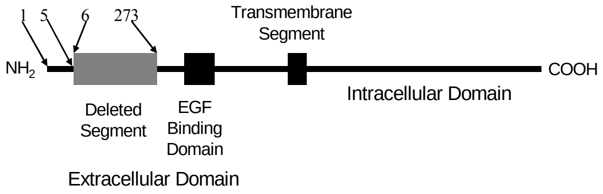


```
1               5   6                 273
    LEU-GLU-GLU-LYS-LYS-VAL-CYS-...-PRO-ARG-ASN-TYR-VAL-VAL-THR-ASP-HIS
EGFR Amino Acid Sequence

    CTG-GAG-GAA-AAG-AAA-GTT-TGC-...-CCC-CGT-AAT-TAT-GTG-GTG-ACA-GAT-CAC
EGFR cDNA Sequence

    CTG-GAG-GAA-AAG-AAA-GGT-AAT-TAT-GTG-GTG-ACA-GAT-CAC
EGFRvIII cDNA Sequence

    LEU-GLU-GLU-LYS-LYS-GLY-ASN-TYR-VAL-VAL-THR-ASP-HIS
EGFRvIII Amino Acid Sequence

    LEU-GLU-GLU-LYS-LYS-GLY-ASN-TYR-VAL-VAL-THR-ASP-HIS-CYS-KLH
PEPvIII-KLH Sequence
```

Fig. 1: **EGFRvIII Sequence**—Comparative sequences for wild type EGFR, the EGFRvIII mutation, and the EGFRvIII-specific peptide sequence PEPvIII-KLH.

EGFRvIII is a mutated form of the natural EGFR that occurs through alternative splicing. Due to the deletion in EGFRvIII, a novel protein sequence is created that is not present in EGFR (Figure 1). The unique peptide sequence (13 amino acids including the glycine formed at the junction) is the antigen for the CDX-110 vaccine. Previous studies have demonstrated that antibodies and T cells can specifically recognize the unique peptide formed in EGFRvIII.

EGFRvIII has additional properties that make it an attractive cancer target. Unlike EGFR, EGFRvIII is a molecule that can directly lead to cancer through its well documented oncogenic properties - it provides a constant growth signal to tumor cells which bear it(Tang, Gong et al. 2000). Consequently, it has been shown that cells producing EGFRvIII have an enhanced capacity for unregulated growth, survival, invasion, and recruitment of new tumor blood vessels. The EGFRvIII protein contains a unique sequence at the splice junction that is not found in EGFR. This sequence was used to generate the immunotherapeutic molecule, PEPvIII-KLH, which is able to induce a tumor specific immune response. The efficacy of this approach has been shown in preclinical models of EGFRvIII positive brain cancer(Heimberger, Crotty et al. 2003).

Peptide vaccination requires adjuvants to provide immune stimulation for generating significant immune responses. In addition, peptides are rapidly degraded in vivo unless conjugated to proteins that can extend the bioavailability of the peptide. The protein known as KLH is a well established carrier protein for conjugated peptide vaccines (Harris and Markl 1999). KLH is used clinically as an immunostimulant, and is part of several conjugated vaccines currently in clinical development. In addition to increasing the stability of the peptide after administration, KLH induces helper T-cell responses, which can enhance the immune responses to the EGFRvIII peptide.

Adjuvants frequently used with vaccination include Freund's incomplete adjuvant, bacilli Calmette-Guerin, QS-21, and diphtheria toxoid. Supplemental cytokines have been used as well for the adjuvant immunological effects (Rosenberg, Yang et al. 1998). Granulocyte macrophage-colony stimulating factor (GM-CSF) has been commonly used, as it is commercially available and well tolerated. GM-CSF is capable of stimulating macrophage function, inducing proliferation and maturation of dendritic cells, and is able to enhance T-lymphocyte stimulatory function. Intradermal administration of GM-CSF enhances the immunization efficacy at the site of administration in a dose dependent

fashion at an optimal dose of 125 μg (Kremer, Stevens et al. 2000). Significant anti-tumor immunity has been demonstrated in preclinical murine studies in which irradiated, stably transfected tumor cell lines secreting GM-CSF have protected against subsequent tumor challenge, especially against intracerebral tumors (Sampson, Ashley et al. 1997; Mach, Gillessen et al. 2000). Furthermore, the potency of GM-CSF has been demonstrated in a Phase I clinical trial in melanoma patients vaccinated with irradiated autologous melanoma cells engineered to secrete GM-CSF (Soiffer, Lynch et al. 1998). The immunization sites were intensely infiltrated with T lymphocytes, dendritic cells, macrophages, and eosinophils in 100% of evaluable patients. Extensive tumor destruction was seen in 11 of 16 patients. Both cytotoxic T cells and antibody responses were associated with this tumor destruction. Hence, GM-CSF has an extensive track record both as a growth factor and an adjuvant, is commercially available and has an acceptable toxicity profile. The experience on the ACTIVATE study supports the use of GM-CSF and sets the precedent for this phase I study.

3.4.2 Pre-clinical Experience with EGFRvIII Vaccination

Active immunotherapy targeting EGFRvIII can lead to anti-tumor responses. Monoclonal antibodies specific for EGFRvIII have demonstrated activity in tumor xenograft models (Sampson, Crotty et al. 2000; Luwor, Johns et al. 2001) suggesting that humoral responses to an EGFRvIII-peptide vaccine can directly impact on tumor growth. In addition, cellular responses to EGFRvIII may be an important component to treatment of GBM. Dendritic cell vaccination strategies, as well as KLH-conjugated peptides, are effective means for eliciting T cell immunity in mice and protecting against EGFRvIII expressing tumors (Figure 2). Importantly, Heimberger et al, using the PEP3-KLH vaccine (also referred to as PEP-3-KLH) have shown these vaccination strategies were also effective in enhancing survival of mice when administered after tumor transplantation (Heimberger, Crotty et al. 2000; Heimberger, Archer et al. 2002; Heimberger, Crotty et al. 2003). The EGFRvIII-expressing intracerebral tumors were introduced to mice prior to subcutaneous administration of PEP3-KLH vaccine or control treatments (arrows)(Heimberger, Crotty et al. 2003)

In addition to proof of concept studies in animal models, there is evidence that humans can generate EGFRvIII specific immune responses. Antibodies to EGFRvIII have been found in the serum of patients with GBM, demonstrating that the patients' immune systems can recognize EGFRvIII (Wikstrand, Hale et al. 1995; Moscatello, Ramirez et al. 1997). Recently, T-cell and antibody responses to EGFRvIII have also been documented in some breast cancer patients(Purev, Cai et al. 2004).

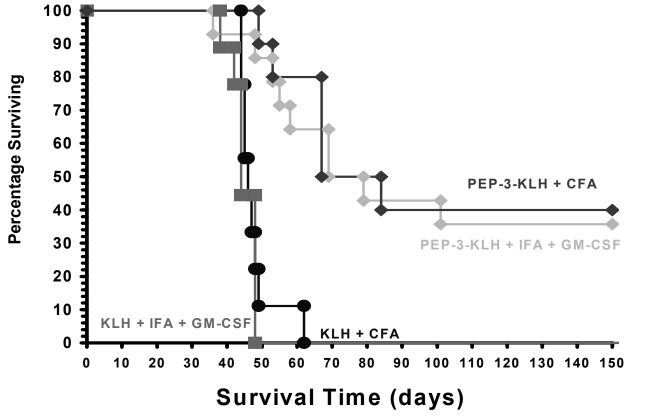

Fig. 2. PEPvIII-KLH vaccine enhances survival of mice with established intracerebral tumor.

Thus, tumor expression of EGFRvIII appears to be able to elicit measurable immune responses to this antigen that could be amplified with vaccination. Cytolytic T lymhocytes (CTLs) can also contribute to tumor elimination, and Wu et al, have demonstrated that EGFRvIII-specific CTLs can be generated from HLA-A2+ peripheral blood mononuclear cells (PBMC) when exposed to a nine amino acid sequence that is contained within the PEPvIII-KLH peptide (Wu, Xiao et al. 2006). These CTL had specific reactivity against EGFRvIII expressing glioma cells.

The pre-clinical data suggest that the EGFRvIII peptide can elicit effective anti-tumor responses in animals. Importantly, systemic immunization approaches were effective against intracerebral tumors.

3.4.3 Clinical Experience

PEP3-KLH has been evaluated in a single institution Phase I (VICTORI) and a randomized, multi-institutional Phase II study (ACTIVATE) (Heimberger, Hussain et al. 2006). These studies have demonstrated that vaccinations with an EGFRvIII-specific peptide **induce T- and B-cell immunity**, produce **complete radiographic responses in all patients** with residual tumor (Fig. 3), universally **eliminate EGFRvIII-expressing cells (See Appendices)(Heimberger, Hussain et al. 2006; Heimberger, Hussain et al. 2006)**, and **extend median survival to >29 months**(Heimberger, Hussain et al. 2006; Heimberger, Hussain et al. 2006) (Fig. 4) which compares favorably with recently published trials evaluating newly-diagnosed patients with GBM treated with GLIADEL® (13.9 months)(Westphal, Hilt et al. 2003); radiation and concurrent TMZ (14.6 months)(Stupp, Mason et al. 2005); or radiolabeled anti-tenascin monoclonal antibodies performed at Duke University (18.3 months)(Boskovitz, Wikstrand et al. 2004).

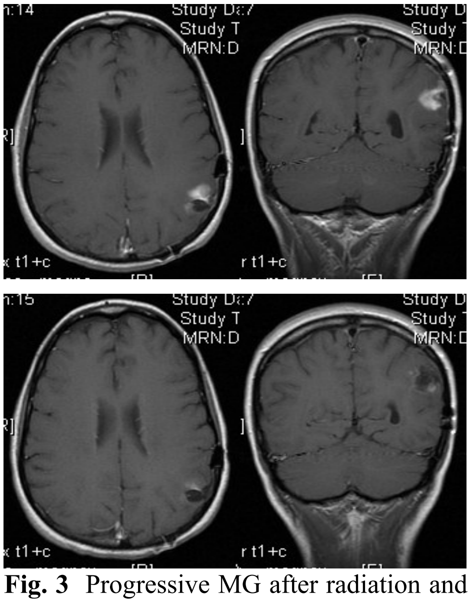

Fig. 3 Progressive MG after radiation and temozolomide (top) with nearly complete response, 3 months after PEPvIII-pulsed DC vaccine (bottom).

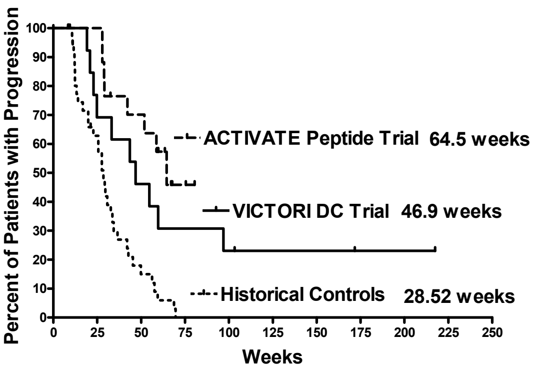

Fig. 4 TTP comparison in patients treated with PEPvIII-KLH loaded DCs (VICTORI Trial), patients treated with PEPvIII-KLH alone (ACTIVATE Trial), and a historical control group that had a 95% resection of an EGFRvIII-positive tumor. There was no significant difference in the TTP between the patients in the VICTORI and ACTIVATE trials, but both were significantly better than historical controls (*P*<0.001).

In the ACTIVATE Trial 23 patients with newly-diagnosed, EGFRvIII-expressing GBM who had received gross-total resection followed by conformal radiation with concurrent temozolomide (75mg/m^2/day) without tumor progression, were enrolled. PEP3-KLH (500 mcg/immunization), mixed with GM-CSF (approx. 142 mcg/immunization), were administered intradermally. Nineteen (19) patients received PEP3-KLH at two-week intervals for three (3) doses, while four (4) patients received saline in a blinded fashion for these first three vaccinations. Thereafter, all patients received monthly PEP3-KLH injections until tumor progression.

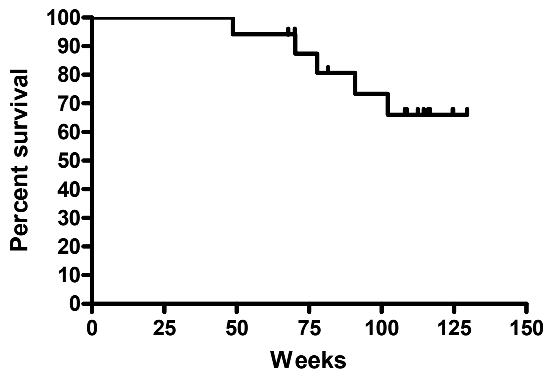

Fig. 5 Survival of patients treated with PEPvIII vaccine. Vaccine patients have a median survival of >126 weeks. This compares favorably to carmustine wafers (60 weeks)(Westphal, Hilt et al. 2003) and TMZ (63 weeks)(Stupp, Mason et al. 2005). Median time to progression (TTP) in the multi-instituional ACTIVATE trial was 64 weeks which compared favorably to a matched historical cohort (28 weeks).

Toxicity was essentially limited to local reactions and no evidence of induced autoimmunity was reported. All patients experienced mild injection site reactions with urticaria, while one patient experienced mild fever thought possibly related to PEP3-KLH. A single patient experienced radiographic changes suggestive of focal demyelination in the pons and corpus collosum after an eleventh vaccination but remained entirely asymptomatic. The radiographic abnormalities improved spontaneously despite continued vaccination. Another patient was reported to have a grade 3 allergic reaction following vaccination, but had a similar experience in association with an MRI study – both consistent with an anxiety process. The investigator discontinued vaccination in this patient despite the patient's desire to continue.

Delayed-type hypersensitivity (DTH) testing showed no response to components of PEP-3-KLH at baseline, but at 6 months, 62% of patients were immunologically reactive to at least one component of the vaccine. The vaccination induced a statistically significant increase in PEP3-specific γ-IFN producing T cells, and increased EGFRvIII-specific antibody titers that correlated with time to tumor progression (TTP). Overall, 38% of the vaccine patients produced EGFRvIII specific humoral immune responses that were at least five-fold greater than pre-vaccine levels (p=0.0008).

Median TTP in the vaccine-treated patients was 12.1 months, comparing favorably with a matched, unvaccinated historical cohort of thirty-nine (39) patients treated at MD Anderson (7.1 months; p=0.0058), and with those obtained using the accepted standard of care of concurrent temozolomide and radiation followed by adjuvant temozolomide (6.9 months*)* (Cohen, Johnson et al. 2005; Stupp, Mason et al. 2005). Final median survival has not been reached at median follow-up of 19 months, which again compares favorably with the matched historical cohort (13 months) and the standard of care therapy group (14.6 months). Six (6) patients experienced recurrence of GBM and were evaluated by immunohistochemistry. Of these, none were found to maintain expression of EGFRvIII,

consistent with elimination of EGFRvIII expressing cells, or loss of expression of the EGFRvIII antigen.

3.5 Cross-Priming

Priming of naïve $CD8^+$ and $CD4^+$ T-cell responses against tumor-derived antigens requires the processing and presentation of antigenic peptides by antigen presenting cells (APC)(Huang, Golumbek et al. 1994; Armstrong, Pulaski et al. 1998; Sotomayor, Borrello et al. 2001). The **milieu of cytokines and cells present at the site of antigen uptake, mediate whether the functional outcome of such cross-priming is the activation of effector cells or induction of antigen-specific tolerance**(Hoffmann, Meidenbauer et al. 2000; Albert, Jegathesan et al. 2001; Kammerer, Stober et al. 2002; Schnurr, Scholz et al. 2002; Scheffer, Nave et al. 2003; van Mierlo, Boonman et al. 2004). Numerous studies have demonstrated that the presentation of tumor-derived antigens by host APCs often occurs in the context of tolerigenic signals, thus attenuating productive immune responses against the growing tumor(Ferguson, Herndon et al. 2002; Wolkers, Brouwenstijn et al. 2004; Boonman, van Mierlo et al. 2005; Otahal, Hutchinson et al. 2005). While the trafficking patterns of professional APC into and out of the CNS have not been well elucidated, cross-priming against antigens derived from intracranial tumors has been shown to occur and lead to the generation of systemic and intracranial anti-tumor immunity(Plautz, Mukai et al. 2000; Calzascia, Di Berardino-Besson et al. 2003; Velicu, Han et al. 2006). Mechanisms that enhance cross-priming, including delivery of inflammatory signals during antigen uptake(Hoffmann, Meidenbauer et al. 2001; Gri, Chiodoni et al. 2002; Tatsumi, Huang et al. 2003; Zimmermann, Bondanza et al. 2004), induction of cell death pathways in tumor cells(Nowak, Lake et al. 2003; Rovere-Querini and Dumitriu 2003; Correale, Cusi et al. 2005; Shi, Cao et al. 2006), or the removal of $\mathbf{T_{Regs}}$ that **suppress the efficiency of cross-priming**(Haeryfar, DiPaolo et al. 2005), have all been demonstrated in animal models to enhance the induction of anti-tumor immunity and to mediate therapeutic benefit in established tumor models(Arina, Tirapu et al. 2002).

3.6 Autoimmune Encephalomyelitis

Antitumor vaccinations have been shown to be quite capable of initiating significant autoimmune responses in murine models, and there has been one incident of a spontaneous generalized vitiligo that occurred after a second intravenous infusion of dendritic cells (DCs) in a patient with melanoma. Although our group and others have demonstrated that DCs loaded with unselected tumor-derived antigens induce potent, specific, and clinically effective immune responses against brain tumors in rodent models without the induction of autoimmune reactivity(Ashley, Faiola et al. 1997; Liau, Black et al. 1999; Heimberger, Crotty et al. 2000; Heimberger, Archer et al. 2002), and although no autoimmune reactions have been identified in human immunotherapy trials in patients with MGs(Yu, Wheeler et al. 2001; Kikuchi, Akasaki et al. 2004; Liau, Prins et al. 2005), immunization in preclinical studies has only been effective when given before tumor challenge or in the context of very small established tumors. These data suggest that for DC-based immunotherapy to be effective in the context of large human tumors, a very strong and sustained antitumor immune response will be required(Ochsenbein, Klenerman et al. 1999). In animal models, when such responses have been generated

against tumor-associated antigens that are shared with host cells, severe and clinically significant autoimmune disease has occasionally resulted(Ludewig, Ochsenbein et al. 2000).

Despite the fact, that no clinical or radiological evidence of experimental autoimmune encephalomyelitis (EAE[12]) was induced by the immunization therapies in any of the preclinical experiments or the clinical trials outlined above, it must be acknowledged that one potential complication of immunotherapy, especially once optimized, is the induction of clinically significant autoimmunity. In fact, many published manuscripts have demonstrated the induction of clinically significant autoimmunity, including EAE, in preclinical studies employing DC-based or other immunization strategies(Borrow, Cornell et al. 1995; Dittel, Visintin et al. 1999; Bourquin, Iglesias et al. 2000; Ludewig, Ochsenbein et al. 2000). Although this may even be a desirable outcome for nonessential tissues, such as the prostate, when infiltrated by tumor, an autoimmune encephalomyelitis could be a lethal consequence of any non-specific active immunotherapy approach for primary intracerebral tumors where the target antigens are ill-defined and not tumor-specific.

In light of the documented expression of normal and fetal brain antigens on human glioma cell lines(Wahlstrom, Linder et al. 1973), and brain tumor tissue(Siris 1936; Slagel, Wilson et al. 1969; Wickremesinghe and Yates 1971), active, specific immunization strategies with uncharacterized brain tumor antigens that are not tumor-specific poses the theoretical risk of inducing an uncontrolled autoimmune response against normal CNS antigens. Such a response would be similar to EAE, which is an acute or chronic, autoimmune, inflammatory demyelinating disease mediated primarily by antigen-specific CD4+ T-cells. Myelin basic protein is the most common known antigenic trigger, but myelin proteolipid protein(Waksman, Porter et al. 1954; Wikstrand and Bigner 1979), myelin oligodendrocyte glycoprotein(Tuohy, Lu et al. 1988), glial fibrillary acidic protein, and S-100β(Linington, Berger et al. 1993), are also sufficient antigens for the induction of EAE.

The susceptibility of humans to the induction of EAE was discovered accidentally when patients were immunized against rabies with spinal cords from rabbits that were infected with the rabies virus(Pasteur 1885; Remlinger 1904; Remlinger 1905; Stuart and Krikorian 1930; Wekerle, Kojima et al. 1994). The toxic component found in these immunizations was subsequently proven to be related to encephalitogenic components contained within the spinal cord preparation and not the virus(Stuart and Krikorian 1928). EAE has also been induced in monkeys after repeated injections of homogenized CNS tissue(Rivers and Schwentker 1935). It was not until the use of strong adjuvants such as complete Freund's adjuvant (CFA[13]), that EAE could be easily and reproducibly created in animals in this manner(Kabat, Wolf et al. 1947). Since that time, it has been shown that EAE can be readily induced in the various species of rats, guinea pigs, mice, sheep, and monkeys after a single injection of CFA and either autologous or heterologous CNS tissue homogenate(Bigner, Pitts et al. 1981).

Given the range of protocols that routinely use immunization with CNS tissue for the production of lethal EAE in non-human primates(Bigner, Pitts et al. 1981), and the documented susceptibility of humans to EAE, the induction of such autoimmune responses is a legitimate concern. Some apprehension regarding EAE is also warranted

[12] EAE, experimental allergic encephalitis
[13] CFA, complete Freund's adjuvant

on the basis of previous active, specific immunotherapy trials in humans with brain tumors. Although no cases of EAE were reported in some human studies(Ommaya 1976; Albright, Seab et al. 1977; Mahaley, Bigner et al. 1983; Bullard, Thomas et al. 1985), and protocols have been developed for safe active, specific immunotherapy with glioma-derived cells in primates(Wikstrand and Bigner 1981), careful review of several other studies(Bloom, Peckham et al. 1973) and(Trouillas and Lapras 1970; Trouillas 1973) reveal one possible case of EAE within each study. Thus, the risk of EAE, or other similar and potentially lethal autoimmune responses, may severely limit the efficacy of active, ***non-specific*** immunotherapy for malignant brain tumors. Thus targeting tumor-specific antigens, such as EGFRvIII or the more recently discovered viral antigens associated with PEPvIII and KLH that are present within MGs, would enhance the safety of early trials in this field.

Depletion or neutralization of $CD25^+$ T_{Regs} prior to adoptive T-cell transfer into immunocompromised mice results in a reproducible spectrum of autoimmune disease including thyroiditis, prostatitis, gastritis, orchitis, oophoritis, and insulinitis(Taguchi and Nishizuka 1987; Taguchi, Kontani et al. 1994; Seddon and Mason 1999; Seddon and Mason 1999; Bagavant, Thompson et al. 2002). However, removal of $CD25^+$ cells alone has not proven sufficient for eliciting EAE(Fecci, Sweeney et al. 2006).

Active immunization then in patients with GBM risks inducing an uncontrolled autoimmune response similar to EAE. Although we have not seen autoimmune manifestations with our tumor-specific EGFRvIII-targeted vaccine, our proposal to unleash secondary immune responses through IL-2Rα blockade of T_{Regs} may risk the induction of such deleterious autoimmune responses. Thus, this approach must be explored with particular attention to the propensity for autoimmune manifestations in treated patients.

3.7 T Cell Homeostatic Proliferation

Homeostatic proliferation is a mechanism by which the body maintains normal physiologic levels of circulating lymphocytes. Within the normal host, homeostatic proliferation involves the relatively slow replication of naïve and memory T-cells along with the generation of new lymphocytes from the bone marrow to maintain normal numbers of T-cells (and other lymphocytes).

Under conditions that induce profound lymphopenia, however, such as during non-myeloablative chemotherapy, naïve and memory T-cells enter a rapid proliferative state in order to replenish the drastically diminished lymphocyte numbers. During physiologic hematopoietic recovery, new T-cells must be regenerated from the bone marrow or by division of the few remaining T-cells in the peripheral lymphoid organs or circulation and is a prolonged process that may take weeks to months to recover normal T-cell counts. Following a period of lymphopenia, there is a spontaneous repopulation of the host's T-cells to re-establish the T-cell repertoire(Cho, Rao et al. 2000). Tanchot *et al.*(Tanchot, Rosado et al. 1997) propose that T-cells remaining after lymphodepletion compete for a finite amount of cytokine and antigenic stimulation. Adoptively transferred lymphocytes when transplanted into lymphopenic hosts, also undergo rapid proliferation and differentiation into effector and memory cells and can provide enhanced protection against tumor outgrowth in experimental animals(Wrzesinski and Restifo 2005). Furthermore, populations of tumor-specific lymphocytes when transferred into

lymphopenic melanoma patients have been shown to be capable of eradicating disseminated malignant disease and maintaining high levels of tumor-specific memory T-cells in the circulation of treated patients(Dudley, Wunderlich et al. 2002; Dudley, Wunderlich et al. 2005; Hughes, Yu et al. 2005; Zhou, Dudley et al. 2005). These studies indicate that homeostatic proliferation may be leveraged to augment antitumor immunity by using T-cells educated to be reactive against tumor either through *ex vivo* expansion or possibly antitumor vaccination *in vivo*.

Recently it has also been demonstrated that depletion of the host's native lymphocyte pool with subsequent vaccination during endogenous homeostatic T-cell reconstitution enhanced the protection of mice bearing established extracranial tumors(Asavaroengchai, Kotera et al. 2002). Similarly, adoptive reconstitution with tumor-reactive T-cells after intentional non-myeloablative lymphodepletion allowed for a marked preferential expansion and maintenance of tumor-specific transferred T-cells resulting in dramatic clinical responses, along with some autoimmune toxicity, in patients with advanced malignant melanoma(Dudley, Wunderlich et al. 2002). Later studies have also indicated that *ex vivo* manipulation of the adoptively transferred cells, for example by depletion of T_{Regs}, further enhances the reconstitution of the host with tumor antigen-specific T-cells(Antony, Piccirillo et al. 2005). These approaches take advantage of still undefined homeostatic forces that provide an environment conducive to T-cell proliferation during recovery from lymphopenia. We propose to use vaccination in patients with treatment-induced lymphopenia in order to determine whether high levels of PEPvIII-KLH-specific, tumor reactive T-cells can be generated and maintained in the circulation of these patients.

Our previous studies have demonstrated the safety of vaccination targeting the EGFRvIII mutation in patients with MGs, and we expect the targeting of PEPvIII-KLH antigens to be well tolerated. There is considerable clinical experience with T-cell based immunotherapy targeting PEPvIII-KLH in bone marrow and organ transplant patients and these studies have demonstrated the safety and efficacy of immunotherapy against PEPvIII-KLH, even in CNS-involved disease. Lymphopenia will be induced in our patient population using standard doses of TMZ, an alkylating agent used routinely in the treatment of MGs at our institution and abroad as outlined below.

Lymphodepletion may be advantageous in patients with GBMs for several reasons. First, lymphodepletion will eliminate the defective T-cells that are well-described in patients with GBM(Brooks, Netsky et al. 1972). Second, lymphodepletion may eliminate T_{Regs}, an inhibitory subset shown by us to be significantly upregulated in patients with GBMs and, in fact TMZ may preferentially deplete these suppressive cells(Su, Sohn et al. 2004). By transferring tumor-specific T-cells into such an environment, especially in the context of tumor-specific vaccination, the tumor-specific T-cells may have a selective advantage. It has also been shown in lymphopenic hosts, that proliferating T-cells differentiate directly into memory T-cells capable of rapid and intense response to antigen re-exposure(Tanchot, Rosado et al. 1997). Grossman and Paul propose that T-cells respond to lymphopenia by tuning their activation threshold down to a level at which T-cells are driven to proliferate, even in the absence of foreign antigens(Grossman and Paul 2000). Also in line with our thinking, others(Dudley, Wunderlich et al. 2002; Hu, Poehlein et al. 2002) propose that, if tumor antigens are present during this repopulation, the population of T-cell could be skewed to create a tumor antigen-targeted

response. Homeostatic proliferation can then be augmented with autologous transfer of antigen-specific activated T-cells to skew the reconstituted pool resulting in an even more homogeneous T-cell repertoire that preferentially targets tumor antigen. In fact, in other studies, it has been shown that transferred T-cells can expand dramatically in the host to the point where they constitute up to 90% of the host's T-cell repertoire and can be maintained for months following transfer(Dudley, Wunderlich et al. 2002).

3.8 Treatment-Induced Lymphopenia from Temozolomide

TMZ, a methylating agent with good blood-brain barrier penetration, has recently been shown to increase survival by a small, but statistically significant, 2.5 months in a subset of patients with newly-diagnosed GBM if given in conjunction with RT following initial resection of the tumor(Stupp, Dietrich et al. 2002; Hegi, Diserens et al. 2005; Stupp, Mason et al. 2005). Leukopenia is essentially the only known human toxicity of TMZ. Although initially counter-intuitive, this TMZ-induced leukopenia may actually be advantageous in treating patients with immunotherapy due to the subsequent homeostatic proliferation it induces. In this protocol patients will receive standard of care doses of TMZ concurrent with RT following initial resection of the tumor. We believe that the myelosuppression induced by therapeutic TMZ treatment, if carefully timed ***before*** vaccination or adoptive reconstitution with T-cells, will actually enhance the proliferation and maintenance of these tumor-specific T-cells through the natural forces that drive T-cell homeostatic proliferative recovery. In addition, TMZ has also been shown to preferentially deplete T_{Regs}(Su, Sohn et al. 2004). Thus, this combination strategy will uniquely exploit the toxicity of one effective therapy for GBMs, TMZ, to enhance another already promising therapy, immunotherapy. In preparation for this protocol, we have evaluated, in animal models, TMZ and sublethal whole body irradiation (WBI[14]), as a positive control, as methods for induction of treatment-induced lymphopenia in order to determine the ability of TMZ-induced lymphodepletion to enhance active and adoptive immunotherapy. These studies were initially performed in a murine T-cell receptor transgenic model in which the antigen-specific T-cells can be followed *in vivo* in mice receiving adoptive transfer. In this model system, we found that ALT coupled with DC vaccination (DC + ALT) is a potent mechanism for inducing antigen-specific T-cell expansion after TMZ treatment. TMZ was found to be an effective agent for inducing homeostatic proliferation of transferred CD4+ and CD8+ T-cells and for enhancing DC or peptide vaccinations with or without ALT (Fig. 6).

[14] WBI, whole body irradiation

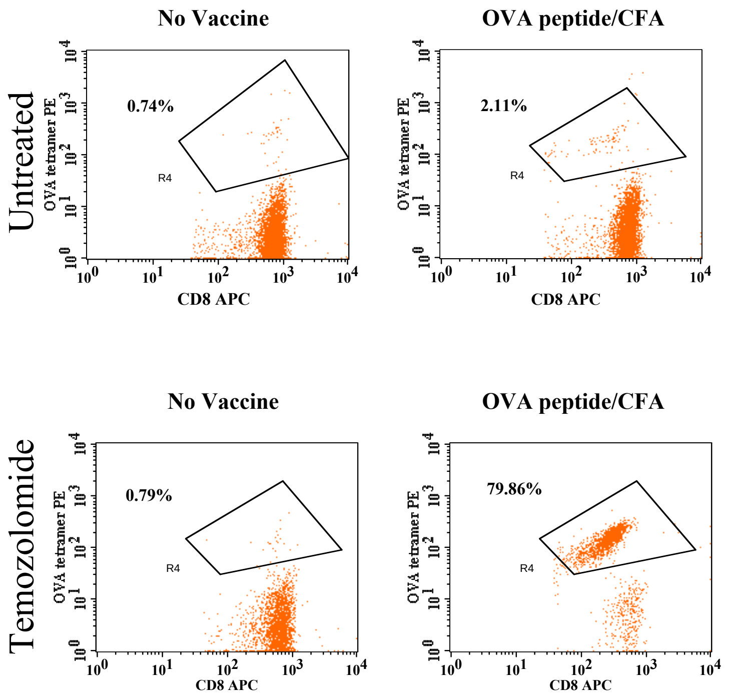

Fig. 6: Homeostatic lymphodepletion induced by temozolomide enhances antigen-specific immune responses. Untreated mice or mice receiving non-myeloablative high-dose temozolomide treatment (5 days x 60 mg/kg) were administered a mixture of OVA-specific and normal lymphocytes intravenously and received either no vaccine, vaccination with DCs transfected with OVA RNA, or OVA peptide vaccination in complete Freund's adjuvant. The peripheral blood of treated animals was collected and analyzed 6 days after vaccination for the expansion of OVA-specific T-cells in the circulation using αCD8 monoclonal antibodies and a specific tetramer. As shown above, mice treated with temozolomide prior to ALT demonstrated markedly increased T-cell expansion after vaccination compared to untreated hosts receiving ALT.

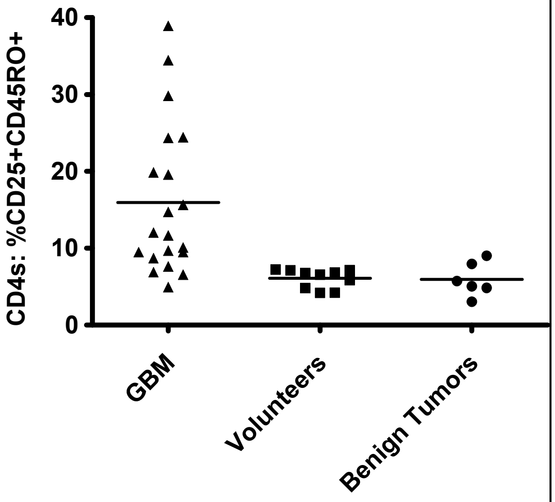

Fig. 7: Patients with newly-diagnosed GBM exhibit increased proporation of T_{regs} (CD4+CD25+CD45RO+FoxP3+ T cells) (Fecci, Mitchell et al. 2006). (See Appendix).

3.9 Immunosuppression in Patients with GBM and CD25+ T_{Regs}

A substantial barrier to the activation of antitumor immune responses in patients with GBM is their well-documented impairment of T- and B-cell immunity(Brooks, Netsky et al. 1972; Brooks, Caldwell et al. 1974; Thomas, Lannigan et al. 1975; Brooks, Roszman et al. 1976; Young and Kaplan 1976; Brooks, Roszman et al. 1977; Mahaley, Brooks et al. 1977; Menzies, Gunar et al. 1980; Roszman and Brooks 1980; Braun, Penn et al. 1982; Roszman, Brooks et al. 1982; Fontana, Hengartner et al. 1984; Miescher, Whiteside et al. 1986; Elliott, Brooks et al. 1987; Roszman, Brooks et al. 1987; Miescher, Whiteside et al. 1988; Elliott, Brooks et al. 1990; Roszman, Elliott et al. 1992). For decades, such impairment has been catalogued, but little advancement was made in

uncovering its etiology. Although immunosuppressive factors secreted by the tumor clearly play a role(Schwyzer and Fontana 1985; Kehrl, Roberts et al. 1986; Kehrl, Wakefield et al. 1986; Rook, Kehrl et al. 1986; Espevik, Figari et al. 1987; Ranges, Figari et al. 1987; Czarniecki, Chiu et al. 1988; Wahl, Hunt et al. 1988; Zuber, Kuppner et al. 1988; Baruah, Propato et al. 2006), we have recently demonstrated that a major contributor to depressed cellular immunity in patients with GBM is an increased representation of T_{Regs} among CD4+ T-cells (Fig. 7 and 8)(Fecci, Mitchell et al. 2006).

T_{Regs} are a physiologic subset of CD4+ T-cells in both mice and humans, normally comprising approximately 5-10% of this compartment(Sakaguchi, Sakaguchi et al. 1995; Suri-Payer, Amar et al. 1998; Jonuleit, Schmitt et al. 2001; Ng, Duggan et al. 2001; Yamagiwa, Gray et al. 2001) that serve to curtail the immunologic function of B-cells(Lim, Hillsamer et al. 2005; Zhao, Thornton et al. 2006), DCs(Fallarino, Grohmann et al. 2003), monocyte/macrophages(Taams, van Amelsfort et al. 2005), NKT cells(Azuma, Takahashi et al. 2003), and NK cells(Smyth, Teng et al. 2006; Ralainirina, Poli et al. 2007). They constitutively express high levels of the high-affinity interleukin (IL)-2 receptor α (IL-2Rα) (CD25) on their surface,(Sakaguchi, Sakaguchi et al. 1995) and can be identified even more specifically by expression of the intracellular transcription factor FOXP3. In vitro studies have revealed that T_{Regs} potently inhibit T-cell cytokine secretion and proliferation(Thornton and Shevach 1998; Dieckmann, Plottner et al. 2001; Jonuleit, Schmitt et al. 2001),(Fontenot, Gavin et al. 2003; Khattri, Cox et al. 2003), directly curtail the generation and expansion of endogenous or induced immune responses in vivo(Taguchi and Nishizuka 1987; Green and Webb 1993; Taguchi, Kontani et al. 1994; Sakaguchi, Sakaguchi et al. 1995; Asano, Toda et al. 1996; Seddon and Mason 1999; Salomon, Lenschow et al. 2000; Stephens and Mason 2000; Bagavant, Thompson et al. 2002), and appear to play a significant role in hindering immunity to tumor-associated antigens(Somasundaram, Jacob et al. 2002; Curiel, Coukos et al. 2004). Our own studies reveal that the removal of T_{Regs} from patient T-cells *in vitro* restores T-cell proliferative and cytokine responses to normal levels(Fecci, Mitchell et al. 2006). Similarly, inactivation of T_{Regs} in our murine gliomas model *in vitro* significantly enhances endogenous and vaccination-induced antitumor immune responses and leads to complete eradication of intracranial tumors in a stringent model (Fig. 9)(Fecci, Mitchell et al. 2006; Fecci, Sweeney et al. 2006). Together, these data strongly suggest that depletion or inactivation of T_{Regs} may be sufficient to overcome the barriers to immunotherapy.

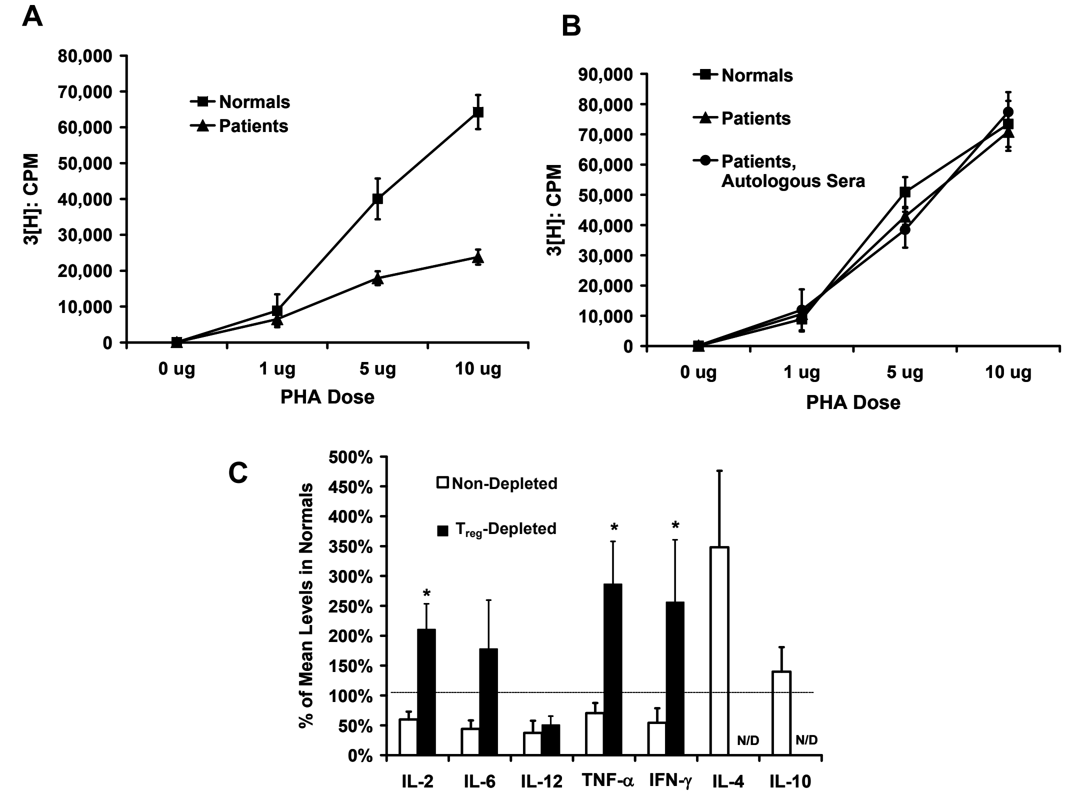

Fig. 8. Patient CD4$^+$ T-cells function normally in the absence of T_{regs}. *A*. Levels of CD4$^+$ T-cell proliferation were assessed among cells from five patients with GBM possessing CD4 proliferative defects and from three healthy controls. CD4$^+$ T-cells were cultured in equal numbers in the presence of varying concentrations of PHA, OKT3, or ConA and proliferation assessed by ^{3}[H]-thymidine incorporation. Patient CD4$^+$ T-cells were cultured in the presence of either FCS or autologous serum. Cumulative results of seven experiments with PHA as stimulator are depicted. Results were similar across all stimulators. *B*. Assays in (*A*) were repeated following removal of CD25$^+$ cells. Differences in proliferation among patients and controls were erased (p=0.689), even when patient cells were cultured in the presence of autologous serum. *C*. T_{reg} depletion relieves T_H2 shift in cytokine production. CD4$^+$ T-cells were purified from patients with GBM (n=5) and healthy controls (n=3). Cells left non-depleted or depleted of CD25$^+$ cells were cultured in equal numbers using PHA as stimulator. Supernatants were collected and analyzed in duplicate for levels of IL-2, IL-4, IL-6, IL-10, IL-12p70, TNF-α, and IFN-γ. Levels of production of each cytokine by patients were expressed as percentages of that by normal CD4$^+$ T-cells to depict differences in profiles. Cumulative results from three experiments are shown. Prior to depletion, patient CD4$^+$ T-cells produce diminished levels of the T_H1 cytokines IL-2, IL-6, IL-12p70, TNF-α, and IFN-γ, but increased levels of the T_H2 cytokines IL-4 and IL-10. This skew is almost completely eliminated upon depletion of CD25$^+$ cells (p values for shifts in production following depletion: IL-2: 0.0002; IL-6: 0.0974; IL-12p70: 0.3264; TNF-α: 0.0017; IFN-γ: 0.0049; IL-4: N/A; IL-10: N/A). Post-depletion data is not available for IL-4 and IL-10, as levels of production were frequently below the level of detection (N/D) in both patients and volunteers following depletion (3/3 experiments for IL-4, 2/3 experiments for IL-10).

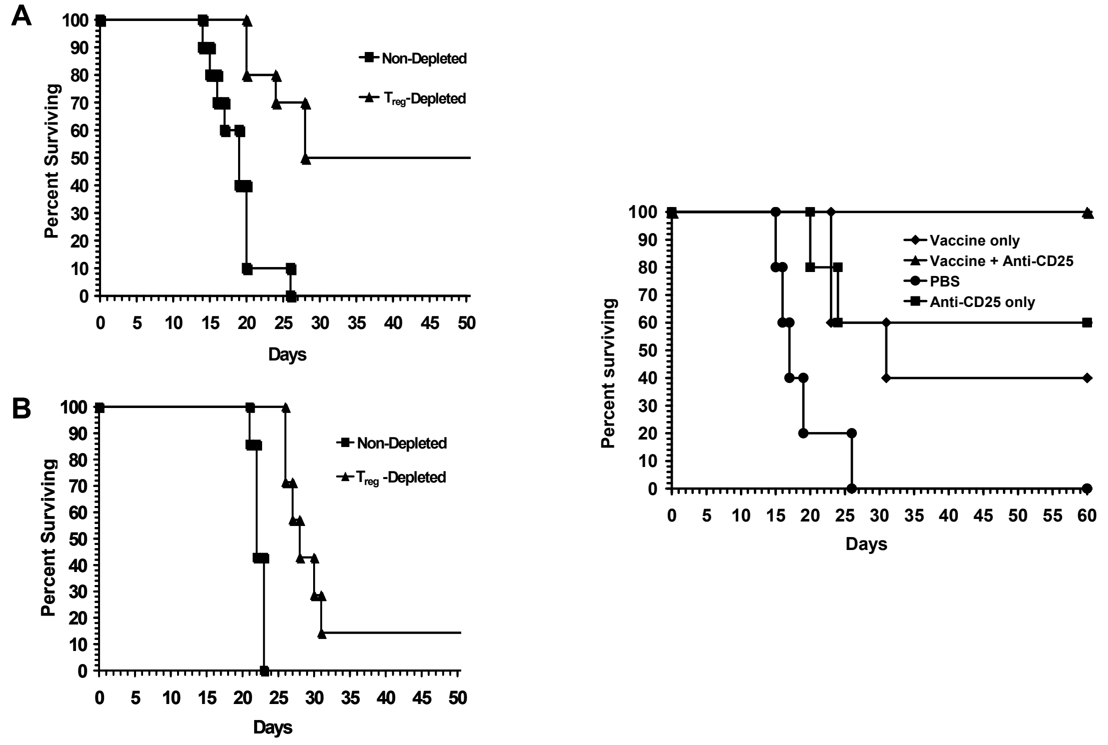

Fig. 9. PC61 *in vivo* extends survival and permits tumor rejection in a murine model of intracranial glioma. *A*. VM/Dk mice (n=10 per group) received i.p. PC61 or isotype control on Day -4. On Day 0, all mice were challenged intracranially with 10,000 syngeneic SMA-560 astrocytoma cells. Anti-CD25 produced 50% long-term survivors with no evidence of tumor. Differences in survival curves are significant (p=0.0002). *B*. Effects of PC61 in a therapeutic model. Treated mice (n=7) received i.p. PC61 three days following tumor implantation, when tumor was established. PC61 significantly extended median survival (p=0.0003) and produced nearly 15% long-term survivors with no evidence of tumor. *C*. Combination of anti-CD25 and DC vaccine produce 100% survival following intracranial tumor challenge. VM/Dk mice (n=5 per group) were adminisitered 0.5 mg PC61 (anti-CD25) or isotype control antibody i.p. on Day -10. On Day -7, mice were vaccinated with 2.5×10^5 DC electroporated with total tumor RNA from the SMA-560 glioma cell line or injected with an equivalent volume of PBS. Injections were delivered s.c. at the base of each ear. On Day 0, all mice were challenged i.c. with 10,000 syngeneic SMA-560 cells. Kaplan-Meier survival data are presented as the percentage of mice surviving in each group. Differences in survival were determined by log rank test. Anti-CD25 (p=0.0198) and DC vaccine (p=0.0153) alone each produced significant survival benefits, but the combination of the two elicited 100% long-term survival (p=0.0018).

3.10 Inhibition of T_{Reg} Function with Anti-CD25-specific Monoclonal Antibodies

We have reported that our murine model of glioma indeed recapitulates MG-induced changes to the human peripheral blood CD4 and T_{Reg} compartments. Specifically, tumor-bearing mice exhibit CD4 lymphopenia, while $CD4^+CD25^+Foxp3^+GITR^+$ T_{Regs} come to represent an increased fraction of the peripheral blood $CD4^+$ T-cells that remain, despite themselves being reduced in number. Extending study to other sites, similar phenomena

are observed in the spleens and cervical lymph nodes (CLN), while the reverse scenario emerges in bone marrow.

We employed this model to investigate the *in vivo* effects of T_{Reg} removal on anti-glioma immune responses. The current doctrine is that effective depletion of T_{Regs} may be achieved simply by systemic administration of anti-CD25 monoclonal antibody (mAb) (Kohm, Carpentier et al. 2002; McHugh and Shevach 2002). Likewise, in peripheral, non-CNS tumor models, the administration of anti-CD25 mAb has been employed in attempts to remove T_{Regs} and has effectively elicited prolonged survival to subcutaneous tumor challenge (Shimizu, Yamazaki et al. 1999; Steitz, Bruck et al. 2001; Sutmuller, van Duivenvoorde et al. 2001). These studies, however, predated our ability to examine Foxp3 expression in T_{Regs} with antibody staining.

Following *in vivo* administration of anti-CD25 mAb (PC61), we discovered that $CD4^{+}Foxp3^{+}GITR^{+}$ cells failed to entirely disappear, despite the present thinking. Instead, they persisted at significant levels in all sites tested. When isolated based on CD4 and GITR expression, however, these cells demonstrated none of the typical suppressive capacities of $CD4^{+}CD25^{+}GITR^{+}$ T_{Regs} *in vitro*. Accordingly, systemic anti-CD25 mAb proved capable of enhancing T-cell proliferation, IFN-γ production and glioma-specific cytotoxic T-lymphocyte (CTL) responses in treated mice. These effects translate to spontaneous tumor rejection in a murine model of established intracranial glioma (Fig. 9). Furthermore, when combined with a dendritic-cell (DC)-based immunization strategy, anti-CD25 mAb elicited glioma rejection in 100% of challenged mice without attendant induction of experimental allergic encephalitis (EAE)(Fecci, Ochiai et al. 2006; Fecci, Sweeney et al. 2006). Systemic anti-CD25 mAb administration therefore appears to counter the suppressive effects of T_{Regs} without comprehensively eliminating the cells *in vivo*. This activity proves permissive for potent antitumor immunity in a murine glioma model that aptly recapitulates tumor-induced changes to the CD4 and T_{Reg} compartments.

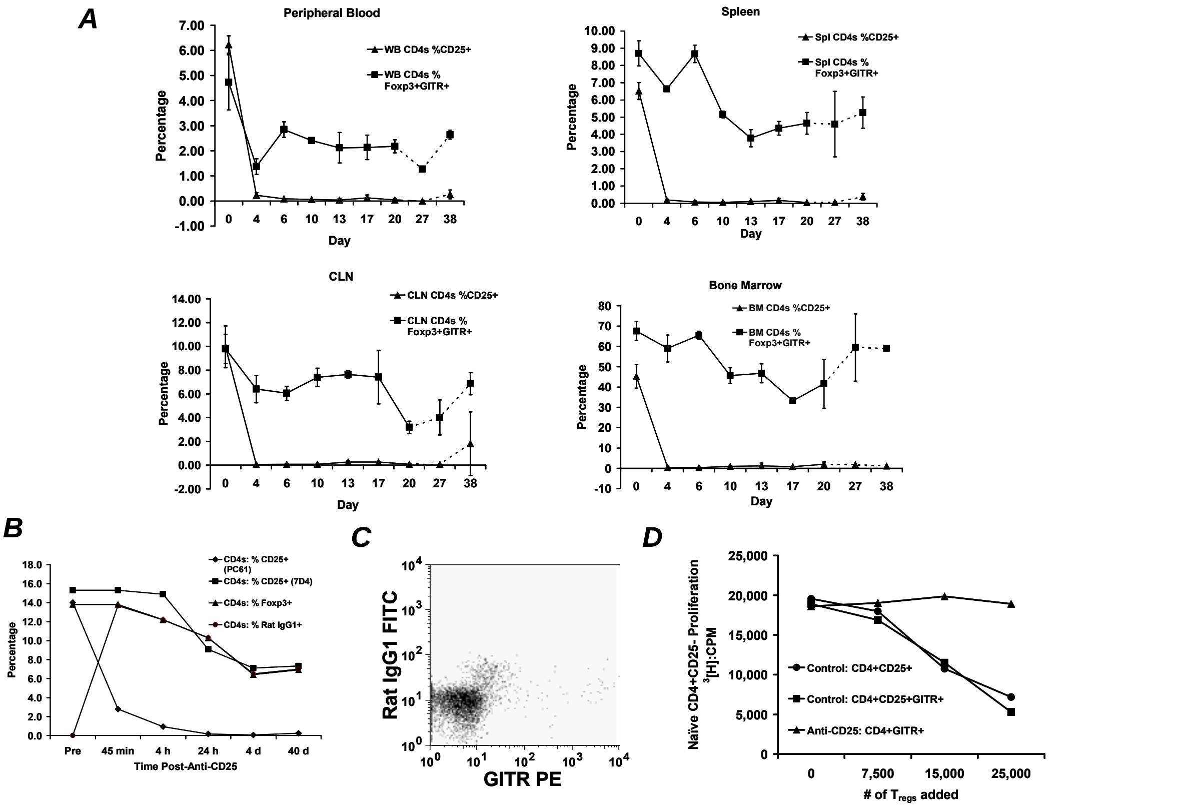

Fig. 10. Systemic anti-CD25 fails to eliminate CD4$^+$Foxp3$^+$GITR$^+$ cells but interferes with their suppressive function. *A*. Relative percentages of CD4$^+$ T-cells in which CD25 (PC61) or Foxp3 and GITR are detectable in the days following *in vivo* anti-CD25 (PC61) administration. Although CD25 becomes rapidly undetectable, CD4$^+$Foxp3$^+$GITR$^+$ cells experience only a slow and moderate decline. B. Time curves of CD25 (PC61), CD25 (7D4), Foxp3, and rat IgG1 detection on CD4$^+$ T-cells in the immediate minutes and hours following in vivo administration of anti-CD25 (PC61). PC61 is a rat IgG1 isotype. *C*. Representative panel demonstrating that following *in vivo* PC61 administration, rat IgG1 is found specifically on the surface of GITR$^+$ cells. Staining was performed 10 days following antibody administration. CLN is depicted. Gating is on CD4$^+$ lymphocytes. *D*. Anti-CD25 blocks suppressive function. Mice were administered 0.5 mg PC61 (anti-CD25) or isotype control antibody (control). After 5 days, spleens and CLN were removed and CD4$^+$ T-cells isolated. Although CD25 was not seen on cells from anti-CD25-treated mice, GITR remained detectable at nearly the same levels and was used to sort CD4$^+$GITR$^-$ and CD4$^+$GITR$^+$ cells from these mice. The ability of these CD4$^+$GITR$^+$ cells to suppress the proliferation of CD4$^+$CD25$^-$ T-cells from control mice was compared that of T_{regs} isolated from control mice on the basis of either CD25 or CD25 and GITR expression. While T_{regs} isolated from control mice suppressed T-cell proliferation in a dose-dependent fashion, CD4$^+$GITR$^+$ cells from anti-CD25 treated mice did not.

3.11 IL-2α-specific Antibodies: PC61 and Daclizumab

PC61, as described above, is a rat IgG1 monoclonal antibody (MAb) that is specific for the mouse IL-2Rα (CD25) and has been used extensively to disrupt the function of $CD25^{+}$ T_{Regs}. Our laboratory was among the first to demonstrate that this dysfunction did not solely result from the depletion of T_{Regs}, but rather that blockade of the IL-2Rα alone could deactivate T_{Reg} suppression (Fig. 10, above))(Fecci, Sweeney et al. 2006; Kohm, McMahon et al. 2006). While others differ on the mechanism of action of PC61(Stephens, Anderton et al. 2006; Zelenay, Demengeot et al. 2006), our own data(Fecci, Sweeney et al. 2006) corroborates that of Kohm *et al.*(Kohm, McMahon et al. 2006) who suggest that IL-2Rα blockade induces shedding of the IL-2Rα and cytokine starvation of the T_{Regs}.

Daclizumab (Zenapax®) is a clinically-approved humanized IgG1 MAb very similar to PC61(Queen, Schneider et al. 1989). It does not mediate direct cell cytotoxicity(Goebel, Stevens et al. 2000), but like PC61 functions as a IL-2Rα antagonist that inhibits IL-2 binding and signaling(Goebel, Stevens et al. 2000; Waldmann 2006). It does not, however, alter expression of the β and γ chains(Tkaczuk, Milford et al. 2001) nor phosphorylation of the βγ signaling domains by IL-15(Goebel, Stevens et al. 2000). It also does not inhibit killing by cytotoxic T-cells(Depper, Leonard et al. 1983). Serum levels of 5-10 μg/mL are necessary to saturate IL-2Rα, and mean peak serum concentrations after the first dose are 21 $\pm$ 14 μg/mL.

Daclizumab has been most frequently used as an immunosuppressant, however, <u>in combination with other broadly active immunosuppressants, such as cyclosporine and corticosteroids</u> that may target the redundancy pathways for effector T-cell development <u>(Abramowicz and Abramowicz 1998; Vincenti, Kirkman et al. 1998)</u>. As an immunosuppressant, it is given every 14 days for a total of 5 doses during which time effector T-cells may be expected to up-regulate IL-2Rα expression and be inhibited. Thus, we believe, and our preclinical animal studies support, that the activity of IL-2Rα blockade is entirely dependent on the context in which it's given. We believe that blocking IL-2Rα signaling during recovery from TMZ-induced lymphopenia will predominantly impact the recovery, function, and survival of T_{Regs} without having negative effects on the recovery, function and survival of tumor-specific T-cells activated through vaccination or through cross-priming at the tumor site where the role of other cytokines will predominate. In support of this belief, *Ontak*® (denileukin difitox), a recombinant molecule consisting of diphtheria toxin conjugated to IL-2 as the targeting moiety, has recently been used to successfully deplete T_{Regs} and enhance antitumor immune responses(Dannull, Su et al. 2005). Results with *denileukin difitox*, however, have been mixed and inconsistent(Attia, Maker et al. 2005; Dannull, Su et al. 2005; Attia, Powell et al. 2006). This may be because the IL-2 moiety is <u>*not*</u> specific for IL-2Rα, but is also capable of targeting the lower affinity IL-2βγ receptors. This might lead to the killing of a broader subset of cells including memory T-cells and NK cells which may have a deleterious effect on the immune response. The IL-2 moiety would also be capable of direct stimulation of the IL-2Rα on T_{Regs} which may lead to partial activation rather than inhibition. Daclizumab, however, although capable of binding to T_{Regs}, effector T-cells, and activated NK cells, all of which may express the IL-2Rα, is less likely to kill

these cells, but only blocks IL-2 signaling through the IL-2Rα which may have no beneficial effect on activated effector cells especially during recovery from TMZ-induced lymphopenia. In this regard, it is interesting that daclizumab has also been used as effective therapy for adult T-cell leukemia associated with HTLV-1 wherein the leukemic cells have a $CD4^{+}CD25^{+}CD45RO^{+}$ phenotype and appear to be the leukemic counterparts of T_{Regs}(Waldmann, Greene et al. 1984; Waldmann, White et al. 1993). Here daclizumab is also thought to work by blocking IL-2-growth signaling, leading to cytokine deprivation and consequent death(Waldmann 2006). This purported mechanism is identical to that which we believe operates for PC61(Fecci, Sweeney et al. 2006; Kohm, McMahon et al. 2006) and which we propose to exploit for the inhibition of T_{Regs} in patients with GBM.

In trials of renal transplantation, the overall incidence of infectious episodes, including viral infections, fungal infections, bacteremia and septicemia, and pneumonia was not higher in the daclizumab–treated patients than the placebo-treated patients. There was, however, a slight increase in wound infections, specifically, with daclizumab. Addition of daclizumab also did not increase the number of post-transplant lymphomas up to three years post-transplant. No difference in abnormal hematologic or chemical laboratory test results were seen between groups treated with placebo or daclizumab with the exception of fasting blood glucose which was likely related to corticosteroids or diabetes. While low titers of anti-idiotype antibodies to daclizumab were detected in adult patients treated with the drug, no antibodies that affected efficacy, safety, serum daclizumab levels or any other clinically relevant parameter examined were detected.

3.12 TMZ-induced Lymphopenia and IL-2Rα Blockade Enhance Immunity

Therapeutic TMZ induces transient, Grade 3 lymphopenia (<500cells/uL) in 70% of patients with GBM after the first cycle (150-200 $mg/m^2/5d$) with nadirs occurring 14-21 days after treatment (n=10). It also enhances the recovery of T_{Regs} (Fig. 11). Thus, we evaluated TMZ for its capacity to induce homeostatic proliferation and to enhance antigen-specific immune responses in a murine model (Fig. 12 and 13). Markedly elevated levels of antigen-specific T-cells could be achieved and maintained in mice receiving antigen-specific vaccination after TMZ-induced lymphopenia when compared to normal hosts. Normal mice exhibited an increase of OVA-specific T cells from 0.49% to 1.90% of circulating CD8+ T cells one week post vaccination which subsequently contracted to 0.40% after two weeks, while TMZ pre-treatment resulted in an expansion from a frequency of 0.40% to 10.64% (absolute count increase from 3.41 cells/uL blood to 42.61 cells/uL) that continued to expand for 4 weeks post-vaccination to 21.18%. OVA-specific T cells in TMZ treated and vaccinated hosts were shown to be Th1-type memory/effector cells ($CD44^{hi}CD43^{lo}IFN\text{-}\gamma^{+}$).

Anti-CD25 treatment (PC61), while having a negative impact on responses in normal mice, led to an even more dramatic enhancement of responses in TMZ treated mice (Fig. 12 and 13). PC61 pre-treatment alone had no effect in normal hosts. In contrast, in mice recovering from TMZ-induced lymphopenia, PC61 treatment given alone or in combination with vaccination significantly enhanced overall and antigen-specific T-cell expansion.

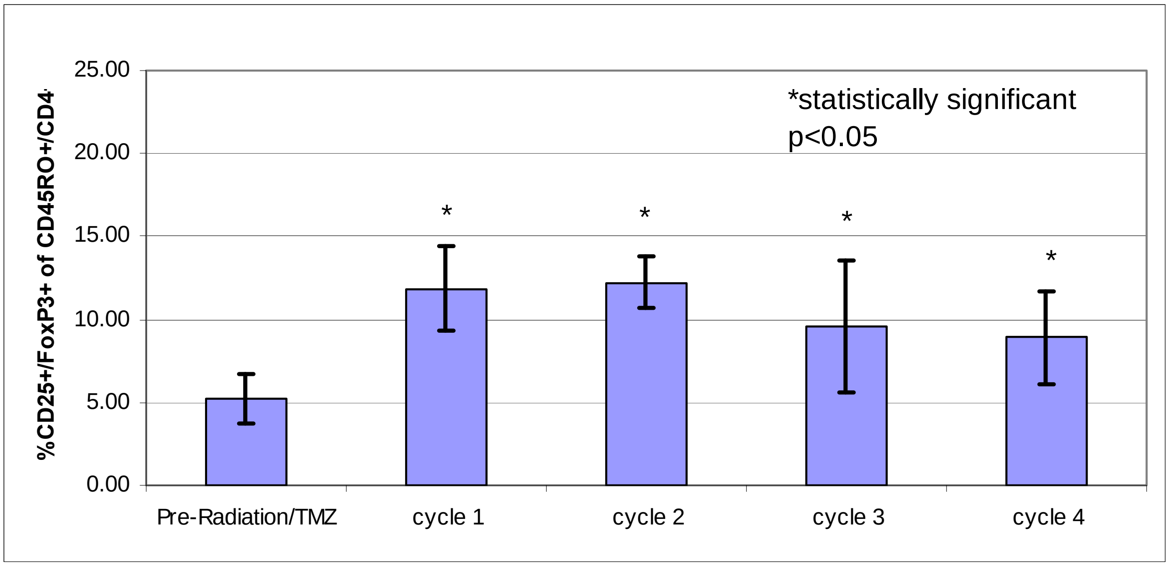

Fig. 11: T_{Reg} (%CD25+/FOXP3+ of CD45RO+/CD4+ T-cells) levels prior to TMZ and after each TMZ cycle. After staining PBMC surface antigens (CD45RO-FITC, CD25-PE, and CD4-PerCpP-Cy5.5, BD #555492, #555432, and #341654 respectively) cells are washed extensively and incubated on ice for 30 minutes in fixation/permeabilization buffer (eBioscience, #5123-43). Cells are then washed in 1X permeabilization buffer (eBioscience, #8333-56), pelleted, and stained with foxp3-APC (e Bioscience, #17-4776-73, clone PCH101). $CD25^{+}foxp3^{+}$ are gated from $CD45RO^{+}CD4^{+}$ lymphocytes. Values are an average of six donor sets representing pre through cycle 4.

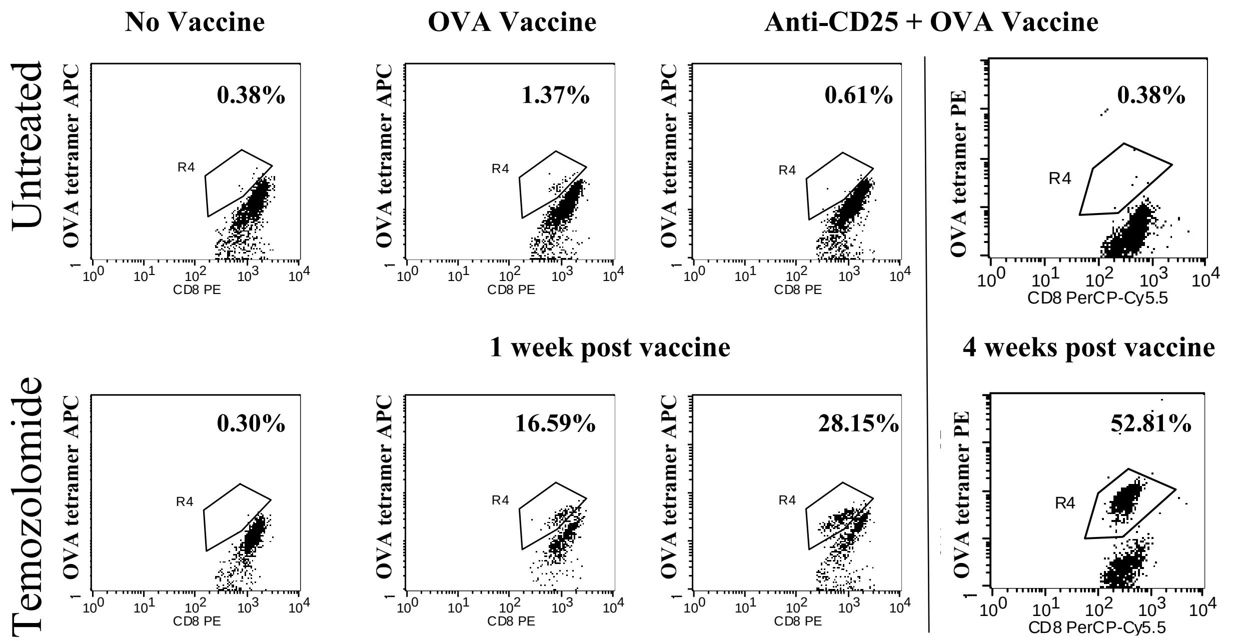

Fig. 12: Enhancement of vaccine responses in mice recovering from TMZ-induced lymphopenia using unarmed anti-CD25 monoclonal antibody treatment. Untreated mice and mice treated with lymphodepletive TMZ (200mg/kg x 1 i.p.) received adoptive lymphocyte transfer (ALT) of naïve splenocytes spiked with OVA-specific transgenic CD8+ T cells (OT-I T cells) 48hrs after TMZ treatment with or without simultaneous anti-CD25 MAb treatment (PC61; 250 □g i.p.). Two days later (4 days after TMZ treatment) some mice received a single intradermal vaccination with a H-$2K^b$ restricted OVA peptide (SINFEKL) (50□g in Complete Freund's Adjuvant) and expansion of OVA-specific T cells was followed weekly in the peripheral blood using an OVA-specific tetramer and CD8 specific antibody. Initial analysis (weeks 1-3) were done using an APC conjugated OVA tetramer and PE conjugated CD8 antibody, however, we found PE conjugated OVA tetramer and PerCP-Cy5.5 conjugated anti-CD8 to given better resolution of antigen-specific T cells (week 4 analysis).

Results above show a dramatic increase in the expansion of OVA-specific T cells in TMZ pretreated hosts compared to untreated mice. Administration of anti-CD25 MAb had a detrimental effect on the expansion of these cells in normal hosts, but in TMZ treated mice anti-CD25 MAb treatment significantly enhanced the vaccine response. The expansion of OVA-specific T cells in normal hosts contracted after two weeks and at 4 weeks the frequency of OVA-specific T cells in the blood was not significantly increased over non-vaccinated animals. However, in mice recovering from TMZ-induced lymphopenia there was a continued expansion of OVA-specific T cells, such that as many as 50% of the circulating CD8+ T cells were OVA-specific. Shown above are the best responders from each group of mice (n=5) and the same mice in anti-CD25+OVA vaccine group at two weeks and four weeks. The mean frequency of OVA-specific T cells for each of these groups was consistent of the data shown above. All responses in TMZ pre-treated hosts were statistically significant (P value < 0.05) when compared to identical group in untreated mice. These differences were also reflected in absolute counts of OVA-specific T cells utilizing FlowCount cytometry beads to enumerate the number of OVA-specific T cells per µl of blood (not shown). The OVA-specific CD8+ T cells induced in these mice were shown to be memory/effector cells at 4 weeks by phenotype ($CD44^{hi}$ x $CD43^{lo}$) and functional analysis (IFN-γ secretion in response to peptide stimulation; not shown). We have obtained similar results using OVA RNA-loaded DC vaccines in temozolomide versus untreated mice and shown similar increases in antigen specific T cells in the lymph nodes, spleen, and bone marrow of TMZ treated animals.

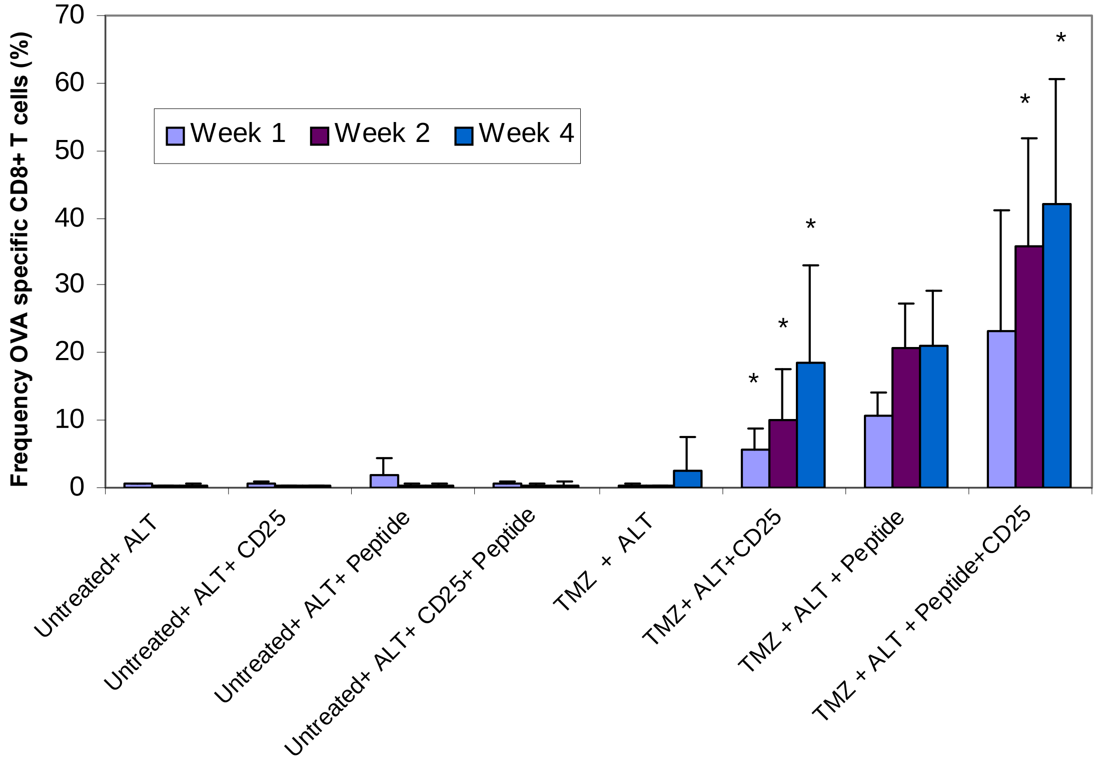

Fig. 13: Enhancement of Immune Responses with TMZ and IL-2Rα Blockade—Mice were lymphodepleted with TMZ (200mg/kg), given normal splenocytes spiked with OVA-specific T-cells (ALT). PC61 (Anti-CD25) was given 2 days later along with a single OVA-specific vaccine. The expansion of OVA-specific T cells in peripheral blood was monitored weekly using a PE conjugated OVA-specific tetramer (**P*< 0.05, Fisher's exact test).

3.13 Daclizumab Eliminates T_{Reg} Function in Monkeys

In collaboration with Dr. Andrew Lackner at the Tulane Primate Center, we have tested daclizumab in a non-human primate model under the direction of Dr. Sempowski (Fig. 20). Initial dose and kinetic studies revealed a delayed but significant depletion in both number and percent of $CD4^+CD25^{hi}$ cells that lasted >4 weeks. (Further characterization of these cells was not possible due to lack of specific reagents in this species. No adverse events were noted.)

3.14 Daclizumab Eliminates Human T_{Reg} and Enhances CD4+ T-cell Response

Although the effects daclizumab may well be different *in vivo* than *in vitro*, we investigated the effects of this IL-2Rα blocking antibody on the survival of human T_{Regs} ($CD4^+CD25^{hi}$) *in vitro* and compared this to denileukin difitox (Ontak). Denileukin difitox failed to kill unsorted $CD25^+$ T_{Regs} *in vitro* possibly due to direct stimulation by the IL-2 moiety. Although others(Dannull, Su et al. 2005) have shown that denileukin difitox does kill $CD25^+$ cells, it is important to note that this was in a selected population of $CD25^+$ cells so the effects of denileukin difitox on other cell populations was not accounted for. This may account for the variable clinical experience with this agent(Attia, Maker et al. 2005). Daclizumab, however, significantly reduced the number of T_{Regs} within 48 hours.

Similarly, we determined if daclizumab would inhibit the expansion and cytokine secretion *in vitro*. Across a large dose range we found a significant dose response relationship between our ability to produce antigen-specific, cytokine-secreting cells and the dose of daclizumab (Fig. 21).

3.15 Rationale for Current Study

We have demonstrated in murine models that the vaccination with a tumor-specific peptide, PEPvIII-KLH (now known as CDX-110), can induce humoral and cellular immune responses and increase survival. Our Phase I and multi-institutional Phase II human studies demonstrate that vaccinations with an EGFRvIII-specific peptide induced T- and B-cell immunity, produce complete radiographic responses in all patients with residual tumor, and universally eliminate EGFRvIII-expressing cells(Heimberger, Hussain et al. 2006; Heimberger, Hussain et al. 2006). Recurrent tumors, however, continue to express wild-type EGFR suggesting that the immune response is specific, but productive intra-molecular cross-priming does not occur. We believe that cross-priming may be hindered by the presence of T_{Regs}.

We have recently shown that T_{Regs} are disproportionately represented within the peripheral blood and tumors of patients with GBM and serve to induce a state of profound, but reversible, immunosuppression(Fecci, Mitchell et al. 2006). T_{Regs} are characterized by constitutive expression of the high affinity IL-2 receptor (IL-2Rα)(CD25) and are absolutely and uniquely dependent on IL-2Rα signaling for their function and survival(Malek 2003; Malek and Bayer 2004; D'Cruz and Klein 2005; Fontenot, Rasmussen et al. 2005; Maloy and Powrie 2005; Fecci, Sweeney et al. 2006). Using our spontaneous murine glioma model, we have already demonstrated that pre-treatment with an antibody that blocks IL-2Rα signaling and functionally inactivates T_{Regs} without inducing autoimmune toxicity(Fecci, Ochiai et al. 2006; Fecci, Sweeney et al. 2006). Established immune responses may also depend on IL-2 signaling of effector T-cells or a novel subset of $CD3^{-}CD56^{bright}$ NK cells(Bielekova, Richert et al. 2004; Li, Lim et al. 2005; Bielekova, Catalfamo et al. 2006), however, such that blocking IL-2Rα signaling during ongoing immune responses may impair effector T-cell responses as well(Williams, Tyznik et al. 2006). After periods of lymphopenia, however, physiologic surges in IL-7(Seddon, Tomlinson et al. 2003; Seddon and Zamoyska 2003) and IL-15(Nishimura, Tagaya et al. 2001) drive homeostatic reconstitution of the effector T-cell compartment and may bypass the need and effects of IL-2 signaling in these cells(Willerford, Chen et al. 1995; Baan, Boelaars-van Haperen et al. 2001).

We have recently shown that TMZ, an alkylating agent that produces a survival benefit in patients with GBM(Stupp, Mason et al. 2005), induces a profound, but transient lymphopenic environment in humans and mice. Our preliminary studies have shown that unarmed CD25-specific antibodies given *in vivo* to mice during recovery from TMZ-induced lymphopenia and in *in vitro* human studies are capable of functionally inactivating T_{Regs} while dramatically enhancing vaccine-induced immune responses.

Although unarmed anti-CD25 antibodies have been used in animal studies for some time now to disrupt the function of regulatory T-cells, our laboratory was amongst the first to demonstrate that this dysfunction did not directly result from the depletion of regulatory T-cells but rather the blockade of the IL-2 receptor (Fecci, Sweeney et al. 2006; Kohm, McMahon et al. 2006). This is consistent with the notion that the only non-redundant function of IL-2 at physiologic levels is the maintenance of regulatory T-cells(D'Cruz and Klein 2005; Fontenot, Rasmussen et al. 2005). This may be especially true within the context of recovery from lymphodepletion when high levels of

homeostatic cytokines such as IL-7 and IL-15 may drive activated T cell division in the absence of IL-2(Baan, Boelaars-van Haperen et al. 2001). This notion is supported by our preclinical work described above which demonstrates that when an unarmed CD25-specific antibody is delivered during the recovery phase from temozolomide-induced lymphopenia, a dramatic up regulation in CD25 expression on all lineages of activated lymphocytes occurs and that vaccine-induced immune responses are significantly enhanced in this context. The antibody used for blockade of the high affinity IL-2 receptor in mice is very similar to the humanized anti-CD25 targeting antibody daclizumab (Zenapax®) which has previously been used in humans as an immunosuppressive agent. We believe, however, that based on our preclinical animal studies, the activity of CD25 blockade is entirely dependent on the context in which it's given and that CD25 blockade during recovery from temozolomide-induced lymphopenia will predominantly impact the recovery and survival of regulatory T-cells without having negative effects on the recovery and survival of activated T-cells. Furthermore, this approach will enhance vaccine-induced immune responses within the context of our clinical trial which targets the tumor-specific EGFRvIII in patients with GBM.

Although others have recently used CD25-targeted toxins to deplete regulatory T-cells (Attia, Maker et al. 2005; Dannull, Su et al. 2005; Attia, Powell et al. 2006), the results of this approach have been variable. This is likely due to the complex pharmacokinetics of this agent and the possibility that the targeting IL-2 moiety actually stimulates regulatory T-cells as well. Although IL-2 in high doses has been used clinically to stimulate T-cell function, its predominant role has recently been shown to be the homeostasis of regulatory T-cells(D'Cruz and Klein 2005; Fontenot, Rasmussen et al. 2005). This is consistent with data from transgene mice which demonstrate no deficits in immunologic function, and in fact the development of autoimmunity, in the genetic absence of the high affinity IL-2 receptor alpha (CD25)(Willerford, Chen et al. 1995)

We hypothesize that daclizumab therapy during the recovery from therapeutic TMZ-induced lymphopenia in patients with newly-diagnosed high grade glioma will inhibit the functional recovery of T_{Regs}, enhance vaccine-induced immune responses, and promote cross-priming without the induction of deleterious autoimmunity.

In this protocol, we will determine if daclizumab inhibits the functional recovery of T_{Regs} after therapeutic TMZ-induced lymphopenia in the context of vaccinating adult patients with newly-diagnosed high grade gliomas using a tumor-specific, EGFRvIII-targeted peptide vaccine.

3.15.1 Dose, Route of Administration, and Dosing Regimen

This trial is designed to replicate the positive Phase II clinical trial utilizing 500 μg PEP3-KLH with approximately 150 μg GM-CSF as an intradermal injection, given every two weeks for three (3) doses and monthly thereafter.

The initial rationale for dose selection for the Phase II PEP3-KLH study was based on administered doses of peptide in previous trials, which had been as low as 1 μg or as high as 3 mg (Miyagi, Imai et al. 2001). The majority of these clinical trials escalated the dose from 100 mcg to 1 mg without dose-related toxicity or clinical activity. As well, in a previous trial, PEP3-KLH had been administered loaded onto dendritic cells at doses ranging from 150 mcg to 500 mcg, with no reported toxicities (unpublished data).

Further refinement of the dosing is not currently feasible. With agents currently approved for oncologic indications, dosing was defined by either acute toxicity or some immediate biologic readout. There are no reliable short term endpoints to use to select the optimal dose for CDX-110. Local reactions and immune responses are highly variable and have not been shown to correlate with any clinical endpoint. Progression-free survival and overall survival require prolonged follow up and large patient numbers for validity. Thus, the submitted phase II/III trial will be based upon the previously tested dose.

Administration routes for previous peptide vaccine trials have been either intramuscular, intradermal or subcutaneous. No definitive randomized clinical trial has thus far been performed to determine whether one route is preferable over another. However, since the intradermal compartment contains a high concentration of dendritic cells it is thought to be a good vaccination route. As with dose, further refinement of the administration route is not currently feasible. There are no reliable short term endpoints to use to select the optimal route for CDX-110 administration. Thus, the submitted phase II/III trial will use the previously tested route to administer the drug.

Finally, dosing intervals in previous trials using peptide vaccines have been between 2 and 4 weeks, with usually two (2) to six (6) administered doses. One study continued the immunization at either the patient or physician's request with a mean of eight (8) doses (Miyagi, Imai et al. 2001). In this protocol, patients will receive the same vaccination regimen used in the previous phase II study: three (3) immunizations 2 weeks apart followed by additional immunization every month until disease progression (Heimberger, Hussain et al. 2006). This vaccine regimen is identical to that used in the ACTIVATE study, and fits conveniently within the most commonly used chemotherapy maintenance regimens for this disease setting (Stupp, Mason et al. 2005), while allowing some intensive vaccination prior to the initiation of chemotherapy.

3.15.2 Standard of Care Regimen

Data generated in a collaborative Phase III study performed by the RTOG and EORTC (Stupp, Mason et al. 2005) clearly suggested that the addition of adjuvant and maintenance TMZ to adjuvant radiation can prolong survival in patients with newly diagnosed GBM. These data served as the basis for FDA approval of TMZ for this indication (Cohen, Johnson et al. 2005). While this regimen has not been universally adopted in the clinic, primarily because the contribution of maintenance TMZ (given for 6 months following concurrent chemoradiation) is not defined, it is certainly recognized as a reasonable standard of care for patients and has been listed as such in the National Comprehensive Cancer Network Clinical Practice Guidelines for CNS cancers Version 1.2006 (http://www.nccn.org/professionals/physician_gls/PDF/cns.pdf).

While it has been traditionally felt that chemotherapy would compromise the cellular elements necessary to generate immune responses, recent data suggest that perhaps chemotherapy can augment immune effects through either preferential elimination of regulatory components, or amplification of antigen exposure following cytotoxic cell damage. The possible synergy of vaccination in combination with conventional chemotherapy has been evaluated (Wheeler, Das et al. 2004; Heimberger, Hussain et al. 2006). In general, it appears that the immune system recovering from a cytotoxic insult may be hyperacute and particularly responsive due both to increased stimulatory

cytokines within the environment and reduced regulatory elements. Specific data have been generated testing the combination of vaccination with temozolomide and have shown that immune responses are not compromised(Heimberger, Sun et al. 2007).

The Stupp regimen of adjuvant and maintenance radiation and temozolomide is generally considered the standard of care for newly diagnosed resectable GBM(Stupp, Mason et al. 2005). It is notable, however, that the Stupp study had relatively poor compliance with the maintenance temozolomide. On the combined chemoradiation arm, only 78% of patients started maintenance temozolomide, and only 47% of patients completed all 6 cycles. The most frequent reason for early termination was disease progression(Stupp, Mason et al. 2005). It is unclear, therefore, what clinical benefit the maintenance regimen added. This question is currently being studied in separate trials.

This protocol will call a slight delay in the initiation of the second cycle of maintenance chemotherapy in all arms. Considering the very limited dose density of maintenance temozolomide actually given in the Stupp study, it is very unlikely that these delays of 1-3 weeks will significantly compromise clinical outcome.

4 STUDY POPULATION

4.1 Inclusion Criteria

4.1.1 Age

The patients must be ≥18 years of age at the time of enrollment.

4.1.2 Disease Status

The patient must have a histopathologic diagnosis of a high grade glioma (WHO Grade III or IV) as confirmed by the study pathologist, Roger McLendon, or his designate. The patient must undergo leukapheresis for immunologic monitoring.

4.1.3 Performance Status

The patient must have a KPS of ≥ 80% and a Curran Group status of I-IV at the time of enrollment.

4.1.4 EGFRvIII

Tumor expression of EGFRvIII by immunohistochemistry (IHC) or polymerase chain reaction (PCR).

4.2 Exclusion Criteria

4.2.1 Disease Status

Patients with radiographic or cytologic evidence of leptomeningeal or multicentric disease will be excluded and replaced if needed.

4.2.2 Prior Therapy

Each patient will have undergone leukapheresis to be eligible for enrollment. This will be followed by standard of care conformal external beam RT with concurrent TMZ. Patients are not permitted to have had any other conventional therapeutic intervention other than steroids, radiation, or TMZ prior to enrollment. Patients who receive previous inguinal lymph node dissection, radiosurgery, brachytherapy, or radiolabeled monoclonal antibodies will be excluded and replaced if needed.

4.2.3 Pregnancy

Females who are pregnant or need to breast feed during the study period will be excluded. A negative β-HCG test will be required within 2 weeks of enrollment.

Patients found to be pregnant will be excluded. Male and female enrolled patients will be advised to practice an effective method of birth control.

4.2.4 Corticosteroids

Patients receiving corticosteroids, with the exception of nasal or inhaled steroid, at a dose above physiologic levels will be excluded. Patients requiring an increase in corticosteroids, with the exception of nasal or inhaled steroid, such that at the time of first vaccination they require a dose above physiologic levels, will be removed from the study and replaced. For the purposes of this study, physiologic dose will be defined as <2 mg of dexamethasone / day. Once vaccinations have been initiated, if patients subsequently require increased steroids, they will still be permitted to remain on the study, but every effort will be made to minimize steroid requirements.

4.2.5 Medical Conditions

Patients with the following conditions will be excluded to avoid confounding the study results:

- Patients with an active infection requiring treatment or an unexplained febrile (>101.5^{o} F) illness.
- Patients with known immunosuppressive disease or known human immunodeficiency virus infection.
- Patients with unstable or severe intercurrent medical conditions such as severe heart or lung disease.
- Patient with prior inguinal lymph node dissection.
- Patients with prior allogeneic solid organ transplantation.

4.2.6 Temozolomide

Patients with demonstrated allergy to TMZ or who are otherwise unable to tolerate TMZ for reasons other than lymphopenia will be excluded and replaced if needed. Patients who are found after enrollment to be unable to tolerate TMZ will not be a candidate for the vaccine despite being previously enrolled and will be removed from the study and replaced.

4.2.7 Daclizumab

Patients who have previously been administered daclizumab or who have had an allergic reaction to daclizumab or one of its components in the past will be excluded.

5 INVESTIGATIONAL PLAN

5.1 Preparation and Administration of Therapeutic Agents

5.1.1 Overview

Twenty patients with newly-diagnosed EGFRvIII+ high grade glioma will undergo leukapheresis for immunologic monitoring. Only 1 dose level of daclizumab (1 mg/kg) will be assessed. The decision to dose escalate in subsequent trials will be dependent on analysis of the safety and immunologic responses obtained in this trial. Patients will be followed until death. This study will be halted if any 2 patients experience a drug-related Grade IV or irreversible Grade III toxicity. After initial leukapheresis, all patients will then receive RT and concurrent TMZ at a standard targeted dose of 75 mg/m2/d. Patients with progressive disease during radiation, dependent on steroid supplements above physiologic levels at time of vaccination, unable to tolerate TMZ or whose PBLs fail to meet release criteria will be replaced, but no more than 40 patients will be enrolled overall. Remaining patients will then receive the initial cycle of TMZ at a targeted dose of 100mg/m2/d for 21 days 3 ± 1 weeks after completing RT and will be independently randomized to receive daclizumab versus saline simultaneous with vaccine #1. An additional cohort of 6 patients (cohort #2) will receive serial administrations of daclizumab during vaccine # 1, vaccine # 3, and during the recovery from each TMZ cycle for the first 3 cycles at the same dose. Vaccines will be given intradermally and divided equally to both inguinal regions. The calculated dosage of daclizumab will be administered in a 50 mL of sterile 0.9% sodium chloride solution via a peripheral or central vein over a 15 minute period. Vaccine #2 and #3 will occur at 2 week intervals. Patients will then be treated monthly with TMZ cycles for a total of 6 cycles after RT. TMZ will be given on days 1-21 of a 28 day cycle. While standard dose 21-day TMZ treatment at 100 mg/m^2/day is recommended, the TMZ regimen may be adjusted at the discretion of the treating neuro-oncologist. Vaccine will always be given on day 21 ± 2 of each 28 day TMZ cycle. Subsequent vaccines will be given on day 21 ± 2 of each of the 5 remaining TMZ cycles. Cohort #2 will receive subsequent vaccinations along with daclizumab (1 mg/Kg) on day 21± 2 for first 3 cycles. Vaccines will then continue monthly until progression. An additional leukapheresis will be required for any patients who have a positive DTH skin test for PEPvIII for immunologic monitoring. Patients will be imaged after vaccine #4 and then bimonthly without receiving any other prescribed antitumor therapy until progression. Blood work for immunologic monitoring will be drawn before vaccine #1 and then monthly after vaccine #3. Blood work for sub-clinical autoimmunity will be drawn before vaccine #1 and every 4 months after vaccine #3. As part of standard care for these patients, upon tumor progression, participants may undergo stereotactic biopsy or resection. As this is not a research procedure consent will be obtained separately. However, if tissue is obtained, it will be used to confirm tumor progression histologically and to assess immunologic cell infiltration and EGFRvIII antigen escape at the tumor site.

5.1.2 Standard Radiation Therapy

External beam RT should begin 4 ± 2 weeks after histopathologic diagnosis of a WHO Grade III or IV high grade glioma and will be given under the direction of the study Radiation Oncologist, John Kirkpatrick, M.D., Ph.D., or his designate, at Duke University or another institution. One treatment of 1.8 Gy will be given daily, 5 days per week, *(33 fractions over less than seven weeks)* for a total of 59.4 Gy. All portals shall be treated during each treatment session. Doses are prescribed at the maximum isodose line encompassing the target volume. Treatment shall be delivered on megavoltage machines of energy ranging from 4 to 18 MV photons. Selection of the appropriate photon energy*(ies)* should be based on optimizing the RT dose distribution within the target volume and minimizing dose to non-target normal tissue. Photon energies > 10 MV should be utilized only in dual energy beam arrangements using at least one beam with energy < 10 MV. Source skin distance for SSD techniques or source axis distance for SAD techniques must be at least 80 cm.

The patient shall be treated in the supine or another appropriate position for the location of the lesion. A head-holding device that is transparent to x-rays and provides adequate immobilization must be utilized at all times during planning and therapy to ensure reproducibility.

The initial target volume shall include the contrast-enhancing lesion and surrounding edema *(if it exists)* demonstrated on pre-operative MRI plus a 2.0-cm margin. If no surrounding edema is present, the initial target volume should include the contrast-enhancing lesion plus a 2.5-cm margin. This primary target volume will be treated to 50.4 Gy in 28 fractions, at 1.8 Gy per fraction.

The boost volume will be based on the post-surgical MRI. After 50.4 Gy, the boost volume will include the contrast-enhancing lesion plus a 1.5-cm margin or, if minimal contrast-enhancing lesion is present at a portion of the resection cavity on MRI, the surgical defect plus a 2.0-cm margin, whichever is greater at that segment of the MRI image. The boost volume will be treated to an additional 9.0 Gy in 5 fractions, 1.8 Gy per fraction. This will bring the total target dose to 59.4 Gy in 33 fractions. All parts of the target volumes are to receive at least 100% but no more than 110% of the dose at the prescription isodose line.

Treatment plans may include opposed lateral fields, a wedge pair of fields, rotation, or multiple-field techniques. MRI-guided 3D treatment planning is necessary to assure accuracy in the selection of field arrangements. Isodose distributions for the primary and boost target volume are required on all patients, including those treated with parallel-opposed fields. A composite plan is required showing the respective target volumes. The inhomogeneity across the target volume shall be kept to a minimum. The maximum dose should be no higher than 10% of the dose at the prescription isodose line. The lens and cervical spine must be shielded from the direct beam at all times. The maximum dose to the optic apparatus (optic chiasm, optic nerves, eyes) must be limited to 54 Gy, and the brain stem to 60 Gy.

5.1.3 *Temozolomide Therapy*

TMZ will be administered concomitant with standard external beam RT under the direction of the study Neuro-Oncologists (listed on title page), or their designates, respectively, at Duke University or another institution at a dose of 75 mg/m^2/d, given 7 days per week from the first day of RT until the last day of RT, but for no longer than 49 days. Dose reduction or alteration during this period due to toxicity will be performed as outlined in Table 1.

Table 1: Temozolomide Dosing Interruption or Discontinuation During Concomitant Radiotherapy		
Toxicity	TMZ Interruption[a]	TMZ Discontinuation
Absolute Neutrophil Count	$\geq$0.5 and <1.0 x 10^9/L	<0.5 x 10^9/L
Platelet Count	$\geq$10 and <100 x 10^9/L	<10 x 10^9/L
Common toxicity criteria (CTC[15]) Non-hematological Toxicity (except for alopecia, nausea, vomiting)	CTC Grade 2	CTC Grade 3 or 4
a: Treatment with concomitant TMZ could be continued when all of the following conditions were met: absolute neutrophil count $\geq$1.0 x 10^9/L; platelet count $\geq$100 x 10^9/L; CTC non- hematological toxicity $\leq$Grade 1 (except for alopecia, nausea, vomiting).		

If interrupted, TMZ may be resumed when re-treatment criteria are met (Table 1). However, if there is a >2 week delay to resume TMZ, the dose will be modified as outlined in Table 2.

Table 2: Dose Modifications of Temozolomide during Radiation Therapy	
**Dose at Toxicity**	**Modified Dose**
75 mg/m2 (during XRT)	60 mg/m2
60 mg/m2 (during XRT)	50 mg/m2

Patients who require a dose modification below 50 mg/m^2 during RT will have TMZ held for the duration of external beam RT, but will be able to resume TMZ following completion of external beam RT and will not be excluded from the study.

Once radiation is complete and following a 3 $\pm$ 1 week break to allow patients meet criteria to resume TMZ (Table 3), patients will receive a total of 6 cycles of TMZ according to the 21-day schedule at a target dose of 100 mg/m^2/d. Standard dose 21-day TMZ treatment at a target dose of 100 mg/m^2/day is recommended, however TMZ regimen may be adjusted at the discretion of the treating neuro-oncologist. Vaccine will always be given on day 21 ± 2 of each 28 day TMZ cycle. A CBC with manual differential (Lab Code 200640) and a T4/T8 ratio (2500202) will be obtained at the end of each cycle and on day of vaccine.

[15] CTC, common toxicity criteria (NCI)

Table 3: Criteria to Resume Temozolomide after Radiation
Absolute Neutrophil Count ≥ 1.0 x 10^9/L
Platelet Count ≥ 100 x 10^9/L
Resolution of CTC Non-hematologic toxicities to Grade 2 or less.

Because of the risk of opportunistic infections in patients receiving TMZ, patients may receive antibiotic prophylaxis at the discretion of the primary Neuro-Oncologist, consisting of inhaled pentamidine or oral levofloxacin. Antiemetic prophylaxis with metoclopramide or a 5-hydroxytryptamine3 antagonist will also be recommended before the initial doses of concomitant TMZ and may be used during the adjuvant five-day course of TMZ.

During TMZ cycles, dose adjustments to TMZ, if needed, will be conducted as outlined below:

Table 4: Temozolomide Dose Reduction or Discontinuation During Maintenance Treatment

Toxicity	Reduce TMZ by 25 mg/m^2/d	Discontinue TMZ
Absolute Neutrophil Count	<1.0 x 10^9/L	See footnote a
Platelet Count	<50 x 10^9/L	See footnote a
CTC Non-hematological Toxicity (except for alopecia, nausea, vomiting)	CTC Grade 3	CTC Grade 4[a]

a: TMZ is to be discontinued if dose reduction to <50 mg/m^2 is required or if the same Grade 3 non-hematological toxicity (except for alopecia, nausea, vomiting) recurs after dose reduction.

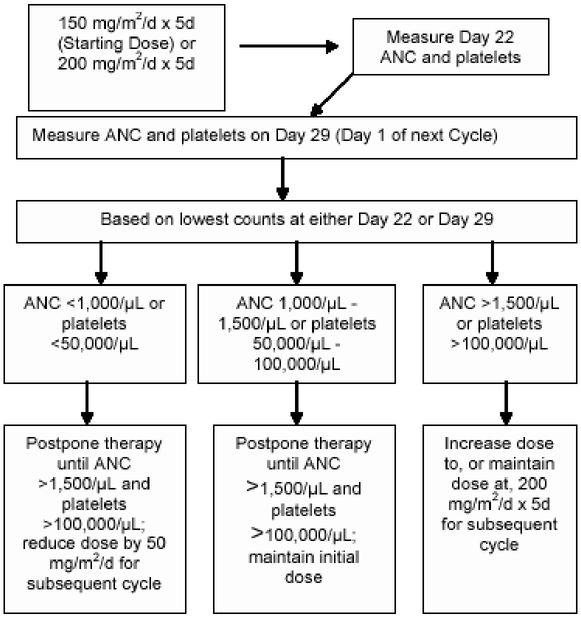

5.1.4 Leukapheresis

Leukaphereses will be performed on each patient enrolled on this protocol. The leukapheresis will be used for immunologic monitoring. All leukaphereses will be between 2 to 4 hours long. It is estimated that 1.5 to 3 times the blood volume (~10-12 L) will be processed during this leukapheresis.

An additional 2-4-hour leukapheresis will be requested from patients with positive immunological responses to the therapy for additional immunologic monitoring studies.

PBMC will be obtained by leukapheresis at the Duke Apheresis Unit (SOP-JHS-HDC-CL-023 "Leukapheresis Collection Procedure") and transported to the Cell Processing facility (SOP-JHS-HDC-CL-011 "Leukapheresis Transport and Receipt Procedure"). For patients without sufficient venous access for leukapheresis a temporary intravenous catheter may be inserted.

5.1.5 Daclizumab

Patients in cohort # 1 will be randomized to receive daclizumab (1 mg/Kg) diluted in saline (FORM-JHS-CP-112 "Administration of Daclizumab"), or saline alone in the same manner. All patients that will receive daclizumab will get it at a 1 mg/Kg dose mixed with 50 mL of sterile 0.9% sodium chloride solution. Daclizumab solution will be administered in a peripheral or central vein over a 15-minute period. When mixing the solution, gently invert the bag in order to avoid foaming: do not shake. Infusion should be administered intravenously within four hours of preparation. If it needs to be held longer, it should be refrigerated between 2° and 8°C (36° to 46°F) for up to 24 hours after which it should be discarded. Medications for the treatment of severe hypersensitivity reactions including anaphylaxis should be available for immediate use. Patients will be monitored for anaphylactic events for 30 minutes.

Patients in cohort # 2 will receive daclizumab (1 mg/Kg) diluted in saline (FORM-JHS-CP-112 "Administration of Daclizumab") with vaccines # 1, vaccine # 3, and during the recovery from each TMZ cycle for first 3 cycles at the same dose.

5.2 Treatment Procedures

5.2.1 Vaccination

After initial leukapheresis, all patients will then receive RT and concurrent TMZ at a standard targeted dose of 75 mg/m^2/d. Patients with progressive disease during radiation, dependent on steroid supplements above physiologic levels at time of vaccination, unable to tolerate TMZ or whose PBLs fail to meet release criteria will be replaced, but no more than 40 patients will be enrolled overall. Remaining patients will then receive the initial cycle of TMZ at a targeted dose of 100mg/m^2/d for 21 days 3 ± 1 weeks after completing RT and will be independently randomized to receive daclizumab versus saline simultaneous with vaccine #1. An additional cohort of 6 patients (cohort #2) will receive serial administrations of daclizumab during vaccine # 1, vaccine # 3, and during the recovery from each TMZ cycle for first 3 cycles at the same dose. Vaccine #1 will be given on day 21 ± 2 days after the start of the 100mg/m^2/d dose of TMZ. Each immunization will be divided equally to both inguinal regions. A total volume of 200 µL

per side will be delivered intradermally (SOP-JHS-HDC-CL-012 "Administration of Vaccine"). Details of the procedure will be recorded on the appropriate form (FORM-JHS-HDC-CL-012). Injection will be performed using a 1.5 inch 25 gauge needle. Due to the potential for particulates, CDX-110 final drug product should be filtered through a 5 micron filter needle (e.g., Becton Dickenson part no. 305200) prior to administration. The CDX-110/GM-CSF admixture should be injected as soon as possible after mixing. Prior to administration, 0.35 mL of GM-CSF will be withdrawn, under aseptic conditions, from the stock vial of Leukine® (500 mcg/mL) using a hypodermic needle and syringe. This 0.35 mL volume, containing 175 mcg GM-CSF, will be added aseptically to the vial containing 0.6 mL CDX-110, and mixed by gentle inversion of the vial (DO NOT SHAKE). After mixing, 0.8 mL of the CDX-110/GM-CSF admixture will be gently withdrawn (avoiding foaming and excess shearing) *using the provided filtration needle*, under aseptic conditions. The filtration needle should be removed and replaced with an appropriate needle for intradermal injection *before* the injection is administered to the patient. This 0.8 mL dose will contain approximately 500 mcg CDX-110 and 150 mcg GMCSF. Any remaining GM-CSF may be stored for up to 20 days at 2-8°C once the vial has been entered, and utilized for subsequent study drug administrations. Any remaining solution should be discarded after 20 days.

Patients will be monitored in the clinic for thirty minutes to one hour post-immunization for the development of any adverse effects. The immunization procedures will be supervised by a nurse or physician that has completed an Advanced Cardiac Life Support (ACLS[16]) course. A cardiac resuscitation cart will be available in the immediate vicinity when performing these immunizations in case of severe allergic reactions. The initial three vaccines will be given each 2 $\pm$ 1 week apart.

Patients will receive TMZ which will be delivered every 4 weeks. Therefore, in addition to the concurrent TMZ (75 mg/m^2/d) given with radiation, a total of 6 addition cycles of TMZ (100 mg/m^2/days 1-21) will be given. While standard dose 21-day TMZ treatment at a target dose of 100 mg/m^2/day is recommended, TMZ regimen may be adjusted at the discretion of the treating neuro-oncologist. Vaccine will always be given on day 21 ± 2 of each 28 day TMZ cycle. Subsequent vaccines will be given on day 21 ± 2 of each of the five remaining TMZ cycles. Cohort #2 will receive subsequent vaccinations along with daclizumab (1 mg/Kg) on day 21± 2 for first 3 cycles. Vaccines will then continue monthly until progression. An additional leukapheresis will be required for any patients who have a positive DTH skin test for PEPvIII for immunologic monitoring.

If the TMZ cycles are completed without progression, monitoring will continue until clinical or radiographic progression without any additional prescribed antitumor therapy. During that time period, patients will be monitored clinically with routine physical and neurologic examinations and MMSE testing at every visit and with a contrasted-enhanced CT or MRI every 8 $\pm$ 4 weeks. Peripheral blood will be obtained as outlined in the study flow sheet below for sub-clinical autoimmunity and immunologic monitoring.

5.2.2 Daclizumab

All patients that will receive daclizumab will get it at a 1 mg/Kg dose mixed with 50 mL of sterile 0.9% sodium chloride solution with vaccine #1. A second cohort of 6

---

[16] ACLS, advanced cardiac life support

patients (cohort #2) will receive serial administrations of daclizumab at vaccine # 1, # 3, and during the recovery from each TMZ cycle for first 3 cycles at the same dose.

5.2.3 DTH Placement and Biopsy Procedure

After completing RT and along with vaccination #1 patients will undergo baseline DTH testing with standard recall antigens. The vaccine site will also be assessed for a DTH-type response. Recall antigens (Tetanus toxoid, *Candida*, and *Trichophyton*) will be prepared and administered according to SOP-JHS-HDC-CL-014 “Delayed type hypersensitivity testing and biopsy procedure”. Beginning at the time of the fourth vaccination, the DTH tests for recall antigens will be repeated. An additional leukapheresis will be required for any patients who have a positive DTH skin test for PEPvIII for immunologic monitoring. The patient will be monitored after the administration of all skin tests for of the development of severe allergic reactions. A physician or nurse who has completed an ACLS course will supervise the skin tests, and a cardiac resuscitation cart will be available in the immediate vicinity when performing these procedures. A 5 mm diameter of erythema or induration will be considered positive. Patients with positive reactions at the site of human PEPvIII-KLH will be biopsied under local anesthesia for evaluation of the infiltrate (SOP-JHS-014 “Delayed Type Hypersensitivity Testing and Biopsy Procedure). The biopsy will be a standard 4 mm “punch” biopsy routinely used in the Dermatology Clinic.

5.3 Safety, Toxicity, and Adverse Events

5.3.1 Dose-limiting Toxicity

DLTs will be tracked using the NCI CTC (Version 3.0) and our own Clinical Neurologic Change (CNC[17]) criteria. CNC will be defined as a decrease in the Mini-mental Status Examination (MMSE[18]) of $\geq$ 6 points, a decrease in KPS of $\geq$30 points, or the development of a new focal neurologic deficit. A DLT will be defined as *any* irreversible CNC, irreversible Grade 3 toxicity, any Grade 4 toxicity, or any life-threatening event not attributable to a concomitant medication, co-morbid event, or disease progression that has been documented histologically. If biopsy cannot be obtained, any new radiographic or clinical changes, as defined above, will be considered a DLT. Also as defined above, any life-threatening event, even if considered a Grade 3 toxicity, will still be considered a DLT even if reversible. CNC and Grade 3 toxicities will be declared irreversible if they cannot be reversed within 2 months of their onset.

5.3.2 Management of Toxicities

Any Grade 4 toxicity or any life-threatening event not attributable to a concomitant medication, co-morbid event, or disease progression, even if reversible, will be considered a DLT. To attribute the Grade 4 toxicity or life-threatening event to tumor progression, tumor progression will need to be confirmed histologically. If biopsy cannot be obtained, any new radiographic or clinical changes that produce a Grade 4 or life-

[17] CNC, Clinical Neurologic Change
[18] MMSE, Mini-mental Status Examination

threatening toxicity will be considered at DLT. Life-threatening events, as described above, even if considered a Grade 3 toxicity, will still be considered a DLT even if reversible and in such cases no further vaccinations will be given..

If a CNC or Grade 3 NCI CTC toxicity is seen that is not attributable to a concomitant medication, co-morbid event, or disease progression that has been documented radiographically or clinically, the next immunization for that patient will be withheld for up to 2 months or until the NCI CTC toxicity improves to a Grade 2 or until the KPS score returns to within 10 points of baseline or the MMSE returns to within 2 points of baseline. CNC and Grade 3 toxicities will be declared irreversible if they cannot be reversed within 2 months of their onset. If the event cannot be reversed within 2 months of its onset, a DLT will be declared for that patient and no further vaccinations or study-related procedures will be performed. If the event is reversed, but a Grade 3 NCI CTC toxicity or a CNC is again seen with subsequent vaccinations, all further vaccinations and study-related procedures will be withheld and a DLT will be declared. Medical therapy may be used to reverse any toxicity if necessary, but any episode of a CNC requiring surgical intervention will still be considered a DLT even if reversible by surgery.

Patients will be evaluated for toxicity as a whole. If no patients in the first cohort of 3 patients experience a toxicity within 2 weeks another cohort of three patients will be enrolled. If any 2 patients develop a DLT, no further enrollment will be permitted and the study will be closed. Because we have previously safely treated many patients with the vaccine alone, toxicity in each arm will be evaluated independently.

5.4 Adverse Event Reporting and Documentation

An "Adverse Event" will be defined as any adverse change from the subject's pre-treatment baseline condition, including any clinical or laboratory test abnormality that occurs during the course of research after treatment has started. Adverse events will be categorized and graded in accordance with the NCI CTC (Version 3.0).

A "Serious Adverse Event" will be defined as an undesirable sign, symptom or medical condition which 1) is fatal or life threatening; 2) requires inpatient hospitalization or a prolongation of existing hospitalization; 3) results in persistent or significant disability/incapacity; 4) constitutes a congenital anomaly or a birth defect and/or; 5) medically significant such that it may jeopardize the subject, and may require medical or surgical intervention to prevent one of the outcomes listed above.

A summary of all adverse events (not just those considered related to the vaccine) will be kept which will categorize the event by organ system, relationship to treatment, its grade of severity, and resolution. The PI will periodically review the collective adverse events with the intention of identifying any trends or patterns in toxicity. If any such trends are identified, depending on their severity and frequency, a protocol amendment will be considered.

All adverse events which are serious and unexpected should be reported immediately to Dr. John H. Sampson (Pager: 919-970-5909) or his designee (919-684-8111) and to the FDA. Fatal or life-threatening, unexpected adverse events will be reported to the FDA by telephone, facsimile, or in writing as soon as possible, but no later than 7 calendar days after first knowledge by the sponsor followed by as complete a report as possible within 8 additional calendar days. Serious, unexpected adverse events that are

not fatal or life-threatening will be reported to the FDA by telephone, facsimile, or in writing as soon as possible, but no later than 15 calendar days after first knowledge by the sponsor.

All adverse events that are considered serious, unanticipated, and related or possibly related to the research (as defined by 21CRF312.32[a]) will be reported to the Duke University Medical Center IRB using the appropriate SAE report form. At the time of the annual progress report to the Duke University Medical Center IRB, a summary of the overall toxicity experience will be provided.

6 DATA AND SPECIMEN COLLECTION

6.1 Pretreatment Evaluation

6.1.1 Baseline and Eligibility

To be enrolled, patients must undergo leukapheresis. Clinical and laboratory evaluations will be obtained within 2 weeks of enrollment for the purposes of meeting Inclusion and Exclusion Criteria as outlined in the Study Flow Sheet.

Pathology slides consisting of 1 H&E and 19 unstained slides, from all prior resections or biopsies, prepared on Fischer Plus glass or Histostix coated slides when available will be mailed to:

Duane A Mitchell, M.D., Ph.D.
220 Sands Building
Research Drive
Duke University Medical Center
Durham, NC 27710

Blood samples will also be sent for the following study related tests: T4/T8 ratio (Lab Code 2500202) and, ABC (Lab Code 2010200) with manual differential (Lab Code 2000640).

Initial clinical evaluations will also include a complete physical, ophthalmologic and neurologic examination with KPS rating, MMSE testing, and Curran rating.

A baseline and study eligibility MRI (with and without gadolinium enhancement) of the brain using standard 5 mm slices with 2.5 mm spacing for comparison to subsequent MRI images will also be obtained.

Patients will also be assessed before vaccine # 1 and every 4 months after vaccine # 3 by a panel of clinical laboratory analyses to screen for the development of autoimmunity. The most common manifestations of autoimmunity seen in related trials(Phan, Yang et al. 2003; Attia, Phan et al. 2005; Blansfield, Beck et al. 2005; Jaber, Cowen et al. 2006) have included enterocolitis, dermatitis, uveitis, hepatitis, and hypophysitis. Laboratory evaluations to detect subclinical disease will be obtained as follows: serum thyroglobulin Ab(Lab Code 1802105), rheumatoid factor (Lab Code 5560195), antinuclear Ab (Lab Code 5560214) (Homma, Sagawa et al. 2006); human anti-human (anti idiotypic) Ab (Sampson Lab), erythrocyte sedimentation rate (Lab Code 2000860), thyroid-stimulating hormone (Lab Code 1802085), ACTH (Lab Code 1800020), cortisol (Lab Code1802010) , free T4 (Lab Code 1802081), ALT, and AST (Lab Code 1004000). Finally, if cerebrospinal fluid becomes available, it will be checked for oligoclonal banding (Lab Code 1543440).

6.2 Data and Specimens to be Accessioned

6.2.1 During Temozolomide

Patients will have a T4/T8 ratio (Lab Code 2500202) and ABC with manual differential (Lab Code 200640) approximately weekly during TMZ administration during radiation. During cycles of TMZ, the laboratory tests will be obtained prior to each next cycle and at the time of vaccination (Day 21 ± 2).

6.2.2 Prior to Initial Leukapheresis

After patients have been enrolled, and before leukapheresis, a ProTRAK form will be submitted to the Cancer Center Protocol office. All enrolled patients will then undergo a leukapheresis for baseline immunological monitoring. Prior to leukapheresis, patients will have blood samples taken for the following tests as required by the Duke Apheresis Center: OP8 (Lab Code 1004500) and β-HCG (Lab Code 1800440). Total estimated blood volume required for these evaluations is 10 cc. For patients without sufficient venous access for leukapheresis, a temporary intravenous catheter may be inserted. To prevent the development of hypocalcemia from the citrate used for leukapheresis, all patients will be given oral Tums, 2 tablets, b.i.d for 2 days prior to the leukapheresis procedure. Patients who have lowered levels of calcium will be treated per Apheresis lab standard protocols under the direction of Dr. DeCastro, M.D. All leukaphereses will be 2 to 4-hours long, and it is estimated that 1.5 to 3 times the blood volume (~10-12 L) will be processed during all leukaphereses.

6.2.3 Prior to Immunizations

Prior to the first immunization, DTH skin tests(Tjoa, Simmons et al. 1998; Lodge, Jones et al. 2000) will be administered and assessed as outlined in Standard Operating Procedure (SOP-JHS-HDC-CL-014 "Delayed-Type Hypersensitivity Test Procedure). Responses will be assessed and recorded on the appropriate form (FORM-JHS-HDC-CL-014C "Delayed-Type Hypersensitivity Test Form"). An additional leukapheresis will be required for any patients who have a positive DTH skin test for PEPvIII for immunologic monitoring.

6.2.4 During and After Immunizations

- Clinical evaluations with each vaccine or leukapheresis will include a general physical, neurologic, and ophthalmologic examination, KPS, and MMSE.
- Radiographic evaluations consisting of a contrast enhanced brain CT or MRI scan will be obtained every 8 ± 4 weeks.
- Blood will be obtained for T4/T8 ratio (Lab Code 2500202), ABC (Lab Code 2010200) with manual differential (Lab Code 2000640), serum for PEPvIII-KLH ELISA (Sampson Lab), and 10 yellow ACD (9 mL), 3 red (9 mL), and 1 purple (3 mL) tubes for Immunologic Monitoring ***prior*** to vaccination #1 and with each vaccination after vaccination #3. Blood will also be obtained for

sub-clinical autoimmunity prior to vaccine # 1 and every 4 months after vaccine # 3. Estimated volume of blood for these tests is 120 cc.

- During TMZ cycles, ABC (Lab Code 2010200) with manual differential (Lab Code 2000640) will be monitored at the time of vaccination (day 21 ± 2) and before the next TMZ dosing of each 4 week cycle.
- DTH testing will occur with the fourth vaccination (SOP-JHS-HDC-CL-014 "Delayed Type Hypersensitivity Testing Procedure"). Results will be read and recorded on the appropriate form (FORM-JHS-HDC-CL-014C "Delayed Type Hypersensitivity Testing Form") 48 ± 24 hours later. An additional leukapheresis will be required for any patients who have a positive DTH skin test for PEPvIII for immunologic monitoring.

7 CLINICAL RESPONSE EVALUATIONS AND STATISTICS

7.1 Radiographic Response and Progression Criteria for Patients with Residual Disease

Residual disease will be defined as contiguous contrast enhancement > 1cm x 1 cm in each of two perpendicular planes. For residual disease present at the time of the last MRI prior to initiating vaccine treatment, radiographic response will be evaluated based on the NCI-endorsed, World Health Organization RECIST criteria and using a modified version of the MacDonald criteria(Macdonald, Cascino et al. 1990; Galanis, Buckner et al. 2006) which is outlined below.

7.1.1 Complete Response

Complete disappearance of enhancing tumor on consecutive CT or MRI images at least 1 month apart, and neurologically stable or improved and at the same or a lower steroid dose. To be assigned a status of "Complete Response" as a best overall response, changes in tumor measurements must be confirmed by repeat assessments that should be performed no less than 4 weeks after the criteria for response is first met.

7.1.2 Partial Response

At least a 30% reduction in longest diameter on an axial image of any enhancing tumor on consecutive CT or MRI images at least 1 month apart, and neurologically stable or improved and at the same or a lower steroid dose. To be assigned a status of "Partial Response" as a best overall response, changes in tumor measurements must be confirmed by repeat assessments that should be performed no less than 4 weeks after the criteria for response is first met taking as a reference the first post-radiation scan.

7.1.3 Stable Disease

Insufficient change to qualify for Partial Response or Progressive disease.

7.1.4 Progressive Disease

At least a 25% increase in the longest diameter on an axial image of any enhancing tumor that is > 1 cm in each of 2 perpendicular planes on consecutive CT or MRI images or the appearance of a new radiographically demonstrable lesion that measures 1cm or greater in each of two perpendicular axial planes taking as a reference the first post-operative or first post-radiation scan whichever is most recent.

7.1.5 Not Assessable

Progression has not been documented and one or more sites have not been assessed.

7.2 Special Circumstances

- **If a scan is suspicious but not definitive for progressive disease (including indeterminate lesion size or enhancement that is not clearly disease progression) in the absence of clinical progression, an investigator may elect to confirm progression through alternate means, including PET scan, biopsy, repeat MRI at 4 weeks, symptomatic follow up or other modalities.**
  - Protocol treatment will continue until the investigator considers progression documented.
- **Disease that was measurable at baseline and appeared to be one solid mass splits to become 2 or more smaller lesions**
  - note that the lesion has split, to identify the separate lesions as unique and to record the measurements of each.
  - appearance of these individual split lesions will not automate a response assignment of PD; they will contribute to a determination of progression only if the sum of all lesions meets the criteria for PD.
- **2 or more lesions which were distinctly separate at baseline become confluent at subsequent visits**
  - note the occurrence and capture the required measurement(s) of the confluent mass.
- **A required region of a baseline scan may be unavailable or technically inadequate, and 1 or more lesions are detected on a subsequent scans.**
  - Such lesions are identified as additional evaluable lesions that do not contribute to determination of that visit's response.
  - If at a successive time point disease progression is noted, the overall response per time point will be PD.
  - For those areas inadequate at baseline, the first scan available will become the reference scan by which later scans are compared for evaluable progression.
- **1 or more lesions are detected in regions unavailable or inadequate at baseline that were also not required**
  - regarded as new lesions, resulting in an overall response of PD for that time point.
- **Regions are not available or adequate at post-baseline visit assessments that showed disease at baseline**
  - an overall response of NA will be assigned to that visit unless other available data shows progression.

Overall Response	Measurable Lesions	Evaluable Lesions	New Lesions	Overall neurological status	Steroid Use	Additional notes
**PD**	PD	PD	Yes	Unequivocal worsening	Stable or increased use	PD by any single component (not steroids by criteria) no sooner than 1 month after the first course of treatment
**SD**	***All other combinations, but please note the following particular combinations:***					
	CR, PR or SD	CR or SD	No	NA	Off steroids, stable dose	Note that a lack of steroid or neurological information could prevent CR or PR from being recorded
	CR, PR or SD	CR or SD	No	improved, unchanged, possible worsening	NA	Note that a lack of steroid or neurological information could prevent CR or PR from being recorded
	NA	NA	NA	improved, unchanged, possible worsening	Off steroids, stable use	
	CR, PR or SD	CR or SD	No	improved, unchanged, possible worsening	Off steroids, stable dose	A CR or PR overall response assignment is not possible with a neurological grading of -1.
**PR**	PR or CR	CR or SD	No	unchanged or improved	Off steroids, stable use	All conditions need to apply and CR criteria do not apply.
**CR**	CR	CR	No	unchanged or improved	Off steroids	All conditions need to apply.
**NA**	NA	NA	NA	NA	Off steroids, stable dose	NA applies when NA is true of both radiology and neurological status.

7.3 Time to Progression (TTP)

Patients potentially eligible for this study will be followed from the time of their initial presentation, if possible. The TTP will be defined radiographically or clinically, as delineated above; or alternatively defined by a significant overall change in clinical status as assessed by the study neuro-oncologist, Henry S. Friedman, M.D. or his designate. In the absence of radiographic or histologic evidence of progression, clinical progression will be defined as a change in KPS of >30 points or a change in MMSE of >6 points that is not reversible with medical therapy within 2 months. Clinical progression in this context will constitute a DLT if not attributable to a concomitant medication, co-morbid event, or disease progression that has been documented radiographically or clinically.

An intent-to-treat analysis will be conducted to compare the TTP experience of patients identified to receive vaccine treatment upon completion of concurrent external beam radiation and temozolomide assuming there was no intervening disease progression to that experienced by a historical cohort of patients administered the same external beam radiation and temozolomide treatments(Stupp, Mason et al. 2005). Of particular interest is whether the TTP experience of the two groups begins to diverge at the time that vaccine is administered 3-4 months after patient enrollment.

7.4 Survival

Survival data in the context of this Phase I/II trial will be interpreted with great care. Still there are a few studies in the literature that may be able to be compared to our study to some degree given that they have similar patient populations. For example, the carmustine polymer study(Westphal, Hilt et al. 2003) and the TMZ study(Stupp, Mason et al. 2005), both have a set of very similar inclusion and eligibility criteria.

In addition, the authors of this study are also aware of the benefit of recursive partitioning analysis of prognostic factors in the comparison of different treatment strategies for patients with MG(Curran, Scott et al. 1993; Lamborn, Chang et al. 2004). Comparison with these studies will allow us to compare the results of the current clinical trial to previously published trials of patients with similar prognostic features.

An intent-to-treat analysis will be conducted to compare the survival experience of patients identified to receive vaccine treatment upon completion of concurrent external beam radiation and temozolomide assuming there was no intervening disease progression to that experienced by a historical cohort of patients administered the same external beam radiation and temozolomide treatments(Stupp, Mason et al. 2005). Of particular interest is whether the survival experience of the two groups begins to diverge at the time that vaccine is administered 3-4 months after patient enrollment.

8 IMMUNOLOGIC MONITORING AND STATISTICAL ANALYSIS

Immunological response evaluations for baseline values will be conducted on the pre-vaccine leukapheresis sample. An additional leukapheresis will be required for any patients who have a positive DTH skin test for PEPvIII for immunologic monitoring. Immunologic response evaluations will also be performed from peripheral blood obtained. A comparison of pre-therapy lymphocyte functions to those at intervals after each immunization and subsequently will be made. These tests may provide evidence for the inhibition of T_{Reg} function or a change in the quantity or quality of immune responses following CDX-110 immunization and will play an important role in the design of future CDX-110-based clinical trials.

Clinical and immunologic endpoints will be analyzed in all patients and statistical considerations described below are powered based on enrollment of 20 patients. However, an analysis of patients stratified at enrollment based on complete resection or residual disease will be performed for hypothesis generation on the role of tumor burden on clinical and immunologic responses to PEPvIII vaccination and daclizumab.

8.1.1 Overview of Statistical Analysis

Our laboratory and the HVTN Immunologic Monitoring Reference Laboratory, headed by Kent Weinhold also at Duke University, has devoted considerable effort to co-developing and co-validating the immunologic assays outlined below for monitoring T_{Reg} function, antigen-specific responses in CD8+ or CD4+ T-cells, and NK cell functional activity in cancer patients. The reproducibility of these assays in our hands ensures that they can be reliably used for monitoring of changes after vaccination with and without daclizumab. Power calculations for this study are based on our primary endpoint which is functional suppressive capacity of CD4+CD25+CD127- T_{Regs}. For these calculations, we assumed that measurements made in each treatment group at a given phytohemagglutinin (PHA) level would be normally distributed with standard deviation of σ. Based on our experience with these assays within this patient population(Fecci, Mitchell et al. 2006), we estimate that with a sample size of 20 randomized patients we will have 80% power to detect a difference of 1.41 σ at the 0.017 significance level (one-tailed). A 0.017 level of significance is used to adjust (Bonferroni correction) for the use of 3 non-zero PHA doses (0.1, 1, 10 µg/well) in the proliferation assay. Thus, with only 20 patients, we will have sufficient power to detect treatment effects much more subtle than the ones observed in our pre-clinical studies(Fecci, Sweeney et al. 2006) and effects that we believe would be clinically significant. However, because the magnitude and variability of the effects of daclizumab on T_{Regs} remain unknown in humans, once human data become available from these studies, power analyses will be recalculated and patient numbers adjusted if needed. If daclizumab treatment does not significantly inhibit the functional recovery of T_{Regs} (primary endpoint), we would not be interested in the further development of this drug as

a vaccine adjuvant. Therefore, a one-sided hypothesis test is utilized. Power calculations are not provided for subsequent analyses as the number of patients in this trial will be determined by the primary endpoint, and the remainder of the analyses is exploratory and hypothesis generating only. With 10 patients in each arm, however, we will have 80% power to detect difference >1.325σ between groups fopr each assay at α=0.05.

8.2 T_{Reg} Functional Assays

The functional capacity of $CD4^+CD25^+CD127^-$ T_{Regs} will be assessed, as described by us previously(Fecci, Mitchell et al. 2006), and comparisons will be made between groups that randomly received saline or daclizumab. Briefly, leukapheresis samples are diluted, underlayered with Ficoll (Histopaque 1077, Sigma), and spun for 25 mins. Interphases are collected, washed extensively, and subjected to a 2 hour adherence step to remove monocytes. A $CD4^+$ T-Cell Isolation Kit II (Miltenyi) is used to isolate untouched $CD4^+$ cells according to the manufacturer's instructions using a biotinylated antibody cocktail specific for non-$CD4^+$ (CD8, CD14, CD16, CD19, CD36, CD56, CD123, TCRγδ and Glycophorin A) and anti-biotin microbeads. CD127-biotin (BD, #558633) is also added to the isolation cocktail to deplete $CD127^+$ cells. CD25+ cells are further isolated using Miltenyi CD25-beads (#130-090-445). Alternatively, unlabeled fractions ($CD4^+CD127^-$) are labeled with a non-overlapping αCD25-PE (MA251, BD Pharmingen (#555432)) and αCD45RO-FITC (UCHL1) and sorted into $CD25^+CD45RO^+$ (T_{Regs}) and $CD25^-$ populations on an ARIA flow cytometer. Purity of obtained populations is always > 98%. Either approach yields a population of $CD4^+CD25^+CD127^-$ T-cells which have been shown by us to function as T_{Regs}. To verify suppressive capacity amongst T_{Regs} (suppression assay), $5x10^4$ $CD4^+CD25^-$ responders are plated alone or with varying proportions of autologous T_{Regs} to a maximum ratio of 1:1 in complete T-cell media consisting of RPMI + 10% FCS, supplemented with HEPES buffer, sodium pyruvate, penicillin/streptomycin, L-glutamine, β-mercaptoethanol, and non-essential amino acids. After 72 hours of culture, levels of proliferation are assessed by ^{3}[H]-thymidine incorporation (Amersham, Piscataway, New Jersey). Cells are cultured for an additional 16 hours and then harvested on a FilterMate cell harvester (Perkin Elmer, Boston, Massachusetts). ^{3}[H] counts were performed using a Wallac 1450 Microbeta Trilux® Liquid Scintillation / Luminescence Counter (Perkin Elmer). Data are taken as means of triplicate wells. As a subordinate assay, to verify energy (proliferation assay) in the T_{Reg} population, $1x10^5$ T_{Regs} or $CD4^+CD25^-$ cells are plated alone in triplicate wells using phytohemagglutinin (PHA) (0.1, 1, 5, 10, 20 μg/well) as a stimulator and analyzed similarly.

8.2.1 Statistical Analysis

Our primary endpoint analysis will consist of a comparison of proliferative T-cell response to PHA among treatment groups (+/- daclizumab) using a generalized linear model for normal data that accounts for correlation of measurement replication across PHA doses (0, 0.1, 1, 10 μg/well) within subjects. This analysis determines whether the difference in treatment groups relative to proliferative response remains constant over 4 PHA doses (i.e. curves remain parallel or not). A test of interaction with 3 degrees of freedom will be used. If the curves deviate significantly from parallelism, we will conclude that the effect of treatment varies as a function of PHA dose level. Within the

context of this model, statistical contrasts will be used to assess treatment differences at specific PHA dose levels. If the test for interaction is not statistically significant, one will conclude that the treatment effect is constant over PHA dose levels. A generalized linear model without an interaction will be used to assess whether the main effect for treatment is statistically significant (i.e. treatment differences are non-zero). In this analysis and others, if assumptions required for t-tests are not satisfied, a non-parametric Wilcoxon rank sum test will be conducted at each PHA level. Significance for this and other assays will be taken at the α=0.05 level unless otherwise indicated. A Bonferroni correction will be used to control for the multiple comparisons.

8.3 T_{Reg} Counts

T_{Regs} levels will be determined before and after vaccination in each treatment group (+/- daclizumab), as described by us previously(Fecci, Mitchell et al. 2006), in whole blood and paired leukapheresis samples using combinations of titrated antibodies against CD3 (UCHT1), CD4 (RPA-T4), CD8 (RPA-T8), CD45RO (UCHL1), and CD25 (BD Biosciences, San Jose, California). Following incubation, Optilyse B (Immunotech, Marseille, France) are added to each sample. Cells are then re-incubated at RT for 15 minutes and diluted with distilled water. For intracellular FOXP3 staining, cells are washed and incubated for 1 hour with Fix/Perm Buffer (eBioscience, San Diego, California) and then washed and labeled with α-FOXP3 (PCH101, eBioscience) for 30 minutes in the dark at 4^oC in the presence of Permeabilization Buffer (eBioscience). Samples are washed and analyzed on an ARIA flow cytometer (BD Biosciences). Data analysis is performed using BD FloJo software. To further explore the potential mechanism of daclizumab in this setting, internalization of CD25 on T_{Regs} after daclizumab will be examined using a non-overlapping antibody (MA251) that does not block the IL-2 binding site. Anti-human secondary $F(ab')_2$ goat antibodies specific for both human IgG and IgM (Jackson ImmunoResearch, #109-116-127) that will recognize daclizumab bound to CD25 on the cell surface will also be used. This will provide data similar to that derived for our animal studies(Fecci, Sweeney et al. 2006).

8.3.1 Statistical Analysis

Assuming normal data distribution, an unpaired t-test will be used to compare treatment groups (+/- daclizumab) relative to mean T_{Reg} levels after vaccination. Because patients will be randomly assigned to each group, and both will receive equal vaccinations, differences between groups after vaccination should be attributable to daclizumab. An unpaired t-test will be used to examine baseline differences in T_{Reg} levels, however. As an exploratory analysis, a paired t-test will also be used within groups to determine differences in T_{Reg} counts before and after vaccination.

8.4 T-cell Cytokine Production

$CD4^+$ T-cells derived from patients in each group will be cultured in 96-well plates with 0, 5, or 10 μg of PHA or PEPvIII or KLH. After 16 and 72 hours of culture, supernatants are harvested and processed in duplicate with a custom BioRad Bio-Plex 7-plex (IL-2, IL-4, IL-6, IL-10, IL-12 (p70), IFN-γ, TNF-α) Cytokine Reagent Kit (BioRad) to differentiate responses into TH_1 and TH_2 types. Briefly, supernatants are incubated with anti-cytokine conjugated beads, followed by incubation with biotinylated

detection antibody. Reaction mixture is detected with streptavidin-PE and analyzed on a Luminex 100 machine (Luminex Corporation, Austin, Texas). Unknown cytokine concentrations are calculated by BioPlex Manager software using standard curves derived from a recombinant cytokine standard. Commercial ELISAs will be used to measure IL-7, IL-15, and TGF-β_{1-2} production as well.

8.4.1 Statistical Analysis

A two-sided t-test will be used to compare cytokine differences between treatment groups as described above.

8.5 EGFRvIII CD4$^+$ and CD8$^+$ Cytokine Fluorescent Cytometry

EGFRvIII-specific cellular immune responses will be monitored and evidence for the induction of autoimmunity will be sought. For cellular immune responses a polyfunctional analysis(Betts, Exley et al. 2005; Betts, Gray et al. 2006; Betts, Nason et al. 2006) of T-cell function will be performed as previously described(Betts, Exley et al. 2005). Briefly, cryopreserved PBMC samples are thawed and rested overnight at 37^{0}C/5% CO_2 in RPMI media containing 10% fetal calf serum (R10). The next day, the cells will be adjusted to 2x10^6/well and incubated with 1 µg/mL of each of the co-stimulatory MAbs αCD28 and αCD49d with or without peptide stimulation (2 µg/ml) in the presence of Brefeldin A (5 µg/ml Sigma-Aldrich, St. Louis, MO) monensin (1µg/ml; Golgistop, BD Biosciences, San Diego, CA), and αCD107a-Alexa 680 for 5-6 hr at 37^{0}C and 5% CO_2. Following stimulation, cells will be treated with EDTA for 15 minutes at ambient temperature (AT, 18-22ºC). The cells will be washed, and stained with MAbs specific for **CD4** (Cy5.5-PE), **CD8** Qdot 705, **CD45RO** PE-TR, **CD27** PE-Cy5, **CD57** Qdot 605, **CD14/CD19** (Cascade Blue) and a vital-dye reagent (LIVE/DEAD Fixable Violet Dead Cell Stain Kit for Flow Cytometry; Invitrogen Corp., CA) for 20 minutes at AT. After two washes, 1x BD FACS Lysing solution (BD Biosciences, San Jose, CA) will be added and samples will be incubated for 10 min at AT. After one wash, 1x BD FACS Permeabilizing Solution 2 (BD Biosciences, San Jose, CA) will be added and samples incubated for 10 minutes at AT. After one wash, cells will be stained with **αCD3** (Cy7-APC), **αIFN-γ** (FITC), **αTNF-α** (Cy7-PE), **αMIP1β** (PE), and **αIL2** (APC) for 30 minutes on ice, washed, and fixed in PBS containing 1% formaldehyde (Sigma-Aldrich, St. Louis, MO). In all experiments, a negative control (αCD28/49d), and a positive control (SEB, 10 µg/ml, Sigma-Aldrich) will be included. The samples are acquired on a custom LSRII polychromatic flow cytometer (BD Immunocytometry System, San Jose, CA) equipped for detection of 17 fluorescent parameters. We are planning to collect a minimum of 500,000 total lymphocytes from each sample, because we expect the frequency of responding cells to be between 0.05 and 1.0%. This number of events is required based on calculations performed by Dr. Holden Maecker (BD Bioscience, personal communication) to detect a statistically significant number of positive events that can be used for the analysis of the data and the characterization of the different populations. Antigens used in this assay will include PEPvIII, KLH, CEF peptide mix, total tumor lysate, and autologous normal brain lysate. Ideally, we would be able to select a validated surrogate immunologic response marker for clinical efficacy, but no such marker has been identified to date and studies to validate such a marker

would need to be large and prospective and would be clearly beyond the scope of this proposal. It would be our intent, however, to incorporate any additional knowledge that becomes available at the time of data analysis to evaluate the relative biologic significance of the immune response markers that we have chosen.

8.5.1 *Statistical Analysis*

The analysis used here will be one that has been successful at distinguishing HIV progressors and non-progressors based on T-cell phenotype(Betts, Exley et al. 2005; Betts, Gray et al. 2006; Betts, Nason et al. 2006). All data will be background subtracted. For each measure, a lower threshold corresponding to 2SD above background is set to 0 based on a Poisson model essentially allowing T-cells to be designated as positive or negative for a certain phenotypic marker. The number of positive phenotypic markers post-vaccination will be calculated for each patient. Treatment groups (+/- daclizumab) will be compared relative to the number of phenotypic markers observed post-vaccination for each antigen using a Wilcoxon rank sum test. Additional exploratory analyses will be conducted using a Fisher's exact test that will compare treatment groups with respect to the proportion of patients with $\geq$4 positive markers. Correlations between phenotypic markers and EGFRvIII loss on recurrence will also be sought.

8.6 Activation-induced Cell Death and *In Vivo* Proliferation

The frequency of proliferating versus apoptotic cells will be determined by intracellular expression of the proliferation marker Ki67 or active Caspase 3 in T-cell subsets, respectively. Freshly isolated PBMC cells (Sigma Accuspin tubes) or PBMC pulsed with PEPvIII-KLH or antigens of interest are stained for surface expression with anti-CD3/CD4/CD8/CD25 (commercially available from BD), washed in 1X PBS containing 1% BSA and 0.1% NaN_3 (PBS Wash) and red cell depleted with BD Lyse. Samples are then washed and fixed with FACS permeabilizing solution (Becton Dickinson), incubated with saturating amounts of anti-Ki67-FITC (Beckman-Coulter) and anti-active Caspase 3 (BD Pharmingen) (30 mins @ 4°C), and then washed and resuspended in 0.5mL PBS Wash with 0.4% paraformaldehyde. Multi-color flow cytometric acquisition/analysis of the samples will be performed on a BD LSRII flow cytometer (Becton Dickinson) in the Duke Human Vaccine Institute Flow Cytometry Facility. This LSRII instrument has two lasers (488nm, 633nm) and 7 fluorescent channels and 2 scatter channels. We routinely run similar 4-7 color panels on mouse, rhesus, and human cells to asses proliferation status in specific cell subsets(Sempowski, Hicks et al. 2005).

8.6.1 *Statistical Analysis*

All data will be background subtracted and compared between treatment groups using upaired t-tests as described above..

8.7 Delayed-type Hypersensitivity

To assess for patients' baseline and progressive status of cellular immunity, routine skin tests will be performed before the first immunization, and at the time of the fourth

vaccination. Patients will receive DTH skin test to the common recall antigens *Trichophyton*, *Candida* and Tetanus in order to try and assess immunocompetence.

8.7.1 Statistical Analysis

Assuming that all patients will have no response before vaccination, each patient will be classified as a responder or non-responder based upon an induration at the response site $\geq$10 mm. Fisher's exact test will be used to compare responses between groups after each vaccination.

8.8 PBMC Proliferation and Cytokine Secretion ELISA

Peripheral blood will be used to test antigen induced T-cell proliferation before each immunization. PBMC will be stimulated with PEPvIII peptide, KLH, a control peptide mix, (*HIV* pol), Tetanus, and the non-specific mitogens PHA and Con-A. The plates will be incubated at 37°C for 5 days. Proliferation will be measured by counting the incorporation of ^{3}H-thymidine in dividing cells. For detection of cytokine secretion, 1 mL cultures will be set up in 24 well plates using 5 x 10^{5} T-cells stimulated with PEPvIII-KLH. At the end of a 5-day incubation at 37°C the supernatant from the cultures will be removed, filtered and assayed for the presence of the cytokines IFN-γ, IL-2, IL-4 IL-5, TNF-α and IL-10, IL-12, IL-13, GM-CSF to determine the Th1/Th2 profile by BIO-Plex Cytokine Reagent Kit (171-30400, BIO-Rad, Hercules, CA 94547).

8.8.1 Statistical Analysis

All data will be background subtracted and compared using two-sided t-tests as described above.

8.9 Serology

Quantitative anti-EGFRvIII extracellular domain, PEPvIII, and KLH antibody concentration in the serum will be measured by FACS analysis of antigen-coated magnetic beads (Dyna-MIAT - Magnetic Immobilized Antigen Technique) in our laboratory. Briefly, antibody levels to specific antigens will be determined by FACS analysis of antigen immobilized beads as described in preliminary data section. Beads are reacted with serum, washed, and developed with fluorochrome conjugated polyclonal anti-human secondary. Antigens used in this assay will include EGFRvIII extracellular domain, PEPvIII, and KLH, CEF peptide mix, total tumor lysate, and autologous normal brain lysate. Bead performance is validated by comparing serum reactivity of antigen-specific beads to serum reactivity with antigen transfected cells. As another means of verification, sample serums will be pre-incubated with excess specific and non-specific antigens, insuring that human antibody binding is specific.

8.9.1 Statistical Analysis

All data will be background subtracted and compared using two-sided t-tests as described above.

8.10 NK Cell Assays

After gradient centrifugation, the lymphocyte population is identified based on characteristic forward and side scatter patterns, NK cells then are counted, sorted, (or isolated (NK Cell Isolation Kit II (negative selection), MACS, Miltenyi Biotec)) based on being $CD3^-$ and $CD56^+$ (BD Pharmingen) using a FACSAria. $CD56^{bright}$ cells are further differentiated by being $CX3CR1^-$ (MBL International)(Li, Lim et al. 2005). For cytokine analysis, purified populations (>95%) are placed in RPMI 1640 media with FCS and left unstimulated or stimulated with IL-12 (20 ng/mL) and IL-15 (100 ng/mL) (PeproTech) for 72 hours. Supernatants are analyzed by a Luminex 100 machine (Luminex Corporation, Austin, Texas) as described above.

To evaluate for the effects of NK cells on $CD4^+$ and $CD8^+$ T-cell proliferation overall and in an antigen-specific manner, PBMCs at baseline and after daclizumab therapy *in vivo* will be obtained from peripheral blood, cultured in X-VIVO 15 media ((Bio Whittaker) labeled with CFSE (Molecular Probes, 1μM), and activated either polyclonally with plate bound CD3 (20 ng/mL, OKT3 #16-0037) / CD28 (10μg/mL, CD28.2 #16-0289) (eBioscience)or with the specific antigen PEPvIII-KLH, or normal brain or tumor homogenate for 72 hours in the presence or absence of daclizumab (matched for sample). For half the samples, NK cells will be removed with CD56 beads (Miltenyi Biotec). The samples are then washed and re-seeded in media with IL-7 and IL-15. The numbers of T cells and NK cells is analyzed as described above.

The effect of daclizumab therapy on the cytotoxicity of NK cells against resting or activated T-cells will be tested using standard ^{51}Cr release assays. T-cells (T cell Isolation Kit II, MACS, Miltenyi Biotec) and NK cells (NK Cell Isolation Kit II (negative selection), MACS, Miltenyi Biotec) will be isolated and mixed at various effector:target ratios after labeling T-cells with ^{51}Cr overnight. For certain assay subsets $CD56^{bright}$ cells are further differentiated by being $CX3CR1^-$ (MBL International)(Li, Lim et al. 2005) using a FACSAria as described above. After 4 hours, at 37° and 5% CO_2, supernatants are harvested and counted. Data is analyzed with the spontaneous release subtracted.

8.10.1 Statistical Analysis

All analyses will be conducted as described above for T-cells.

8.11 Sub-clinical Autoimmunity

Patients will also be assessed before vaccine #1 and every 4 months after vaccine # 3 by a panel of clinical laboratory analyses to screen for the development of autoimmunity. The most common manifestations of autoimmunity seen in related trials(Phan, Yang et al. 2003; Attia, Phan et al. 2005; Blansfield, Beck et al. 2005; Jaber, Cowen et al. 2006) have included enterocolitis, dermatitis, uveitis, hepatitis, and hypophysitis. Laboratory evaluations to detect subclinical disease will be obtained as follows: serum thyroglobulin Ab (Lab Code 1802105), rheumatoid factor (Lab Code 5560195), antinuclear Ab (Lab Code 5560214) (Homma, Sagawa et al. 2006); human anti-human (anti idiotypic) Ab (Sampson Lab), erythrocyte sedimentation rate (Lab Code 2000860), thyroid-stimulating hormone (Lab Code 1802085), ACTH (Lab Code 1800020), cortisol (Lab Code1802010) , free T4 (Lab Code 1802081), ALT, and AST (Lab Code 1004000). Finally, if

cerebrospinal fluid becomes available, it will be checked for oligoclonal banding (Lab Code 1543440).

8.11.1 Statistical Analysis

For laboratory screening tests of autoimmunity, any change from a normal to an abnormal value or a 2 fold change will be considered a positive result. A patient with any "positive" test will be considered positive and the number of positive tests will not be weighted. A 2 x 2 table will then be constructed to differentiate the frequency of positive patients by treatment group. A Chi^2 test will then be used to assess if these frequencies are statistically significant.

8.12 Whole Blood for Quantitative PCR for EGFRvIII

Before each immunization, 10 ml of whole blood from each patient will be collected for total RNA extraction. The mononuclear cell fraction of 10 ml of EDTA treated blood was separated by gradient density centrifugation with Ficoll. A total of $5 \times 10^6 – 1 \times 10^7$ viable cells were used for total RNA extraction with QIAamp RNA Blood Mini kits (Qiagen Cat# 52304) according to manufacture's instruction. RNA was quantified spectrophotometrically at 260 nm and stored at -80 ^oC. 1st strand cDNA is synthesized from 1-5 ug of each total RNA with SuperScriptase III (Invitrogen Cat# 18080-051) by following the supplied protocol.

EGFRvIII & EGFR are detected with nested PCR by Taq DNA polymerase Kit with Q-solution (Qiagen) and dUTP substituted for dTTP (Silva, Abraul *et al*. 2006). The 1st round external PCR is mixed in a 50 µL reaction [containing 5–8 µl of cDNA, 1X buffer 1X Q-solution , 200 µM of dNTPs (dATP, dGTP, and dCTP), 600 µM of dUTP, 0.5 µM of each primer (forward: 5'-GTA TTG ATC GGG AGA GCC G-3'; reverse: 5'-GTG GAG ATC GCC ACT GAT G-3', which cross several exons to assure amplifying cDNA only), 2.5 mM of MgCl2, 3U of Taq DNA polymerase and 1 U of N-uracyl glycosylase (UNG)] and amplified on a thermal cycler [5 min at 25 ^oC for UNG reaction, 2min at 95 ^oC for UNG inactivation, 40 cycles of 45 sec at 95° C, 45 sec at 60 ^oC and 90 sec at 72 ^oC]. 5 µl of the 1st PCR was used in 2nd internal PCR with primers (forward: 5'-GCG ATG CGA CCC TCC GGG-3'; reverse primer 5'-TCC GTT ACA CAC TTT GCG-3' which spans exons 8 and 9 junction to eliminate genomic DNA amplification) at the condition (1 cycle of 94 ^oC for 2 min followed by 35 cycles of 45 sec at 95 ^oC, 1 min at 55 ^oC and 1 min at 72 ^oC). 8 µl of cDNA in 1st PCR and 5 µl of 1st PCR in 2nd PCR are used in the higher RNA dilutions in sensitivity assays with patients' blood, positive control (EGFR or EGFRvIII expressing cell lines) and negative control samples (normal nucleated blood cells).

352 bp of 1st PCR & 219 bp of 2nd PCR are amplified from EGFRvIII cDNA (GenBank NM_201283). Both 1st & 2nd PCR products from EGFR cDNA are 801 bp bigger than from EGFRvIII cDNA resolved on a 1.5% agarose electrophoresis gel stained with ethidium bromide (7-10 µL of PCR products each).

Quality of cDNA synthesis is verified by detecting a housekeeping gene glyceraldehyde-3-phosphate dehydrogenase (GAPDH) cDNA (GenBank BC029618) with a 171 bp PCR product of cDNA at above PCR condition except adding less amount of cDNA (1-2 µl of cDNA), changing to GAPDH primers (forward: 5'-TCG CCA GCC

GAG CCA CAT CG-3'; reverse: 5'-GAA CAT GTA AAC CAT GTA G-3') and maintaining 35 cycles with 56 ^{o}C of annealing temperature.

The detection of EGFR or EGFRvIII are confirmed by sequencing & aligning the products of the 2nd PCR obtained from positive control samples and from randomly selected positive blood samples. The specificity was also confirmed with no detection of RT-nested PCR in cell lines that do not express EGFRvIII or EGFR and normal nucleated blood cells.

8.12.1 Statistical Analysis

All data will be background subtracted and compared using **two-sided t-tests** as described above.

9 SAFETY MONITORING

9.1 Potential Benefits

Based on experience with immunization in our previous vaccine trials, immunotherapy may be of benefit to subjects with MGs. Of course, because individuals respond differently to therapy, no one can know in advance if it will be beneficial in an individual case. The potential benefits may include reduction and/or remission of the subject's brain cancer. Because this procedure is experimental, it cannot be guaranteed that subjects will receive any benefit as a result of participating in this research study. The information collected in this research may help scientists better understand the mechanisms involved in the immune system's ability to fight cancer. If such an understanding comes from this research, then it may benefit society by furthering the development of improved treatment methods for human malignant brain tumors in the future.

9.2 Potential Risks

9.2.1 Allergic Reactions to EGFRvIII Vaccination

Injection of PEPvIII peptide or KLH may result in an allergic reaction, which could include redness and swelling at the injection site, itching, hives, low blood pressure, difficulty breathing, or in the most extreme circumstances, death. In addition, if the immune system becomes overly activated, potential discomforts may include pain, redness and swelling at the injection site.

9.2.2 GM-CSF

Injection of GM-CSF may increase the risk of infection, lower platelets, or cause fluid retention. GM-CSF also may result in an allergic reaction, which could include redness and swelling at the injection site, itching, hives, flushing, syncope, low blood pressure, difficulty breathing, or in the most extreme circumstances, death. In addition, if the immune system becomes overly activated, potential discomforts may include pain, redness and swelling at the injection site.

9.2.3 Cerebral Edema

Cerebral edema may be secondary to the disease process itself, the surgical procedure, necrosis from previous radiation, or inflammation due to immune infiltration of the brain or destruction of tumor cells. Symptoms may include, but are not limited to, severe headache, confusion, lethargy, unresponsiveness, coma, or focal neurological deficits. Patients will be monitored throughout the course of the study and those patients with any signs or symptoms of cerebral edema may need their steroid doses increased, treatment with an osmotic diuretic, or surgical decompression. Edema that fails to respond to aggressive therapy may lead to permanent neurological impairment. The probability of this risk can be predicted to some degree based upon tumor size, location,

pre-operative neurological impairment, and post-operative course prior to vaccinations. Patients will be monitored throughout the course of the study.

9.2.4 Infection

The CDX-110 vaccine may include the risk of infection due to potential contamination of the leukocyte product in the laboratory. This may result in localized redness, swelling, or induration at the injection site. In the most extreme situation, this may lead to systemic bacterial/fungal sepsis and possibly death. The probability of this risk is relatively low, given the small injection volume (1 mL divided between >2 intradermal locations) and the fact that the CDX-110 will be strictly tested for sterility prior to each injection. The risk of infection due to potential contamination of the CDX-110 in the laboratory will be minimized by biosafety quality assurance and testing. All cell cultures will be handled under sterile conditions in a core tissue culture facility dedicated to the processing of human cells. Prior to injection into patients, vaccines must pass sterility tests in thiglycolate broth, tryptic soy blood agar, and inhibitory Sabouraud agar. Following injections, patients will be monitored throughout the course of the study for any signs and symptoms of infection. There have been no infections to date in the recent VICTORI (IRB #3108-05-9R4) or ACTIVATE (IRB #5421-05-1R1) clinical trials testing similar approaches in a similar patient population. If an active infection is suspected, patients will be cultured and treated with appropriate antibiotics.

9.2.5 Delayed Autoimmune Diseases

It is possible that delayed autoimmune disease(s) may develop as a result of injection with CDX-110. This means that the immune system may be stimulated to attack natural tissue in the body. Animal studies have reported the development of autoimmunity in the context of vaccination and recovery from lymphopenia. However, our current experience with CDX-110 vaccination in glioma patients has not demonstrated evidence of autoimmunity in treated patients. Furthermore, the doses of TMZ used in this study for induction of lymphopenia are standard doses administered to patients with GBM. It therefore, is unknown what the risk of delayed autoimmune disease is for this study.

9.2.6 Phlebotomy

Drawing blood or inserting an intravenous catheter into an arm vein may result in bruising or swelling in the area of the insertion, bleeding at the site of the needle puncture, light headedness, fainting and very rarely, local infection, which may be severe. These risks are reduced by the fact that the blood will be drawn by a qualified physician, nurse or phlebotomist (a professional trained to draw blood).

9.2.7 Leukapheresis

As with any donation of blood, a variety of minor reactions may occur with leukapharesis, which include fainting, dizziness, or nausea. Uncommon but serious complications may also result, which include bleeding, infection, an adverse reaction to

the anticoagulant or replacement fluids, hypocalcemia, hypotension, shock, convulsions, air emboli, heart failure, or the inability to transfuse blood back into the patient. These risks are reduced by the fact that the procedure will be performed by qualified staff at a specialized clinical hemapheresis unit. Patients will be carefully monitored throughout the procedure by trained nursing and medical staff. Calcium gluconate (2 mg PO) will be given to minimize the risks of hypocalcemia, fluid supplementation will be given to minimize hypotension, and blood will be routinely screened for *HIV*, hepatitis, and syphilis to minimize the risk of transmitting infection.

9.2.8 MRI

The risks and/or discomforts associated with the performance of MRI include the anxiety produced from being in a tight, enclosed space (claustrophobia). In addition, the machine operates using a large and powerful magnet. The magnetism of the machine attracts certain metals: therefore, people with these metals in their bodies (specifically pacemakers, infusion pumps, metal aneurysm clips, metal prostheses, joints, rods or plates) will be excluded from the study. Patients will also be checked to make sure that they do not bring any metal objects into the MRI facility. Dental fillings are less affected by the magnetic fields generated and are therefore permitted. It will be asked that patients let the physicians conducting this study know of any metal in their bodies other than dental fillings.

9.2.9 Allergic Reactions to Contrast Agents

During the MRI, patients will be given a contrast agent. The agent is given routinely to obtain enhanced MRI scans of the brain. The agent is administered through the vein and requires the placement of an IV catheter. The catheter placement is similar to drawing blood except that the catheter remains in the vein during the time the agent is actively delivered. The risks of a blood draw and insertion of a catheter are similar. There have been a few, rare cases of allergies to the agent used in MRI contrast enhanced scans. Patients with any known severe allergies to contrast agents will be excluded from the study. Patients with mild allergies (i.e., rash only) will be pretreated with Tylenol and Benadryl prior to injection of the contrast agent.

9.2.10 Temozolomide

TMZ has been well tolerated by both adults and children with the most common toxicity being mild myelosuppression. Other, less likely, potential toxicities include nausea and vomiting, constipation, headache, alopecia, rash, burning sensation of skin, esophagitis, pain, diarrhea, lethargy, hepatotoxicity, anorexia, fatigue and hyperglycemia. Hypersensitivity reactions have not yet been noted with TMZ. As in the case with many anti-cancer drugs, TMZ may be carcinogenic. Rats given TMZ have developed breast cancer. The significance of this finding for human is not presently known. TMZ therapy will be followed but given as standard of care. If toxicities occur, the Principle investigator and primary physician will titrate therapy based on standard clinical guidelines as outlined above.

9.2.11 Daclizumab

In trials of renal transplantation, the overall incidence of infectious episodes, including viral infections, fungal infections, bacteremia and septicemia, and pneumonia was not higher in the daclizumab–treated patients than the placebo-treated patients. There was, however, a slight increase in wound infections, specifically, with daclizumab. Addition of daclizumab also did not increase the number of post-transplant lymphomas up to three years post-transplant. No difference in abnormal hematologic or chemical laboratory test results were seen between groups treated with placebo or daclizumab with the exception of fasting blood glucose which was likely related to corticosteroids or diabetes. While low titers of anti-idiotype antibodies to daclizumab were detected in adult patients treated with the drug, no antibodies that affected efficacy, safety, serum daclizumab levels or any other clinically relevant parameter examined were detected.

9.2.12 Unknown Risks

The overall risk classification of this research is unknown. Clinical trials using CDX-110-based immunizations on brain tumor patients only recently published in the literature. From our experience with 40 patients in ongoing and previous trials we have not seen any toxicities or serious unexpected adverse events.

9.2.13 Confidentiality

Participation in research investigations may result in a loss of confidentiality. However, all data from preoperative and postoperative evaluations will be coded to protect the patient's identity. The coding, and the results of these studies will be available only to the individuals involved with the study, the clinical staff administering the study, representatives of the National Institutes of Health, and representatives of the U.S. Food and Drug Administration. Any publications resulting from this study will not use patient identifying data.

9.2.14 Treatment Alternatives & Financial Reimbursement

Alternative treatments for recurrent malignant brain tumors include additional surgery, radiation, and/or chemotherapy. If the patient chooses not to participate in this trial, they certainly may seek alternative treatment. If the patient fails treatment through this trial, these alternatives may still be available to the patient. There will be no financial reimbursement to subjects for study participation.

9.3 Data and Safety Monitoring Plan

This clinical research study will be monitored both internally by the PI and externally by the Duke University Medical Center Cancer Center's Protocol Review and Monitoring system in accord with their NCI-approved "Institutional Protocol Monitoring Procedures and Guidelines for NIH-sponsored Research Involving Human Subjects." In terms of internal review the PI will continuously monitor and tabulate adverse events. Appropriate reporting to the Duke University Medical Center IRB will be made. If an

unexpected frequency of grade III or IV events occur, depending on their nature, action appropriate to the nature and frequency of these adverse events will be taken. This may require a protocol amendment, dose de-escalation, or potentially closure of the study. The PI of this study will also continuously monitor the conduct, data, and safety of this study to ensure that:

Interim analyses occur as scheduled;
Stopping rules for toxicity and/or response are met;
Risk/benefit ratio is not altered to the detriment of the subjects;
Appropriate internal monitoring of adverse events and outcomes is done;
Over-accrual does not occur;
Under-accrual is addressed with appropriate amendments or actions;
Data are being appropriately collected in a reasonably timely manner.

External review of this protocol begins with an initial review by the CPC, which performs a risk assessment of the trial. The PI will abide by their assessment of the level of risk, which will determine the intensity of subsequent external monitoring. Documentation of this assessment will be maintained. A formal, independent audit will be conducted by the Scientific Monitoring Subcommittee of the CPC after the first 3 subjects are enrolled. Assuming a "satisfactory" review, thereafter, the Scientific Monitoring Subcommittee of the CPC will review selected patients enrolled on this protocol on a bi-annual basis. This includes review of aggregate toxicity as reported in the progress report, accrual, amendments, and outcomes. If there appears to be an unexpected frequency of serious and/or unexpected toxicity or other significant issues, a full re-audit will be initiated. Any rating less than satisfactory will also prompt a full re-audit and corrective plan.

10 STATISTICAL CONSIDERATIONS

10.1 Recruitment

The following table lists the gender and minority breakdown for all brain tumor patients seen at Duke relative to the national incidence figures:

PERCENTAGE

	DUMC (1992-1996) n=793			NATIONAL		
	MALE	FEMALE	TOTAL	MALE	FEMALE	TOTAL
WHITE	55.5%	32.3%	87.7%	48.2%	34.4%	82.6%
AFRICAN AMERICAN	5.7%	4.0%	9.7%	3.6%	3.6%	7.2%
NATIVE AMERICAN	0.3%	0.3%	0.5%	0.1%	0.1%	0.2%
ASIAN	0.4%	0.3%	0.6%	0.7%	1.0%	1.7%
HISPANIC	0.9%	0.5%	1.4%	1.8%	0.8%	2.6%
OTHER	0.0%	0.0%	0.0%	3.3%	2.4%	5.7%
TOTAL	62.7%	37.3%	100.0 %	57.7%	42.3%	100.0%

Since the Duke Brain Tumor Program began in 1996 we have treated patients from 35 States and 11 foreign countries. The national incidence figures are the most accurate with which to compare our accruals. Our brain tumor studies are open to patients of all ages, both sexes, and all minorities. The only exclusions are the occasional incidences in which the FDA or our IRB[19] requires that children below the age of 18 be excluded from some of our initial Phase I studies because of the potential for greater toxicity to the developing nervous system. In addition, certain diseases that we treat occur preferentially in one gender or minority than another. For example, carcinoma of the breast is a frequent cause of metastatic brain tumors and, of course, is much more common in women than in men. Although the lung cancer incidence is rising in women, the incidence is still higher in men and it is also a frequent cause of metastatic brain tumors. Primary brain tumors and metastatic brain tumors from some very common tumors such as melanoma are an infrequent disease in African-American populations. Our accrual statistics reflect the lower incidence in the African-American population. Although our accrual historically has been weighted in favor of white males, we have previously demonstrated that this bias was not present when the demographics of the patient population that was offered participation was considered, but rather only in that population that consented to participate. We will continue to actively recruit women and minorities into this study using methods that have shown previous success in recruiting the participation of these subgroups.

We routinely send letters to our primary referring physicians notifying them of our interest in including minorities and women in all our brain tumor clinical trials. The overall Duke figures for minority patients with brain tumors exceed, in most instances, the national incidence figures.

In order to ensure representative accrual to our studies, the table below has been created. It outlines estimated accrual and anticipated ethnic and sex composition for the proposed studies. Actual accrual will be reviewed after each segment and compared with

[19] IRB, institutional review board

this table. If the expected accrual diversity is not met, we will initiate a targeted recruitment program through techniques already established within the Duke University Medical Center including the placement of advertisements in newspapers or on radio stations catering to the underrepresented population(s) and providing educational seminars for patients and staff at community institutions such as churches and medical clinics that preferentially serve the underrepresented population(s).

Twenty patients will be accrued to this study. All patients enrolled on the study, even if taken off study prior to treatment, will be included in the data analysis for this section and those outlined below. Based on our proven accrual rate of >1 EGFRvIII$^+$ patients per month in our completed studies and the absence of any other competing studies within the Brain Tumor Center at Duke, accrual to this study is estimated to take ~20 months to complete. It is estimated that an additional 6 months will be needed to analyze immunologic data and another 12 months to complete patient follow-up for clinical endpoints. The target distribution for patient enrollment is shown in the following Table:

**Number of Patients:**		20	
**Race**	**Male**	**Female**	**TOTAL**
**American Indian/Alaska Native**	0	0	
**Asian**	0	0	
**Native Hawaiian or Other Pacific Islander**	0	0	
**Black or African-American**	1	1	
**White**	10	8	
**TOTAL**	11	9	20
**Ethnicity**			
**Hispanic or Latino**	0	0	
**Not Hispanic or Latino**	11	9	20

A second cohort of 6 patients (cohort #2) will receive serial administrations of daclizumab at vaccines # 1 and 3 and during the recovery from first 3 TMZ cycles at the same dose. Patients with progressive disease during radiation, dependent on steroid supplements above physiologic levels at time of vaccination, and are unable to tolerate TMZ will be replaced. As a result, 12 patients will be consented but a maximum of 6 patients will receive 3 vaccines and 5 cycles of daclizumab. In cohort #2, patients who do not complete 4 vaccines and 2 doses of daclizumab will be replaced. Based on a predicted accrual rate of 2 patients each month, it is estimated to take 3-6 months to complete this additional cohort. The target distribution for patient enrollment is shown in the following Table:

	Patient Numbers	White		African-American		Hispanic		Asian		Other	
		Male	Female	Male	Female	Male	Female	Male	Female	Male	Female
Cohort #1	20	10	7	1	1	0	0	0	0	1	0
Cohort #2	6	3	2	1	0	0	0	0	0	0	0
TOTAL	26	13	9	2	1	0	0	0	0	1	0

10.2 Safety Component

The study statistician, James Herndon, II, Ph.D., will be responsible for all statistical analysis. Establishing the safety and efficacy of intradermal immunization with CDX-110 with or without daclizumab will be an important goal of this study and the methods for determining this are outlined above.

Given the lack of significant toxicity in our prior multi-institutional EGFRvIII-targeted immunotherapy trials and those of others in patients with GBM(Yu, Wheeler et al. 2001), and the established safety of the clinically–approved drug daclizumab, and the safety of other clinical approaches to T_{Reg} inhibition that have been used recently(Dannull, Su et al. 2005), we believe that it is likely that no significant toxicity will occur. However, any irreversible Grade 3 or reversible Grade 4 toxicity or life-threatening event, prior to tumor progression, will be considered a limiting toxicity. Patients will be randomized to each group in blocks of 3 and patients will be evaluated for toxicity within these groupings. If no patients in a given cohort experience a toxicity within 2 weeks another cohort will be enrolled within that arm. Enrollment will be halted if:

1. Any two patients in a cohort of three experience a DLT; or,
2. Any two patients in a cohort of six consecutively enrolled patients experience a DLT.

Because we have previously safely treated many patients with the vaccine alone, toxicity in each arm will be evaluated independently. We have an existing Data and Safety Monitoring Plan approved by the NCI (See Appendices) for use in these studies.

10.3 Time to Progression and Survival

Patients enrolled on this study who are confirmed to have a high grade glioma will be followed for all parameters until death. TTP is not a primary endpoint in this study but will be accessioned to derive hypotheses that may relate other data derived in this study with progression. Clinical and immunologic endpoints will be analyzed in all patients and statistical considerations powered based on enrollment of 20 patients. However, an analysis of patients stratified at enrollment based on complete resection or residual disease will be performed for hypothesis generation on the role of tumor burden on clinical and immunologic responses to CDX-110 vaccination and daclizumab.

Survival is not an endpoint in this study but will be accessioned to derive hypotheses that may relate data derived in this study with survival times. Patients will also be assigned a prognosis based on published recursive partitioning analysis of prognostic factors(Curran, Scott et al. 1993). Survival will be calculated from the date diagnostic tissue is first obtained.

Although not powered for survival analyses, the product limit estimator of Kaplan and Meier(Kaplan and Meier 1958) will be used to describe the distribution of survival time and TTP for both treatment groups, and the Cox proportional hazards model(Cox

1972) will be used to compare TTP and survival between the groups and with appropriate matched historical control groups. We would consider daclizumab worthy of further evaluation only if it did not significantly reduce TTP or overall survival relative to the control group and relative to the historical controls. Therefore, in the proposed study, we are interested in determining whether either treatment regimen is definitely worse than our best contemporaneous results at Duke University which are with the use of radiolabeled MAbs delivered intratumorally. The most recent Phase II trial evaluating radiolabeled MAbs at Duke in patients with newly-diagnosed GBM had a median survival of 79.4 weeks, and the lower limit of the 95% confidence interval was 61.4 weeks. Assuming that survival is exponential in this context, a median of 61.4 weeks translates into a 6-month survival of approximately 75%. Therefore if 6-month survival is not definitively <75%, there would remain interest in further development of the regimen proposed here. With 10 patients in each arm of our trial, one can conduct a test with $\alpha=\beta=0.1$ to differentiate between a 6-month survival rate of 35% and 75%. That means that if the true 6-month survival rate were $\leq$35% there is <10% chance of concluding that the treatment regimen is worthy of pursuing with further investigation. Similarly, if the true 6-month survival rate were $\geq$75% or more there is $\leq$10% chance of rejecting the treatment. Other comparison groups that could be used include a historical control database, the recently published Phase III trial of the regimen of TMZ to be used in this trial, or our previous trial dataset using this vaccine approach which had median survivals (95% CI) of 14.7 (12,0, 17.4), 14.6 (10.9, 21.2), and >29.8 months, respectively. In addition, we will assess clinical efficacy by estimating the proportion of patients who survived longer than expected according to Curran's recursive partition analysis(Curran, Scott et al. 1993).

10.3.1 Identification of Covariates

Kaplan and Meier survival curves will be constructed and the Cox proportional hazards model will be used to identify continuous or discrete variables associated with TTP or survival from the immunologic measures and measures of the following predictors: serum EGFRvIII qPCR; serum anti-EGFRvIII titers; EGFRvIII-specific, IFN-γ producing, CD8+/CD27–/CD28–/CD45RA–effector memory cells, 5-color positive cells, PEPvIII-KLH-specific tetramer-positive cells, and T_{Reg} number and function. Given the large number of predictors that will be examined relative to the overall sample size, it is recognized that these analyses will be exploratory. These analyses are conducted to generate hypotheses only and will need to be validated in additional patients or studies. Statistically, joint effects will also be explored, but are unlikely to have sufficient power to identify such interactions. Assuming that half the sample size of 24 patients are positive for a particular measure and that all have died, we will have 80% power for a logrank test conducted with $\alpha=0.05$ to detect a survival hazard ratio of approximately 5.04 comparing patients with a positive and negative measure.

In addition, logistic regression and Fisher's exact Chi^2 test will be used to explore whether the covariates outlined above predict the binary outcome of whether or not a patient survives longer than predicted according to Curran's(Curran, Scott et al. 1993) or Lamborn's(Lamborn, Chang et al. 2004) models. For example, for a continuous

predictor dichotomized at its median, given our patient sample size of 20, we will have 80% power to detect a difference in the “success” rate between 30% and 97% with α=0.05.

11 STUDY FLOW SHEET

Appointment	Time and Range	Curran Rating<br>EGFRvIII screen<br>*HIV* Screen<br>Hepatitis	Physical, Ophthalmologic, and Neurologic Exam<br>MMSE<br>KPS	MRI[20]	EGFRvIII PCR<br>EGFRv III ELISA<br>Blood for Autoimmunity and Immunologic monitoring[21]	DTH[22]	ABC[23]<br>T4/T8	CMP[24]<br>β-HCG[25]
**Screening and Pre-enrollment**	Week ≤4 from leukapheresis	X					X	
**Pre-Immunization Leukapheresis**	Prior to Temozolomide		X	X[26]	X	X	X	X
**Radiation with Temozolomide**	6 ± 2 weeks duration						X	
**Initial Temozolomide Cycle**	3 ± 1 week post RT (Days 1-5)		X	X[27]			X	
**Immunization #1 (Daclizumab*)***	Day 21 ± 2		X		X		X	
**Immunization #2**	Day 35 ± 7		X				X	
**Immunization #3(Daclizumab cohort#2)**	Day 49 ± 7		X			X	X	
**Temozolomide Cycles**	Every 4 weeks (Days 1-5)						X	
**Immunization #4 (even) (Daclizumab cohort#2)[28]**	Day 21 ± 2 of cycle		X	X	X	X	X	
**Immunization #5 (odd)**	Day 21 ± 2 of cycle		X		X		X	
**EGFRvIII +DTH**	+DTH		X		X		X	X
**Progression**			X	X	X	X	X	

[20] MRI every 8 ± 4 weeks.
[21] 10 Yellow top ACD (9 mL each) and 3 Red top (9 mL each) - all **prior** to vaccine except prior to vaccines #2 and #3 where only 4 Yellow top ACD (9 mL each) and 3 Red top (9 mL each) will be obtained . With vaccine #4, blood work for immunological monitoring will occur monthly before each vaccine. Blood work for autoimmunity monitoring will be obtained prior to vaccine # 1 and every 4 months after vaccine # 3.
[22] Should be read 48-72 hours after placement
[23] All ABC should be obtained with manual differential counts. ABC should always be obtained with T4/T8 counts. These should be obtained at least weekly during concurrent external beam radiation and temozolomide, immediately before and within 1h after ALT, and then subsequently on day of vaccine and immediately prior to next temozolomide cycle.
[24] Comprehensive Metabolic Panel (CMP)
[25] Obtained BEFORE each leukapheresis only. B-HCG only required for females.
[26] MRI here is just post-operative eligibility MRI
[27] Post-radiation MRI will be done a minimum of 10 days after completing radiation therapy.
[28] Cohort 2 will receive daclizumab at same dose with vaccine # 1, # 3, and on day 21 ± 2 for 3 TMZ cycles.

12 REFERENCES

Abramowicz, D. and D. Abramowicz (1998). "Daclizumab to prevent acute rejection in renal transplantation.[comment]." New England Journal of Medicine **338**(23): 1700-1.

Albert, M. L., M. Jegathesan, et al. (2001). "Dendritic cell maturation is required for the cross-tolerization of CD8+ T cells." Nat Immunol **2**(11): 1010-7.

Albright, L., J. A. Seab, et al. (1977). "Intracerebral delayed hypersensitivity reactions in glioblastoma multiforme patients." Cancer **39**(3): 1331-1336.

Aldape, K. D., K. Ballman, et al. (2004). "Immunohistochemical detection of EGFRvIII in high malignancy grade astrocytomas and evaluation of prognostic significance." Journal of Neuropathology & Experimental Neurology **63**(7): 700-7.

Antony, P. A., C. A. Piccirillo, et al. (2005). "CD8+ T Cell Immunity Against a Tumor/Self-Antigen Is Augmented by CD4+ T Helper Cells and Hindered by Naturally Occurring T Regulatory Cells." Journal of Immunology **174**(5): 2591-2601.

Arina, A., I. Tirapu, et al. (2002). "Clinical implications of antigen transfer mechanisms from malignant to dendritic cells. exploiting cross-priming." Exp Hematol **30**(12): 1355-64.

Armstrong, T. D., B. A. Pulaski, et al. (1998). "Tumor antigen presentation: changing the rules." Cancer Immunol Immunother **46**(2): 70-4.

Asano, M., M. Toda, et al. (1996). "Autoimmune disease as a consequence of developmental abnormality of a T cell subpopulation." Journal of Experimental Medicine **184**(2): 387-96.

Asavaroengchai, W., Y. Kotera, et al. (2002). "Tumor lysate-pulsed dendritic cells can elicit an effective antitumor immune response during early lymphoid recovery." Proceedings of the National Academy of Sciences of the United States of America **99**(2): 931-6.

Ashley, D. M., B. Faiola, et al. (1997). "Bone marrow-generated dendritic cells pulsed with tumor extracts or tumor RNA induce antitumor immunity against central nervous system tumors." Journal of Experimental Medicine **186**(7): 1177-1182.

Attia, P., A. V. Maker, et al. (2005). "Inability of a fusion protein of IL-2 and diphtheria toxin (Denileukin Diftitox, DAB389IL-2, ONTAK) to eliminate regulatory T lymphocytes in patients with melanoma." Journal of Immunotherapy **28**(6): 582-92.

Attia, P., A. V. Maker, et al. (2005). "Inability of a fusion protein of IL-2 and diphtheria toxin (Denileukin Diftitox, DAB389IL-2, ONTAK) to eliminate regulatory T lymphocytes in patients with melanoma." 582-92, 2005 Nov-Dec.

Attia, P., G. Q. Phan, et al. (2005). "Autoimmunity correlates with tumor regression in patients with metastatic melanoma treated with anti-cytotoxic T-lymphocyte antigen-4." Journal of Clinical Oncology **23**(25): 6043-53.

Attia, P., D. J. Powell, Jr., et al. (2006). "Selective elimination of human regulatory T lymphocytes in vitro with the recombinant immunotoxin LMB-2." Journal of Immunotherapy **29**(2): 208-14.

Attia, P., D. J. Powell, Jr., et al. (2006). "Selective elimination of human regulatory T lymphocytes in vitro with the recombinant immunotoxin LMB-2." 208-14, 2006 Mar-Apr.

Azuma, T., T. Takahashi, et al. (2003). "Human CD4+ CD25+ regulatory T cells suppress NKT cell functions." Cancer Research **63**(15): 4516-20.

Baan, C. C., M. J. Boelaars-van Haperen, et al. (2001). "IL-7 and IL-15 bypass the immunosuppressive action of anti-CD25 monoclonal antibodies." Transplantation Proceedings **33**(3): 2244-6.

Bagavant, H., C. Thompson, et al. (2002). "Differential effect of neonatal thymectomy on systemic and organ-specific autoimmune disease." International Immunology **14**: 1397-1406.

Baruah, P., A. Propato, et al. (2006). "The pattern recognition receptor PTX3 is recruited at the synapse between dying and dendritic cells, and edits the cross-presentation of self, viral, and tumor antigens." Blood **107**(1): 151-8.

Baselga, J. (2001). "The EGFR as a target for anticancer therapy--focus on cetuximab." Eur J Cancer **37 Suppl 4**: S16-22.

Batra, S. K., S. Castelino-Prabhu, et al. (1995). "Epidermal growth factor ligand-independent, unregulated, cell-transforming potential of a naturally occurring human mutant EGFRvIII gene." Cell Growth & Differentiation **6**(10): 1251-1259.

Betts, M. R., B. Exley, et al. (2005). "Characterization of functional and phenotypic changes in anti-Gag vaccine-induced T cell responses and their role in protection after HIV-1 infection." Proceedings of the National Academy of Sciences of the United States of America **102**(12): 4512-7.

Betts, M. R., C. M. Gray, et al. (2006). "Antigen-specific T-cell-mediated immunity after HIV-1 infection: implications for vaccine control of HIV development." Expert Review of Vaccines **5**(4): 505-16.

Betts, M. R., M. C. Nason, et al. (2006). "HIV nonprogressors preferentially maintain highly functional HIV-specific CD8+ T cells." Blood **107**(12): 4781-9.

Bielekova, B., M. Catalfamo, et al. (2006). "Regulatory CD56(bright) natural killer cells mediate immunomodulatory effects of IL-2Ralpha-targeted therapy (daclizumab) in multiple sclerosis." Proceedings of the National Academy of Sciences of the United States of America **103**(15): 5941-6.

Bielekova, B., N. Richert, et al. (2004). "Humanized anti-CD25 (daclizumab) inhibits disease activity in multiple sclerosis patients failing to respond to interferon beta.[erratum appears in Proc Natl Acad Sci U S A. 2004 Dec 14;101(50):17565]." Proceedings of the National Academy of Sciences of the United States of America **101**(23): 8705-8.

Bigner, D. D., O. M. Pitts, et al. (1981). "Induction of lethal experimental allergic encephalomyelitis in nonhuman primates and guinea pigs with human glioblastoma multiforme tissue." Journal of Neurosurgery **55**(1): 32-42.

Bigner, S. H., P. A. Humphrey, et al. (1990). "Characterization of the epidermal growth factor receptor in human glioma cell lines and xenografts." Cancer Research **50**: 8017-8022.

Blansfield, J. A., K. E. Beck, et al. (2005). "Cytotoxic T-lymphocyte-associated antigen-4 blockage can induce autoimmune hypophysitis in patients with metastatic melanoma and renal cancer." Journal of Immunotherapy **28**(6): 593-8.

Bloom, H. J., M. J. Peckham, et al. (1973). "Glioblastoma multiforme: a controlled trial to assess the value of specific active immunotherapy in patients treated by radical surgery and radiotherapy." British Journal of Cancer **27**(3): 253-267.

Boockvar, J. A., D. Kapitonov, et al. (2003). "Constitutive EGFR signaling confers a motile phenotype to neural stem cells." Molecular & Cellular Neurosciences **24**(4): 1116-30.

Boonman, Z. F., G. J. van Mierlo, et al. (2005). "Maintenance of immune tolerance depends on normal tissue homeostasis." J Immunol **175**(7): 4247-54.

Borrow, P., J. L. Cornell, et al. (1995). "Immunization-induced inflammatory infiltration of the central nervous system in transgenic mice expressing a microbial antigen in astrocytes." Journal of Neuroimmunology **61**(2): 133-149.

Boskovitz, A., C. J. Wikstrand, et al. (2004). "Monoclonal antibodies for brain tumour treatment." Expert Opinion in Biological Therapy **4**(9): 1453-71, 2004 Sep.

Bourquin, C., A. Iglesias, et al. (2000). "Myelin oligodendrocyte glycoprotein-DNA vaccination induces antibody-mediated autoaggression in experimental autoimmune encephalomyelitis." European Journal of Immunology **30**(12): 3663-71.

Braun, D. P., R. D. Penn, et al. (1982). "Immunoregulatory cell function in peripheral blood leukocytes of patients with intracranial gliomas." Neurosurgery **10**(2): 203-209.

Brooks, W. H., H. D. Caldwell, et al. (1974). "Immune responses in patients with gliomas." Surgical Neurology **2**(6): 419-423.

Brooks, W. H., M. G. Netsky, et al. (1972). "Depressed cell-mediated immunity in patients with primary intracranial tumors. Characterization of a humoral immunosuppressive factor." Journal of Experimental Medicine **136**(6): 1631-1647.

Brooks, W. H., T. L. Roszman, et al. (1977). "Immunobiology of primary intracranial tumours. II. Analysis of lymphocyte subpopulations in patients with primary brain tumours." Clinical and Experimental Immunology **29**(1): 61-66.

Brooks, W. H., T. L. Roszman, et al. (1976). "Impairment of rosette-forming T lymphoctyes in patients with primary intracranial tumors." Cancer **37**(4): 1869-1873.

Bullard, D. E., D. G. Thomas, et al. (1985). "A preliminary study utilizing viable HLA mismatched cultured glioma cells as adjuvant therapy for patients with malignant gliomas." British Journal of Cancer **51**(2): 283-289.

Calzascia, T., W. Di Berardino-Besson, et al. (2003). "Cutting edge: cross-presentation as a mechanism for efficient recruitment of tumor-specific CTL to the brain." J Immunol **171**(5): 2187-91.

Cho, B. K., V. P. Rao, et al. (2000). "Homeostasis-stimulated proliferation drives naive T cells to differentiate directly into memory T cells.[see comment]." Journal of Experimental Medicine **192**(4): 549-56.

Chong, G. and M. A. Morse (2005). "Combining cancer vaccines with chemotherapy." Expert Opin Pharmacother **6**(16): 2813-20.

Chu, C. T., K. D. Everiss, et al. (1997). "Receptor dimerization is not a factor in the signalling activity of a transforming variant epidermal growth factor receptor (EGFRvIII)." Biochemical Journal **324**(Pt 3): 855-61.

Chu, C. T., K. D. Everiss, et al. (1997). "Receptor dimerization is not a factor in the signalling activity of a transforming variant epidermal growth factor receptor (EGFRvIII)." Biochemical Journal **324**(Pt 3): 855-861.

Cohen, M. H., J. R. Johnson, et al. (2005). "Food and Drug Administration Drug approval summary: temozolomide plus radiation therapy for the treatment of newly diagnosed glioblastoma multiforme." Clin Cancer Res **11**(19 Pt 1): 6767-71.

Correale, P., M. G. Cusi, et al. (2005). "Dendritic cell-mediated cross-presentation of antigens derived from colon carcinoma cells exposed to a highly cytotoxic multidrug regimen with gemcitabine, oxaliplatin, 5-fluorouracil, and leucovorin, elicits a powerful human antigen-specific CTL response with antitumor activity in vitro." J Immunol **175**(2): 820-8.

Cox, D. R. (1972). "Regression models and life-tables (with discussion)." Journal of the Royal Statistical Society **34**: 187-220.

Cunningham, M. P., S. Essapen, et al. (2005). "Coexpression, prognostic significance and predictive value of EGFR, EGFRvIII and phosphorylated EGFR in colorectal cancer." International Journal of Oncology **27**(2): 317-25.

Curiel, T. J., G. Coukos, et al. (2004). "Specific recruitment of regulatory T cells in ovarian carcinoma fosters immune privilege and predicts reduced survival." Nature Medicine **10**(9): 942-9.

Curran, W. J., Jr, C. B. Scott, et al. (1993). "Recursive partitioning analysis of prognostic factors in three Radiation Therapy Oncology Group malignant glioma trials." Journal of the National Cancer Institute **85**(9): 704-710.

Czarniecki, C. W., H. H. Chiu, et al. (1988). "Transforming growth factor-beta 1 modulates the expression of class II histocompatibility antigens on human cells." Journal of Immunology **140**(12): 4217-4223.

D'Cruz, L. M. and L. Klein (2005). "Development and function of agonist-induced CD25+Foxp3+ regulatory T cells in the absence of interleukin 2 signaling.[see comment]." Nature Immunology **6**(11): 1152-9.

Dannull, J., Z. Su, et al. (2005). "Enhancement of vaccine-mediated antitumor immunity in cancer patients after depletion of regulatory T cells." Journal of Clinical Investigation **115**(12): 3623-33.

de Roux, A., A. Marx, et al. (2006). "Impact of corticosteroids on the immune response to a MF59-adjuvanted influenza vaccine in elderly COPD-patients." Vaccine **24**(10): 1537-42.

de Roux, A., N. Schmidt, et al. (2004). "Immunogenity of the pneumococcal polysaccharide vaccine in COPD patients. The effect of systemic steroids." Respir Med **98**(12): 1187-94.

Depper, J. M., W. J. Leonard, et al. (1983). "Blockade of the interleukin-2 receptor by anti-Tac antibody: inhibition of human lymphocyte activation." Journal of Immunology **131**(2): 690-6.

Dieckmann, D., H. Plottner, et al. (2001). "Ex vivo isolation and characterization of CD4(+)CD25(+) T cells with regulatory properties from human blood." Journal of Experimental Medicine **193**(11): 1303-10.

Dinapoli, R. P., L. D. Brown, et al. (1993). "Phase III comparative evaluation of PCNU and carmustine combined with radiation therapy for high-grade glioma." Journal of Clinical Oncology **11**(7): 1316-1321.

Dittel, B. N., I. Visintin, et al. (1999). "Presentation of the self antigen myelin basic protein by dendritic cells leads to experimental autoimmune encephalomyelitis." Journal of Immunology **163**(1): 32-9.

Dudley, M. E., J. R. Wunderlich, et al. (2002). "Cancer regression and autoimmunity in patients after clonal repopulation with antitumor lymphocytes." Science **298**(5594): 850-4.

Dudley, M. E., J. R. Wunderlich, et al. (2005). "Adoptive cell transfer therapy following non-myeloablative but lymphodepleting chemotherapy for the treatment of patients with refractory metastatic melanoma." Journal of Clinical Oncology **23**: 2346-57, 2005 Apr 1.

Ekman, M. and M. Westphal (2005). "Cost of brain tumour in Europe." European Journal of Neurology **12**: 45-9.

Ekstrand, A. J., C. D. James, et al. (1991). "Genes for epidermal growth factor receptor, transforming growth factor alpha, and epidermal growth factor and their expression in human gliomas in vivo." Cancer Research **51**(8): 2164-2172.

Elliott, L., W. Brooks, et al. (1987). "Role of interleukin-2 (IL-2) and IL-2 receptor expression in the proliferative defect observed in mitogen-stimulated lymphocytes from patients with gliomas." Journal of the National Cancer Institute **78**(5): 919-922.

Elliott, L. H., W. H. Brooks, et al. (1990). "Inability of mitogen-activated lymphocytes obtained from patients with malignant primary intracranial tumors to express high affinity interleukin 2 receptors." Journal of Clinical Investigation **86**(1): 80-86.

Emens, L. A. and E. M. Jaffee (2005). "Leveraging the activity of tumor vaccines with cytotoxic chemotherapy." Cancer Res **65**(18): 8059-64.

Emens, L. A., J. P. Machiels, et al. (2001). "Chemotherapy: friend or foe to cancer vaccines?" Curr Opin Mol Ther **3**(1): 77-84.

Espevik, T., I. S. Figari, et al. (1987). "Inhibition of cytokine production by cyclosporin A and transforming growth factor beta." Journal of Experimental Medicine **166**(2): 571-576.

Fallarino, F., U. Grohmann, et al. (2003). "Modulation of tryptophan catabolism by regulatory T cells." Nature Immunology **4**(12): 1206-12.

Fecci, P. E., D. A. Mitchell, et al. (2006). "Increased regulatory T-cell fraction amidst a diminished CD4 compartment explains cellular immune defects in patients with malignant glioma." Cancer Res **66**(6): 3294-302.

Fecci, P. E., D. A. Mitchell, et al. (2006). "Increased regulatory T-cell fraction amidst a diminished CD4 compartment explains cellular immune defects in patients with malignant glioma." Cancer Research **66**(6): 3294-302.

Fecci, P. E., D. A. Mitchell, et al. (2006). "Increased regulatory T-cell fraction amidst a diminished CD4 compartment explains cellular immune defects in patients with malignant glioma." Cancer Research **66**(6): 3294-3302.

Fecci, P. E., H. Ochiai, et al. (2006). "Systemic CTLA-4 blockade ameliorates glioma-induced changes to the CD4+ T-cell compartment without affecting regulatory T-cell function." Journal of Experimental Medicine **Submitted**.

Fecci, P. E., A. E. Sweeney, et al. (2006). "Systemic Anti-CD25 Monoclonal Antibody Administration Safely Enhances Immunity in Murine Glioma without Eliminating Regulatory T Cells." Clinical Cancer Research **12**(14): 4294-4305.

Fecci, P. E., A. S. Sweeney, et al. (2006). "Systemic Treg depletion safely enhances immunity in murine glioma." Clinical Cancer Research **In press**.
Ferguson, T. A., J. Herndon, et al. (2002). "Uptake of apoptotic antigen-coupled cells by lymphoid dendritic cells and cross-priming of CD8(+) T cells produce active immune unresponsiveness." J Immunol **168**(11): 5589-95.
Fontana, A., H. Hengartner, et al. (1984). "Glioblastoma cells release interleukin 1 and factors inhibiting interleukin 2-mediated effects." Journal of Immunology **132**(4): 1837-1844.
Fontenot, J. D., M. A. Gavin, et al. (2003). "Foxp3 programs the development and function of CD4+CD25+ regulatory T cells." Nature Immunology **4**(4): 330-6.
Fontenot, J. D., J. P. Rasmussen, et al. (2005). "A function for interleukin 2 in Foxp3-expressing regulatory T cells." Nature Immunology **6**(11): 1142-51.
Fontenot, J. D., J. P. Rasmussen, et al. (2005). "A function for interleukin 2 in Foxp3-expressing regulatory T cells.[see comment][erratum appears in Nat Immunol. 2006 Apr;7(4):427]." Nature Immunology **6**(11): 1142-51.
Frederick, L., X. Y. Wang, et al. (2000). "Diversity and frequency of epidermal growth factor receptor mutations in human glioblastomas." Cancer Research **60**(5): 1383-7.
Galanis, E., J. C. Buckner, et al. (2006). "Validation of neuroradiologic response assessment in gliomas: measurement by RECIST, two-dimensional, computer-assisted tumor area, and computer-assisted tumor volume methods." Neuro-Oncology **8**(2): 156-65.
Garcia de Palazzo, I. E., G. P. Adams, et al. (1993). "Expression of mutated epidermal growth factor receptor by non- small cell lung carcinomas." Cancer Research **53**: 3217-3220.
Garcia de Palazzo, I. E., G. P. Adams, et al. (1993). "Expression of mutated epidermal growth factor receptor by non- small cell lung carcinomas." Cancer Res. **53**: 3217-3220.
Ge, H., X. Gong, et al. (2002). "Evidence of high incidence of EGFRvIII expression and coexpression with EGFR in human invasive breast cancer by laser capture microdissection and immunohistochemical analysis." International Journal of Cancer **98**(3): 357-61.
Gilboa, E. (1999). "The makings of a tumor rejection antigen." Immunity **11**(3): 263-70.
Gilboa, E. (2004). "The promise of cancer vaccines." Nature Reviews: Cancer **4**(5): 401-11.
Goebel, J., E. Stevens, et al. (2000). "Daclizumab (Zenapax) inhibits early interleukin-2 receptor signal transduction events." Transplant Immunology **8**(3): 153-9.
Green, D. R. and D. R. Webb (1993). "Saying the 'S' word in public." Immunology Today **14**(11): 523-5.
Gri, G., C. Chiodoni, et al. (2002). "Antitumor effect of interleukin (IL)-12 in the absence of endogenous IFN-gamma: a role for intrinsic tumor immunogenicity and IL-15." Cancer Res **62**(15): 4390-7.
Grossman, Z. and W. E. Paul (2000). "Self-tolerance: context dependent tuning of T cell antigen recognition." Seminars in Immunology **12**(3): 197-203; discussion 257-344.

Haeryfar, S. M., R. J. DiPaolo, et al. (2005). "Regulatory T cells suppress CD8+ T cell responses induced by direct priming and cross-priming and moderate immunodominance disparities." J Immunol **174**(6): 3344-51.

Hall, W. A. and O. Fodstad (1992). "Immunotoxins and central nervous system neoplasia." Journal of Neurosurgery **76**(1): 1-12.

Harris, J. R. and J. Markl (1999). "Keyhole limpet hemocyanin (KLH): a biomedical review." Micron **30**(6): 597-623.

Hegi, M. E., A. C. Diserens, et al. (2005). "MGMT gene silencing and benefit from temozolomide in glioblastoma." New England Journal of Medicine **352**: 997-1003, 2005 Mar 10.

Heimberger, A., W. Sun, et al. (2007). "Immunological responses in a patient with glioblastoma multiforme treated with sequential courses of temozolomide and immunotherapy: Case study." Neuro-oncology **In press**.

Heimberger, A. B., G. E. Archer, et al. (2002). "Dendritic cells pulsed with a tumor-specific peptide induce long-lasting immunity and are effective against murine intracerebral melanoma." Neurosurgery **50**(1): 158-64; discussion 164-6.

Heimberger, A. B., G. E. Archer, et al. (2002). "Dendritic cells pulsed with a tumor-specific peptide induce long-lasting immunity and are effective against murine intracerebral melanoma." Neurosurgery **50**: 158-166.

Heimberger, A. B., L. E. Crotty, et al. (2003). "Epidermal growth factor receptor VIII peptide vaccination is efficacious against established intracerebral tumors." Clin Cancer Res **9**(11): 4247-54.

Heimberger, A. B., L. E. Crotty, et al. (2000). "Bone marrow-derived dendritic cells pulsed with tumor homogenate induce immunity against syngeneic intracerebral glioma." Journal of Neuroimmunology **103**(1): 16-25.

Heimberger, A. B., L. E. Crotty, et al. (2000). "Bone marrow-derived dendritic cells pulsed with tumor homogenate induce immunity against syngeneic intracerebral glioma." J Neuroimmunol **103**(1): 16-25.

Heimberger, A. B., R. Hlatky, et al. (2005). "Prognostic effect of epidermal growth factor receptor and EGFRvIII in glioblastoma multiforme patients." Clinical Cancer Research **11**(4): 1462-6.

Heimberger, A. B., S. F. Hussain, et al. (2006). Tumor-specific peptide vaccination in newly-diagnosed patients with GBM. American Society of Clinical Oncology Annual Meeting, Atlanta, GA.

Heimberger, A. B., S. F. Hussain, et al. (2006). "Tumor-specific peptide vaccination in newly-diagnosed patients with GBM." J Clin Oncol **24**(18S): 107s abstract 2529.

Heimberger, A. B., S. F. Hussain, et al. (2006). An Epidermal Growth Factor Receptor Variant III Peptide Vaccination Appears Promising in Newly Diagnosed GBM Patients: Preliminary Results of a Randomized Phase II Clinical Trial American Association of Neurological Surgeons. San Francisco, CA.

Hoffmann, T. K., N. Meidenbauer, et al. (2000). "Generation of tumor-specific T-lymphocytes by cross-priming with human dendritic cells ingesting apoptotic tumor cells." Cancer Res **60**(13): 3542-9.

Hoffmann, T. K., N. Meidenbauer, et al. (2001). "Proinflammatory Cytokines and CD40 Ligand Enhance Cross-Presentation and Cross-Priming Capability of Human

Dendritic Cells Internalizing Apoptotic Cancer Cells." J Immunother **24**(2): 162-171.
Homma, S., Y. Sagawa, et al. (2006). "Cancer immunotherapy using dendritic/tumour-fusion vaccine induces elevation of serum anti-nuclear antibody with better clinical responses." Clinical & Experimental Immunology **144**(1): 41-7.
Hu, H. M., C. H. Poehlein, et al. (2002). "Development of antitumor immune responses in reconstituted lymphopenic hosts." Cancer Research **62**(14): 3914-9.
Huang, A. Y., P. Golumbek, et al. (1994). "Bone marrow-derived cells present MHC class I-restricted tumour antigens in priming of antitumour immune responses." Ciba Found Symp **187**: 229-40; discussion 240-4.
Huang, H. S., M. Nagane, et al. (1997). "The enhanced tumorigenic activity of a mutant epidermal growth factor receptor common in human cancers is mediated by threshold levels of constitutive tyrosine phosphorylation and unattenuated signaling." Journal of Biological Chemistry **272**(5): 2927-2935.
Hughes, M. S., Y. Y. Yu, et al. (2005). "Transfer of a TCR gene derived from a patient with a marked antitumor response conveys highly active T-cell effector functions." Human Gene Therapy **16**: 457-72, 2005 Apr.
Humphrey, P. A., A. J. Wong, et al. (1990). "Anti-synthetic peptide antibody reacting at the fusion junction of deletion-mutant epidermal growth factor receptors in human glioblastoma." Proceedings of the National Academy of Sciences of the United States of America **87**(11): 4207-4211.
Imperato, J. P., N. A. Paleologos, et al. (1990). "Effects of treatment on long-term survivors with malignant astrocytomas." Annals of Neurology **28**(6): 818-822.
Jaber, S. H., E. W. Cowen, et al. (2006). "Skin reactions in a subset of patients with stage IV melanoma treated with anti-cytotoxic T-lymphocyte antigen 4 monoclonal antibody as a single agent." Archives of Dermatology **142**(2): 166-72.
Jonuleit, H., E. Schmitt, et al. (2001). "Identification and functional characterization of human CD4(+)CD25(+) T cells with regulatory properties isolated from peripheral blood." Journal of Experimental Medicine **193**(11): 1285-94.
Kabat, E. A., A. Wolf, et al. (1947). "The rapid production of acute disseminated encephalomyelitis in rhesus monkeys by injection of heterologous and homologous brain tissue with adjuvants." Journal of Experimental Medicine **85**: 117-130.
Kammerer, R., D. Stober, et al. (2002). "Noncovalent association with stress protein facilitates cross-priming of CD8+ T cells to tumor cell antigens by dendritic cells." J Immunol **168**(1): 108-17.
Kapetanovic, M. C., T. Saxne, et al. (2006). "Influence of methotrexate, TNF blockers and prednisolone on antibody responses to pneumococcal polysaccharide vaccine in patients with rheumatoid arthritis." Rheumatology (Oxford) **45**(1): 106-11.
Kaplan, E. L. and P. Meier (1958). "Nonparametric estimation from incomplete observations." Journal of the American Statistical Association **53**: 457-481.
Kehrl, J. H., A. B. Roberts, et al. (1986). "Transforming growth factor beta is an important immunomodulatory protein for human B lymphocytes." Journal of Immunology **137**(12): 3855-3860.

Kehrl, J. H., L. M. Wakefield, et al. (1986). "Production of transforming growth factor beta by human T lymphocytes and its potential role in the regulation of T cell growth." Journal of Experimental Medicine **163**(5): 1037-1050.

Kelly, P. J. (1992). "Stereotactic resection and its limitations in glial neoplasms." Stereotactic and Functional Neurosurgery **59**(1-4): 84-91.

Khattri, R., T. Cox, et al. (2003). "An essential role for Scurfin in CD4+CD25+ T regulatory cells." Nature Immunology **4**(4): 337-42.

Kikuchi, T., Y. Akasaki, et al. (2004). "Vaccination of glioma patients with fusions of dendritic and glioma cells and recombinant human interleukin 12." Journal of Immunotherapy with Emphasis on Tumor Immunology **27**: 452-9.

Kohm, A. P., P. A. Carpentier, et al. (2002). "Cutting edge: CD4+CD25+ regulatory T cells suppress antigen-specific autoreactive immune responses and central nervous system inflammation during active experimental autoimmune encephalomyelitis." Journal of Immunology **169**(9): 4712-6.

Kohm, A. P., J. S. McMahon, et al. (2006). "Cutting Edge: Anti-CD25 monoclonal antibody injection results in the functional inactivation, not depletion, of CD4+CD25+ T regulatory cells." Journal of Immunology **176**(6): 3301-5.

Kotliarov, Y., M. E. Steed, et al. (2006). "High-resolution Global Genomic Survey of 178 Gliomas Reveals Novel Regions of Copy Number Alteration and Allelic Imbalances." Cancer Research **66**(19): 9428-36.

Kremer, I. B., S. R. Stevens, et al. (2000). "Intradermal granulocyte-macrophage colony-stimulating factor alters cutaneous antigen-presenting cells and differentially affects local versus distant immunization in humans." Clinical Immunology **96**(1): 29-37.

Lamborn, K. R., S. M. Chang, et al. (2004). "Prognostic factors for survival of patients with glioblastoma: recursive partitioning analysis." Neuro-oncology **6**: 227-35, 2004 Jul.

Lammering, G., T. H. Hewit, et al. (2004). "Inhibition of the type III epidermal growth factor receptor variant mutant receptor by dominant-negative EGFR-CD533 enhances malignant glioma cell radiosensitivity." Clinical Cancer Research **10**(19): 6732-43.

Lammering, G., T. H. Hewit, et al. (2003). "EGFRvIII-mediated radioresistance through a strong cytoprotective response." Oncogene **22**(36): 5545-53.

Lammering, G., K. Valerie, et al. (2004). "Radiation-induced activation of a common variant of EGFR confers enhanced radioresistance." Radiotherapy & Oncology **72**(3): 267-73.

Lengauer, C., K. W. Kinzler, et al. (1998). "Genetic instabilities in human cancers." Nature **396**(6712): 643-9.

Lennerz, V., M. Fatho, et al. (2005). "The response of autologous T cells to a human melanoma is dominated by mutated neoantigens." Proceedings of the National Academy of Sciences of the United States of America **102**(44): 16013-8.

Li, B., M. Yuan, et al. (2004). "Mutant epidermal growth factor receptor displays increased signaling through the phosphatidylinositol-3 kinase/AKT pathway and promotes radioresistance in cells of astrocytic origin." Oncogene **23**(26): 4594-602.

Li, Z., W. K. Lim, et al. (2005). "Cutting edge: in vivo blockade of human IL-2 receptor induces expansion of CD56(bright) regulatory NK cells in patients with active uveitis.[erratum appears in J Immunol. 2005 Sep 1;175(5):3447]." Journal of Immunology **174**(9): 5187-91.

Liau, L., K. L. Black, et al. (1999). "Treatment of intracranial gliomas with bone marrow-derived dendritic cells pulsed with tumor antigens." Journal of Neurosurgery **90**(6): 1115-1124.

Liau, L. M., R. M. Prins, et al. (2005). "Dendritic cell vaccination in glioblastoma patients induces systemic and intracranial T-cell responses modulated by the local central nervous system tumor microenvironment." Clinical Cancer Research **11**: 5515-25.

Libermann, T. A., H. R. Nusbaum, et al. (1985). "Amplification, enhanced expression and possible rearrangement of EGF receptor gene in primary human brain tumours of glial origin." Nature **313**(5998): 144-147.

Lim, H. W., P. Hillsamer, et al. (2005). "Cutting edge: direct suppression of B cells by CD4+ CD25+ regulatory T cells." Journal of Immunology **175**(7): 4180-3.

Linington, C., T. Berger, et al. (1993). "T cells specific for the myelin oligodendrocyte glycoprotein mediate an unusual autoimmune inflammatory response in the central nervous system." European Journal of Immunology **23**(6): 1364-1372.

Lodge, P. A., L. A. Jones, et al. (2000). "Dendritic cell-based immunotherapy of prostate cancer: immune monitoring of a phase II clinical trial." Cancer Research **60**(4): 829-33.

Loeb, L. A. (2001). "A mutator phenotype in cancer." Cancer Research **61**(8): 3230-9.

Ludewig, B., A. F. Ochsenbein, et al. (2000). "Immunotherapy with dendritic cells directed against tumor antigens shared with normal host cells results in severe autoimmune disease." Journal of Experimental Medicine **191**(5): 795-804.

Luo, X., X. Gong, et al. (2003). "Suppression of EGFRvIII-mediated proliferation and tumorigenesis of breast cancer cells by ribozyme." International Journal of Cancer **104**(6): 716-21.

Luwor, R. B., T. G. Johns, et al. (2001). "Monoclonal antibody 806 inhibits the growth of tumor xenografts expressing either the de2-7 or amplified epidermal growth factor receptor (EGFR) but not wild-type EGFR." Cancer Res **61**(14): 5355-61.

Macdonald, D. R., T. L. Cascino, et al. (1990). "Response criteria for phase II studies of supratentorial malignant glioma." Journal of Clinical Oncology **8**(7): 1277-1280.

Mach, N., S. Gillessen, et al. (2000). "Differences in dendritic cells stimulated in vivo by tumors engineered to secrete granulocyte-macrophage colony-stimulating factor or Flt3-ligand." Cancer Research **60**(12): 3239-46.

Machiels, J. P., R. T. Reilly, et al. (2001). "Cyclophosphamide, doxorubicin, and paclitaxel enhance the antitumor immune response of granulocyte/macrophage-colony stimulating factor-secreting whole-cell vaccines in HER-2/neu tolerized mice." Cancer Res **61**(9): 3689-97.

Mackensen, A., B. Herbst, et al. (2000). "Phase I study in melanoma patients of a vaccine with peptide-pulsed dendritic cells generated in vitro from CD34(+) hematopoietic progenitor cells." International Journal of Cancer **86**(3): 385-92.

Mahaley, M. S., Jr., D. D. Bigner, et al. (1983). "Immunobiology of primary intracranial tumors. Part 7: Active immunization of patients with anaplastic human glioma cells: a pilot study." Journal of Neurosurgery **59**(2): 201-207.

Mahaley, M. S., Jr., W. H. Brooks, et al. (1977). "Immunobiology of primary intracranial tumors. Part 1: studies of the cellular and humoral general immune competence of brain-tumor patients." Journal of Neurosurgery **46**(4): 467-476.

Malek, T. R. (2003). "The main function of IL-2 is to promote the development of T regulatory cells." Journal of Leukocyte Biology **74**(6): 961-5.

Malek, T. R. and A. L. Bayer (2004). "Tolerance, not immunity, crucially depends on IL-2." Nature Reviews Immunology **4**(9): 665-74.

Maloy, K. J. and F. Powrie (2005). "Fueling regulation: IL-2 keeps CD4+ Treg cells fit.[comment]." Nature Immunology **6**(11): 1071-2.

McHugh, R. S. and E. M. Shevach (2002). "Cutting edge: Depletion of CD4+CD25+ regulatory T cells is necessary, but not sufficient, for induction of organ-specific autoimmune disease." Journal of Immunology. **168**(12): 5979-83.

Mellinghoff, I. K., M. Y. Wang, et al. (2005). "Molecular determinants of the response of glioblastomas to EGFR kinase inhibitors." New England Journal of Medicine **353**(19): 2012-24.

Menzies, C. B., M. Gunar, et al. (1980). "Impaired thymus-derived lymphocyte function in patients with malignant brain tumour." Clinical Neurology and Neurosurgery **82**(3): 157-168.

Miescher, S., T. L. Whiteside, et al. (1986). "Functional properties of tumor-infiltrating and blood lymphocytes in patients with solid tumors: effects of tumor cells and their supernatants on proliferative responses of lymphocytes." Journal of Immunology **136**(5): 1899-1907.

Miescher, S., T. L. Whiteside, et al. (1988). "In situ characterization, clonogenic potential, and antitumor cytolytic activity of T lymphocytes infiltrating human brain cancers." Journal of Neurosurgery **68**(3): 438-448.

Miyagi, Y., N. Imai, et al. (2001). "Induction of cellular immune responses to tumor cells and peptides in colorectal cancer patients by vaccination with SART3 peptides." Clinical Cancer Research **7**(12): 3950-62.

Montgomery, R. B., J. Guzman, et al. (2000). "Expression of oncogenic epidermal growth factor receptor family kinases induces paclitaxel resistance and alters beta-tubulin isotype expression." Journal of Biological Chemistry **275**(23): 17358-63.

Moscatello, D. K., M. Holgado-Madruga, et al. (1995). "Frequent expression of a mutant epidermal growth factor receptor in multiple human tumors." Cancer Research **55**(23): 5536-5539.

Moscatello, D. K., R. B. Montgomery, et al. (1996). "Transformation and altered signal transduction by a naturally occurring mutant EGF receptor." Oncogene **13**: 85-96.

Moscatello, D. K., G. Ramirez, et al. (1997). "A naturally occurring mutant human epidermal growth factor receptor as a target for peptide vaccine immunotherapy of tumors." Cancer Res **57**(8): 1419-24.

Nagane, M., F. Coufal, et al. (1996). "A common mutant epidermal growth factor receptor confers enhanced tumorigenicity on human glioblastoma cells by increasing proliferation and reducing apoptosis." Cancer Research **56**(21): 5079-5086.

Nagane, M., Y. Narita, et al. (2001). "Human glioblastoma xenografts overexpressing a tumor-specific mutant epidermal growth factor receptor sensitized to cisplatin by the AG1478 tyrosine kinase inhibitor." Journal of Neurosurgery **95**(3): 472-9.

Ng, W. F., P. J. Duggan, et al. (2001). "Human CD4(+)CD25(+) cells: a naturally occurring population of regulatory T cells." Blood **98**(9): 2736-44.

Nishikawa, R., X. D. Ji, et al. (1994). "A mutant epidermal growth factor receptor common in human glioma confers enhanced tumorigenicity." Proceedings of the National Academy of Sciences of the United States of America **91**(16): 7727-7731.

Nishimura, H., M. Tagaya, et al. (2001). "Mice lacking interleukin-2 (IL-2)/IL-15 receptor beta chain are susceptible to infection with avirulent Salmonella enterica subsp. enterica serovar choleraesuis but mice lacking IL-2 are resistant." Infection & Immunity **69**(2): 1226-9.

Nowak, A. K., R. A. Lake, et al. (2003). "Induction of tumor cell apoptosis in vivo increases tumor antigen cross-presentation, cross-priming rather than cross-tolerizing host tumor-specific CD8 T cells." J Immunol **170**(10): 4905-13.

Ochsenbein, A. F., P. Klenerman, et al. (1999). "Immune surveillance against a solid tumor fails because of immunological ignorance." Proceedings of the National Academy of Sciences of the United States of America **96**(5): 2233-8.

Okamoto, I., L. C. Kenyon, et al. (2003). "Expression of constitutively activated EGFRvIII in non-small cell lung cancer." Cancer Science **94**(1): 50-6.

Olapade-Olaopa, E. O., D. K. Moscatello, et al. (2000). "Evidence for the differential expression of a variant EGF receptor protein in human prostate cancer." British Journal of Cancer **82**(1): 186-94.

Ommaya, A. K. (1976). "Immunotherapy of gliomas: a review." Advances in Neurology **15**: 337-359.

Otahal, P., S. C. Hutchinson, et al. (2005). "Inefficient cross-presentation limits the CD8+ T cell response to a subdominant tumor antigen epitope." J Immunol **175**(2): 700-12.

Pasteur, L. (1885). "Methode pour prevenir la rage apres morsure." Comptes rendus de l'academie des sciences (Paris) **101**: 765-774.

Pedersen, M. W., V. Tkach, et al. (2004). "Expression of a naturally occurring constitutively active variant of the epidermal growth factor receptor in mouse fibroblasts increases motility." International Journal of Cancer **108**(5): 643-53.

Phan, G. Q., J. C. Yang, et al. (2003). "Cancer regression and autoimmunity induced by cytotoxic T lymphocyte-associated antigen 4 blockade in patients with metastatic melanoma." Proceedings of the National Academy of Sciences of the United States of America **100**(14): 8372-7.

Pickard, J. D., S. Bailey, et al. (1990). "Steps towards cost-benefit analysis of regional neurosurgical care." British Medical Journal **301**(6753): 629-635.

Plautz, G. E., S. Mukai, et al. (2000). "Cross-presentation of tumor antigens to effector T cells is sufficient to mediate effective immunotherapy of established intracranial tumors." J Immunol **165**(7): 3656-62.

Purev, E., D. Cai, et al. (2004). "Immune responses of breast cancer patients to mutated epidermal growth factor receptor (EGF-RvIII, Delta EGF-R, and de2-7 EGF-R)." J Immunol **173**(10): 6472-80.

Queen, C., W. P. Schneider, et al. (1989). "A humanized antibody that binds to the interleukin 2 receptor." Proceedings of the National Academy of Sciences of the United States of America **86**(24): 10029-33.
Ralainirina, N., A. Poli, et al. (2007). "Control of natural killer (NK) cell functions by CD4+CD25+ regulatory T cells." Journal of Leukocyte Biology **81**: In press.
Ranges, G. E., I. S. Figari, et al. (1987). "Inhibition of cytotoxic T cell development by transforming growth factor beta and reversal by recombinant tumor necrosis factor alpha." Journal of Experimental Medicine **166**(4): 991-998.
Remlinger, P. (1904). "Contribution a l'etude de la toxine rabique (faits experimentaux et clinique)." Comptes rendus des seances de la Societe de Biologie **56**: 348-350.
Remlinger, P. (1905). "Accidents paralytiques au cours du traitement antirabique." Annales de l'Institut Pasteur **19**: 625-646.
Rivers, T. M. and F. F. Schwentker (1935). "Encephalomyelitis accompanied by myelin destruction experimentally produced in monkeys." Journal of Experimental Medicine **61**: 689-702.
Rook, A. H., J. H. Kehrl, et al. (1986). "Effects of transforming growth factor beta on the functions of natural killer cells: depressed cytolytic activity and blunting of interferon responsiveness." Journal of Immunology **136**(10): 3916-3920.
Rosenberg, S. A., J. C. Yang, et al. (1998). "Immunologic and therapeutic evaluation of a synthetic peptide vaccine for the treatment of patients with metastatic melanoma [see comments]." Nature Medicine **4**(3): 321-327.
Roszman, T. L. and W. H. Brooks (1980). "Immunobiology of primary intracranial tumours. III. Demonstration of a qualitative lymphocyte abnormality in patients with primary brain tumours." Clinical and Experimental Immunology **39**(2): 395-402.
Roszman, T. L., W. H. Brooks, et al. (1982). "Immunobiology of primary intracranial tumors. VI. Suppressor cell function and lectin-binding lymphocyte subpopulations in patients with cerebral tumors." Cancer **50**(7): 1273-1279.
Roszman, T. L., W. H. Brooks, et al. (1987). "Inhibition of lymphocyte responsiveness by a glial tumor cell- derived suppressive factor." Journal of Neurosurgery **67**(6): 874-879.
Roszman, T. L., L. H. Elliott, et al. (1992). "Proliferative potential of T-cell lymphocytes from gliomas." Journal of Neurosurgery **77**(5): 820-821.
Rovere-Querini, P. and I. E. Dumitriu (2003). "Corpse disposal after apoptosis." Apoptosis **8**(5): 469-79.
Sakaguchi, S., N. Sakaguchi, et al. (1995). "Immunologic self-tolerance maintained by activated T cells expressing IL-2 receptor alpha-chains (CD25). Breakdown of a single mechanism of self-tolerance causes various autoimmune diseases." Journal of Immunology **155**(3): 1151-64.
Salford, L. G., A. Brun, et al. (1988). "Ten-year survival among patients with supratentorial astrocytomas grade III and IV." Journal of Neurosurgery **69**(4): 506-509.
Salomon, B., D. J. Lenschow, et al. (2000). "B7/CD28 costimulation is essential for the homeostasis of the CD4+CD25+ immunoregulatory T cells that control autoimmune diabetes." Immunity. **12**(4): 431-40.

Sampson, J. H., D. M. Ashley, et al. (1997). "Characterization of a spontaneous murine astrocytoma and abrogation of its tumorigenicity by cytokine secretion." Neurosurgery **41**(6): 1365-1373.
Sampson, J. H., L. E. Crotty, et al. (2000). "Unarmed, tumor-specific monoclonal antibody effectively treats brain tumors." Proc Natl Acad Sci U S A **97**(13): 7503-8.
Scheffer, S. R., H. Nave, et al. (2003). "Apoptotic, but not necrotic, tumor cell vaccines induce a potent immune response in vivo." Int J Cancer **103**(2): 205-11.
Schnurr, M., C. Scholz, et al. (2002). "Apoptotic pancreatic tumor cells are superior to cell lysates in promoting cross-priming of cytotoxic T cells and activate NK and gammadelta T cells." Cancer Res **62**(8): 2347-52.
Schwyzer, M. and A. Fontana (1985). "Partial purification and biochemical characterization of a T cell suppressor factor produced by human glioblastoma cells." Journal of Immunology **134**(2): 1003-1009.
Seddon, B. and D. Mason (1999). "Peripheral autoantigen induces regulatory T cells that prevent autoimmunity." Journal of Experimental Medicine **189**(5): 877-82.
Seddon, B. and D. Mason (1999). "Regulatory T cells in the control of autoimmunity: the essential role of transforming growth factor beta and interleukin 4 in the prevention of autoimmune thyroiditis in rats by peripheral CD4(+)CD45RC- cells and CD4(+)CD8(-) thymocytes." Journal of Experimental Medicine **189**(2): 279-88.
Seddon, B., P. Tomlinson, et al. (2003). "Interleukin 7 and T cell receptor signals regulate homeostasis of CD4 memory cells." Nature Immunology **4**(7): 680-6.
Seddon, B. and R. Zamoyska (2003). "Regulation of peripheral T-cell homeostasis by receptor signalling." Current Opinion in Immunology **15**(3): 321-4.
Sempowski, G. D., C. B. Hicks, et al. (2005). "Naive T cells are maintained in the periphery during the first 3 months of acute HIV-1 infection: implications for analysis of thymus function." Journal of Clinical Immunology **25**(5): 462-72.
Shapiro, W. R. (1986). "Therapy of adult malignant brain tumors: what have the clinical trials taught us?." Seminars in Oncology **13**(1): 38-45.
Shi, H., T. Cao, et al. (2006). "Hyperthermia enhances CTL cross-priming." J Immunol **176**(4): 2134-41.
Shimizu, J., S. Yamazaki, et al. (1999). "Induction of tumor immunity by removing CD25+CD4+ T cells: a common basis between tumor immunity and autoimmunity." Journal of Immunology. **163**(10): 5211-8.
Silva, H. A. C., E. Abraul, et al. (2006). "Molecular detection of EGFRvIII-positive cells in the peripheral blood of breast cancer patients." European Journal of Cancer **42**(15): 2617-22.
Siris, J. H. (1936). "Concerning the immunological specificity of glioblastoma multiforme." Bulletin of Neurology of New York **4**: 597-601.
Slagel, D. E., C. B. Wilson, et al. (1969). "Polyacrylamide electrophoresis and immunodiffusion studies of brain tumor proteins." Annals of the New York Academy of Sciences **159**: 490-496.
Smyth, M. J., M. W. Teng, et al. (2006). "CD4+CD25+ T regulatory cells suppress NK cell-mediated immunotherapy of cancer." Journal of Immunology **176**(3): 1582-7.

Soiffer, R., T. Lynch, et al. (1998). "Vaccination with irradiated autologous melanoma cells engineered to secrete human granulocyte-macrophage colony-stimulating factor generates potent antitumor immunity in patients with metastatic melanoma." Proceedings of the National Academy of Sciences of the United States of America **95**(22): 13141-6.

Somasundaram, R., L. Jacob, et al. (2002). "Inhibition of cytolytic T lymphocyte proliferation by autologous CD4+/CD25+ regulatory T cells in a colorectal carcinoma patient is mediated by transforming growth factor-beta." Cancer Research **62**(18): 5267-72.

Sotomayor, E. M., I. Borrello, et al. (2001). "Cross-presentation of tumor antigens by bone marrow-derived antigen-presenting cells is the dominant mechanism in the induction of T-cell tolerance during B-cell lymphoma progression." Blood **98**(4): 1070-7.

Steitz, J., J. Bruck, et al. (2001). "Depletion of CD25(+) CD4(+) T cells and treatment with tyrosinase-related protein 2-transduced dendritic cells enhance the interferon alpha-induced, CD8(+) T-cell-dependent immune defense of B16 melanoma." Cancer Research. **61**(24): 8643-6.

Stephens, L. A., S. M. Anderton, et al. (2006). "Comment on "Cutting edge: anti-CD25 monoclonal antibody injection results in the functional inactivation, not depletion, of CD4+CD25+ T regulatory cells".[comment]." Journal of Immunology **177**(4): 2036; author reply 2037-8.

Stephens, L. A. and D. Mason (2000). "CD25 is a marker for CD4+ thymocytes that prevent autoimmune diabetes in rats, but peripheral T cells with this function are found in both CD25+ and CD25- subpopulations." Journal of Immunology. **165**(6): 3105-10.

Stuart, G. and K. Krikorian (1928). "The neuro-paralytic accidents of anti-rabies treatment." Annals of Tropical Medicine **22**: 327-377.

Stuart, G. and K. Krikorian (1930). "A fatal neuro-paralytic accident of anti-rabies treatment." Lancet **1**: 1123-1125.

Stupp, R., P. Y. Dietrich, et al. (2002). "Promising survival for patients with newly diagnosed glioblastoma multiforme treated with concomitant radiation plus temozolomide followed by adjuvant temozolomide." Journal of Clinical Oncology. **20**(5): 1375-82.

Stupp, R., W. P. Mason, et al. (2005). "Radiotherapy plus concomitant and adjuvant temozolomide for glioblastoma." N Engl J Med **352**(10): 987-96.

Stupp, R., W. P. Mason, et al. (2005). "Radiotherapy plus Concomitant and Adjuvant Temozolomide for Glioblastoma." New England Journal of Medicine **352**(10): 987-996.

Su, Y. B., S. Sohn, et al. (2004). "Selective CD4+ lymphopenia in melanoma patients treated with temozolomide: a toxicity with therapeutic implications." Journal of Clinical Oncology **22**(4): 610-6.

Su, Y. B., S. Sohn, et al. (2004). "Selective CD4+ lymphopenia in melanoma patients treated with temozolomide: a toxicity with therapeutic implications.[see comment][erratum appears in J Clin Oncol. 2004 May 15;22(10):2038]." Journal of Clinical Oncology **22**(4): 610-6.

Sugawa, N., A. J. Ekstrand, et al. (1990). "Identical splicing of aberrant epidermal growth factor receptor transcripts from amplified rearranged genes in human glioblastomas." Proceedings of the National Academy of Sciences of the United States of America **87**(21): 8602-8606.

Suri-Payer, E., A. Z. Amar, et al. (1998). "CD4+CD25+ T cells inhibit both the induction and effector function of autoreactive T cells and represent a unique lineage of immunoregulatory cells." Journal of Immunology **160**(3): 1212-8.

Sutmuller, R. P., L. M. van Duivenvoorde, et al. (2001). "Synergism of cytotoxic T lymphocyte-associated antigen 4 blockade and depletion of CD25(+) regulatory T cells in antitumor therapy reveals alternative pathways for suppression of autoreactive cytotoxic T lymphocyte responses." Journal of Experimental Medicine. **194**(6): 823-32.

Taams, L. S., J. M. van Amelsfort, et al. (2005). "Modulation of monocyte/macrophage function by human CD4+CD25+ regulatory T cells." Human Immunology **66**(3): 222-30.

Taguchi, O., K. Kontani, et al. (1994). "Tissue-specific suppressor T cells involved in self-tolerance are activated extrathymically by self-antigens." Immunology **82**(3): 365-9.

Taguchi, O. and Y. Nishizuka (1987). "Self tolerance and localized autoimmunity. Mouse models of autoimmune disease that suggest tissue-specific suppressor T cells are involved in self tolerance." Journal of Experimental Medicine **165**(1): 146-56.

Tanchot, C., M. M. Rosado, et al. (1997). "Lymphocyte homeostasis." Seminars in Immunology **9**(6): 331-7.

Tang, C. K., X. Q. Gong, et al. (2000). "Epidermal growth factor receptor vIII enhances tumorigenicity in human breast cancer." Cancer Res **60**(11): 3081-7.

Tatsumi, T., J. Huang, et al. (2003). "Intratumoral Delivery of Dendritic Cells Engineered to Secrete Both Interleukin (IL)-12 and IL-18 Effectively Treats Local and Distant Disease in Association with Broadly Reactive Tc1-type Immunity." Cancer Res **63**(19): 6378-6386.

Thomas, D. G., C. B. Lannigan, et al. (1975). "Letter: Impaired cell-mediated immunity in human brain tumours." Lancet **1**(7921): 1389-1390.

Thornton, A. M. and E. M. Shevach (1998). "CD4+CD25+ immunoregulatory T cells suppress polyclonal T cell activation in vitro by inhibiting interleukin 2 production." Journal of Experimental Medicine **188**(2): 287-96.

Tjoa, B. A., S. J. Simmons, et al. (1998). "Evaluation of phase I/II clinical trials in prostate cancer with dendritic cells and PSMA peptides." Prostate **36**(1): 39-44.

Tkaczuk, J., E. Milford, et al. (2001). "Intracellular signaling consequences of anti-IL-2Ralpha blockade by daclizumab." Transplantation Proceedings **33**(1-2): 212-3.

Trouillas, P. (1973). "Immunology and immunotherapy of cerebral tumors. Current status." Revue Neurologique (Paris) **128**(1): 23-38.

Trouillas, P. and C. Lapras (1970). "[Active immunotherapy of cerebral tumor. 20 cases]. [French]." Neurochirurgie. **16**(2): 143-170.

Truckenmiller, M. E., R. H. Bonneau, et al. (2006). "Stress presents a problem for dendritic cells: corticosterone and the fate of MHC class I antigen processing and presentation." Brain Behav Immun **20**(3): 210-8.

Truckenmiller, M. E., M. F. Princiotta, et al. (2005). "Corticosterone impairs MHC class I antigen presentation by dendritic cells via reduction of peptide generation." J Neuroimmunol **160**(1-2): 48-60.

Tuohy, V. K., Z. J. Lu, et al. (1988). "A synthetic peptide from myelin proteolipid protein induces experimental allergic encephalomyelitis." Journal of Immunology **141**(4): 1126-1130.

van der Most, R. G., A. Currie, et al. (2006). "Cranking the immunologic engine with chemotherapy: using context to drive tumor antigen cross-presentation towards useful antitumor immunity." Cancer Res **66**(2): 601-4.

van Mierlo, G. J., Z. F. Boonman, et al. (2004). "Activation of dendritic cells that cross-present tumor-derived antigen licenses CD8+ CTL to cause tumor eradication." J Immunol **173**(11): 6753-9.

Velicu, S., Y. Han, et al. (2006). "Cross-priming of T cells to intracranial tumor antigens elicits an immune response that fails in the effector phase but can be augmented with local immunotherapy." J Neuroimmunol **174**(1-2): 74-81.

Vincenti, F., R. Kirkman, et al. (1998). "Interleukin-2-receptor blockade with daclizumab to prevent acute rejection in renal transplantation. Daclizumab Triple Therapy Study Group.[see comment]." New England Journal of Medicine **338**(3): 161-5.

Wahl, S. M., D. A. Hunt, et al. (1988). "Transforming growth factor-beta is a potent immunosuppressive agent that inhibits IL-1-dependent lymphocyte proliferation." Journal of Immunology **140**(9): 3026-3032.

Wahlstrom, T., E. Linder, et al. (1973). "Glia-specific antigens in cell cultures from rabbit brain, human foetal and adult brain, and gliomas." Acta Pathologica et Microbiologica Scandinavica [B] Microbiology and Immunology **81**(6): 768-774.

Waksman, B. H., H. Porter, et al. (1954). "A study of the chemical nature of components of bovine white matter effective in producing allergic encephalomyelitis in the rabbit." Journal of Experimental Medicine **100**: 451-471.

Waldmann, T. A. (2006). "The biology of interleukin-2 and interleukin-15: implications for cancer therapy and vaccine design." Nature Reviews Immunology **6**(8): 595-601.

Waldmann, T. A., W. C. Greene, et al. (1984). "Functional and phenotypic comparison of human T cell leukemia/lymphoma virus positive adult T cell leukemia with human T cell leukemia/lymphoma virus negative Sezary leukemia, and their distinction using anti-Tac. Monoclonal antibody identifying the human receptor for T cell growth factor." Journal of Clinical Investigation **73**(6): 1711-8.

Waldmann, T. A., J. D. White, et al. (1993). "The interleukin-2 receptor: a target for monoclonal antibody treatment of human T-cell lymphotrophic virus I-induced adult T-cell leukemia." Blood **82**(6): 1701-12.

Walker, M. D., E. Alexander, Jr, et al. (1978). "Evaluation of BCNU and/or radiotherapy in the treatment of anaplastic gliomas. A cooperative clinical trial." Journal of Neurosurgery **49**(3): 333-343.

Walker, M. D., S. B. Green, et al. (1980). "Randomized comparisons of radiotherapy and nitrosoureas for the treatment of malignant glioma after surgery." New England Journal of Medicine **303**(23): 1323-1329.

Weinstein, I. B. (2002). "Cancer. Addiction to oncogenes--the Achilles heal of cancer." Science **297**(5578): 63-4.

Wekerle, H., K. Kojima, et al. (1994). "Animal models." Annals of Neurology **36 Suppl**: S47-53.
Westphal, M., D. C. Hilt, et al. (2003). "A phase 3 trial of local chemotherapy with biodegradable carmustine (BCNU) wafers (Gliadel wafers) in patients with primary malignant glioma." Neuro-oncology **5**: 79-88.
Wheeler, C. J., A. Das, et al. (2004). "Clinical responsiveness of glioblastoma multiforme to chemotherapy after vaccination." Clin Cancer Res **10**(16): 5316-26.
Wickremesinghe, H. R. and P. O. Yates (1971). "Immunological properties of neoplastic neural tissues." British Journal of Cancer **25**(4): 711-720.
Wikstrand, C. J. and D. D. Bigner (1979). "Surface antigens of human glioma cells shared with normal adult and fetal brain." Cancer Res. **39**(8): 3235-3243.
Wikstrand, C. J. and D. D. Bigner (1981). "Hyperimmunization of non-human primates with BCG-CW and cultured human glioma-derived cells. Production of reactive antisera and absence of EAE induction." Journal of Neuroimmunology **1**(3): 249-260.
Wikstrand, C. J., L. P. Hale, et al. (1995). "Monoclonal antibodies against EGFRvIII are tumor specific and react with breast and lung carcinomas and malignant gliomas." Cancer Research **55**(14): 3140-3148.
Wikstrand, C. J., L. P. Hale, et al. (1995). "Monoclonal antibodies against EGFRvIII are tumor specific and react with breast and lung carcinomas and malignant gliomas." Cancer Res **55**(14): 3140-8.
Wikstrand, C. J., L. P. Hale, et al. (1995). "Monoclonal antibodies against EGFRvIII are tumor specific and react with breast and lung carcinomas and malignant gliomas." Cancer Research **55**(14): 3140-8.
Willerford, D. M., J. Chen, et al. (1995). "Interleukin-2 receptor alpha chain regulates the size and content of the peripheral lymphoid compartment." Immunity **3**(4): 521-30.
Williams, M. A., A. J. Tyznik, et al. (2006). "Interleukin-2 signals during priming are required for secondary expansion of CD8+ memory T cells." Nature **441**(7095): 890-3.
Wolkers, M. C., N. Brouwenstijn, et al. (2004). "Antigen bias in T cell cross-priming." Science **304**(5675): 1314-7.
Wong, A. J., S. H. Bigner, et al. (1987). "Increased expression of the epidermal growth factor receptor gene in malignant gliomas is invariably associated with gene amplification." Proceedings of the National Academy of Sciences of the United States of America **84**(19): 6899-6903.
Wong, A. J., J. M. Ruppert, et al. (1992). "Structural alterations of the epidermal growth factor receptor gene in human gliomas." Proceedings of the National Academy of Sciences of the United States of America **89**: 2965-2969.
Woodburn, J. R. (1999). "The epidermal growth factor receptor and its inhibition in cancer therapy." Pharmacol Ther **82**(2-3): 241-50.
Wrzesinski, C. and N. P. Restifo (2005). "Less is more: lymphodepletion followed by hematopoietic stem cell transplant augments adoptive T-cell-based anti-tumor immunotherapy." Current Opinion in Immunology **17**: 195-201.

Wu, A. H., J. Xiao, et al. (2006). "Identification of EGFRvIII-derived CTL epitopes restricted by HLA A0201 for dendritic cell based immunotherapy of gliomas." J Neurooncol **76**(1): 23-30.

Yamagiwa, S., J. D. Gray, et al. (2001). "A role for TGF-beta in the generation and expansion of CD4+CD25+ regulatory T cells from human peripheral blood." Journal of Immunology **166**(12): 7282-9.

Young, H. F. and A. M. Kaplan (1976). "Cellular immune deficiency in patients with glioblastoma." Surgical Forum **27**(62): 476-478.

Yu, J. S., C. J. Wheeler, et al. (2001). "Vaccination of malignant glioma patients with peptide-pulsed dendritic cells elicits systemic cytotoxicity and intracranial T-cell infiltration." Cancer Research **61**(3): 842-7.

Zelenay, S., J. Demengeot, et al. (2006). "Comment on "Cutting edge: anti-CD25 monoclonal antibody injection results in the functional inactivation, not depletion, of CD4+CD25+ T regulatory cells".[comment]." Journal of Immunology **177**(4): 2036-7; author reply 2037-8.

Zhao, D. M., A. M. Thornton, et al. (2006). "Activated CD4+CD25+ T cells selectively kill B lymphocytes." Blood **107**(10): 3925-32.

Zhou, J., M. E. Dudley, et al. (2005). "Persistence of multiple tumor-specific T-cell clones is associated with complete tumor regression in a melanoma patient receiving adoptive cell transfer therapy." Journal of Immunotherapy with Emphasis on Tumor Immunology **28**: 53-62, 2005 Jan-Feb.

Zimmermann, V. S., A. Bondanza, et al. (2004). "TNF-alpha coupled to membrane of apoptotic cells favors the cross-priming to melanoma antigens." J Immunol **172**(4): 2643-50.

Zuber, P., M. C. Kuppner, et al. (1988). "Transforming growth factor-beta 2 down-regulates HLA-DR antigen expression on human malignant glioma cells." European Journal of Immunology **18**(10): 1623-1626.
